# Antifungal and Profungal Potentials of Acetone-Extracted Complexes of Lichen Substances on Macromycete *Heterobasidion parviporum* Mycelium Growth Dynamics—The First In Vitro Approach

**DOI:** 10.3390/molecules31162882

**Published:** 2026-08-18

**Authors:** Łukasz Furmanek, Sławomir Bakier, Paweł Czarnota, Ireneusz Kapusta, Tomasz Pawłowicz, Mark R. D. Seaward, Piotr Borowik, Tomasz Oszako

**Affiliations:** 1 Department of Forest Protection, Forest Research Institute, Sękocin Stary, Braci Leśnej 3 Street, 05-090 Raszyn, Poland; pborow@poczta.onet.pl (P.B.); t.oszako@ibles.waw.pl (T.O.); 2 Institute of Forest Sciences, Faculty of Civil Engineering and Environmental Sciences, Białystok University of Technology, Wiejska 45E Street, 15-351 Białystok, Poland; s.bakier@pb.edu.pl (S.B.); tomasz.pawlowicz@pb.edu.pl (T.P.); 3 Department of Ecology & Environmental Protection, University of Rzeszów, Zelwerowicza 4 Street, 35-601 Rzeszów, Poland; pczarnota@ur.edu.pl; 4 Department of Food Technology and Human Nutrition, University of Rzeszów, Zelwerowicza 4 Street, 35-601 Rzeszów, Poland; ikapusta@ur.edu.pl; 5 School of Archaeological & Forensic Sciences, University of Bradford, Bradford BD7 1DP, UK; m.r.d.seaward@bradford.ac.uk

**Keywords:** chemical hormesis, lichen metabolites, pathogenic fungus, inhibition, usnic acid

## Abstract

This study provides the first experimental evidence on how acetone-extracted lichen substances affect the mycelial growth of the pathogenic basidiomycete *Heterobasidion parviporum*. Six lichen species were tested: the terricolous species *Cladonia arbuscula*, *C. digitata*, *C. rangiferina*, *C. uncialis* and *Cetraria islandica*, and the epiphytic species *Pseudevernia furfuracea*. Extracts were prepared from single species and from species mixtures, including *C. arbuscula* + *C. rangiferina* + *C. digitata* and all five terricolous species. The substantial economic losses caused by the tested pathogen in forestry provided the rationale for these experiments. Two supplementation methods were used: applying extracts (1–4 mL) to solidified agar and incorporating extracts into the medium before solidification. The effects varied by extract, dose and supplementation method, showing either inhibitory or stimulatory potential. Surface application generally caused stronger inhibition than incorporation into the liquid medium, in which growth stimulation occurred, especially with extracts of *C. rangiferina*, *C. digitata* and *Cetraria islandica*. Fungistatic effects were associated mainly with usnic acid and, synergistically, with physodic acid, chloroatranorin and atranorin. The results highlight lichen extract complexes as promising eco-friendly biocontrol agents against *H. parviporum* in forest nurseries and spruce stands, with possible ecological implications.

## 1. Introduction

The biosynthesis of secondary lichen metabolites depends on products of primary metabolism [1,2] and on the complex relationship between the mycobiont and photobiont known as lichen symbiosis, which generates qualitative profiles of secondary metabolites that are mostly unique in the natural world [3].

Primary metabolites, such as proteins, sugars and vitamins, are mainly stored intracellularly [2]. By contrast, secondary compounds are synthesized by the fungal component from the conversion of polyols produced during photosynthesis by the photobiont [4] and are transported extracellularly to fungal cells [2]. Secondary lichen metabolites are formed mainly through three biosynthetic pathways. Most are produced through the polyketide, or acetate–polymalonate, pathway, which gives rise to depsides and depsidones, as well as less numerous anthraquinones, dibenzofurans, including usnic acid derivatives, and higher aliphatic fatty acids [2,4,5]. Most polyketides are classified as phenolic compounds with an aromatic acid ring to which at least one hydroxyl group is attached [6]. Less common metabolites include branched compounds derived from the mevalonic acid pathway and shikimic-acid-derived pulvinic acid derivatives [2,4].

The current list of secondary lichen metabolites includes more than 1000 compounds [7,8]. Their biosynthesis and accumulation occur under the influence of biotic stress, including thallus size and the presence of lichenicolous fungi [9,10], and abiotic factors, including seasonal differences, temperature and precipitation [11,12]. The intensity of stress affects the concentration of secondary metabolites in the lichen thallus, which usually reaches low mass values [13,14]. However, these compounds may sometimes constitute a significant percentage of thallus dry mass [15,16,17], and the content of one dominant metabolite in the compound complex may exceed 50% [17].

Primary compounds are generally regarded as relatively soluble in both polar solvents, including water [16,18], and organic solvents, e.g., acetone [18]. By contrast, many secondary metabolites have poorer water solubility, as shown for usnic acid and for the effect of water pH on extraction efficiency [13,18,19]. At the same time, increasing evidence indicates that extraction with water of specific physicochemical parameters can be effective, including extraction with rainwater under laboratory conditions [16], which confirms earlier findings in this field [20,21].

Organic solvents are considered dominant and effective extraction media for secondary lichen metabolites [18,22,23]. However, more extensive studies on the solubility of polyphenols in acetone have also shown limited solubility when compared with rainwater extracts [16,24].

Regardless of the qualitative and quantitative profiles of the dissolved lichen-substance complex, the biological potential of an extract depends on the overall interaction among all components [13,25,26,27]. Such extracts may exhibit antifungal activity against macromycetes and against *Aspergillus* and *Fusarium* species [25,28,29], antibacterial activity [30,31], and antiviral properties [32]. These activities are relevant to pharmacology, including nanomedicine [33], and to medicine, including anticancer and neuroprotective applications [34]. The potential use of lichen substances in environmental protection and green chemistry, including corrosion inhibition, natural dyes and nanomaterials, has also been reported [35,36,37].

The biological potential of lichen extracts against the mycelium of macromycete species remains poorly understood, despite research in this area dating back to the mid-20th century [25]. Critical analysis of known experiments shows a highly diverse response profile in this group of fungi [25]. In general, fungistatic properties are most commonly reported for lichen metabolites, largely because of the research methodologies used [22]. However, under certain circumstances, stimulatory effects on mycelial growth have also been observed [16,25], indicating the potential use of extracted lichen substances as biostimulants in environmental protection, forestry and agriculture [38], similarly to plant biostimulants [39,40,41]. Growing interest in such applications may also have practical and economic relevance [39].

*Heterobasidion parviporum* Niemelä & Korhonen (Bondarzewiaceae, Basidiomycota) is a well-known pathogenic fungus of economic importance [42,43,44] that occurs in forests of Europe and Asia [45,46,47,48]. It most commonly infects the roots of spruce trees (*Picea* spp.) [49,50], and less frequently pine trees (*Pinus* spp.) and fir trees (*Abies* spp.) [51], causing white rot [46,52]. Current prevention of pathogen spread relies on a narrow range of biological and chemical substances used for stump treatment [53]. The scale of forestry losses, estimated at EUR 800 million in Europe alone [42], is sufficient reason to seek effective ways to reduce its negative biological impacts. However, the possible wider use of lichen substances has received insufficient attention in this research area. The only experiments to date involving lichen metabolites, now mainly of historical significance, were conducted on *Heterobasidion annosum* s. lat. [25,54,55,56,57,58,59]. Given later taxonomic changes that distinguished *H. parviporum* s. str. from this species complex, it is not certain whether the fungus tested in those studies was this species.

Root and butt rot caused by *H. parviporum* represents one of the key constraints to sustainable Norway spruce forestry in northern and boreal Europe, with substantial economic losses [42] and long-lasting impacts on stand stability, carbon storage and wood quality. Despite decades of research on *Heterobasidion* biology and its control [25], there remains a striking lack of knowledge about how secondary metabolites synthesized by lichens may modulate the growth, behaviour and competitive ability of this pathogen. By focusing on acetone extracts from ecologically relevant lichen species, our study addresses this gap and explores whether lichen-derived chemical diversity can translate into inhibitory or stimulatory effects on *H. parviporum* mycelial dynamics.

The aim of this article is to fill the research gap concerning the in vitro assessment of the potential of lichen-substance complexes against the mycelium of *H. parviporum*. Given the economic importance of this pathogen, as emphasized above, this research direction appears justified and, in light of recent in-depth studies on the biosynthetic potential of lichens [60], also promising.

For the experimental work, the following research hypotheses were adopted:

1. Lichen complexes dissolved in acetone have fungistatic effects against the tested fungal species.
2. The method of extract supplementation affects their antifungal properties.
3. The antifungal potential of lichen extracts depends on the lichen species used for extraction, indicating species-specific fungus–lichen interactions.
4. The potential of extracts obtained from mixed thalli of several lichen species differs from that of extracts obtained from thalli of single lichen species.

## 2. Results

The Supplementary Materials comprise Tables S1–S22 and Figures S1–S21.

The growth dynamics of *Heterobasidion parviporum* mycelium in extract-treated and control cultures are presented graphically in the Supplementary Materials (Figures S9–S21).

### 2.1. Control Assay with Acetone

#### 2.1.1. Supplementation onto the Medium Surface

Increasing doses of acetone consistently slowed mycelial growth compared with the solvent-free control. Mycelial growth was observed after 72 h at the 2 mL dose and after 216 h at the 3 mL dose. By the end of the experiment, at 312 h, when the substrate in the pure culture was completely overgrown, these differences were statistically significant. The mean mycelial diameter at 312 h was 65.3 mm in the solvent-free control, 40.3 mm in the culture treated with 1 mL of acetone, 39.5 mm in the culture treated with 2 mL of acetone, and 13.1 mm in the culture treated with 3 mL of acetone. No mycelial growth was observed in cultures treated with 4 or 5 mL of acetone. Differences in mycelial diameter among cultures treated with different acetone doses were not statistically significant (Table S2).

#### 2.1.2. Supplementation into the Liquid Medium

Mycelial growth dynamics in all acetone-treated control cultures were reduced relative to the solvent-free control, but these differences were not statistically significant. However, significant differences in mycelial diameter were observed between cultures treated with 1 and 3 mL of acetone and between those treated with 3 and 4 mL of acetone (Table S11).

### 2.2. Assay with Cladonia arbuscula Extract

#### 2.2.1. Supplementation onto the Medium Surface

The growth dynamics of mycelium on media treated with 1 and 2 mL of extract were similar, but differed significantly from those in the solvent-free control for almost the entire experiment, except at 12 h and at 84–96 h for the 1 mL dose (Figure S9; Table S3). At 312 h, the mean mycelial diameter for both tested extract doses was close to 20 mm. The differences in mycelial diameter at the end of the experiment between equivalent doses of acetone and extract were 20.2 mm for 1 mL and 19.8 mm for 2 mL, with no significant differences across the full range of measurements (Table S20).

#### 2.2.2. Supplementation into the Liquid Medium

At all tested doses, the extract limited the growth of *H. parviporum* according to a dose-dependent pattern: the higher the dose, the stronger the inhibition. Mycelium in all extract-treated cultures grew more slowly than in both the solvent-free control and the acetone control (Figure S10). After 228 h, the mean mycelial diameters in cultures treated with increasing extract volumes were 38.1 mm at 1 mL, 30.3 mm at 2 mL, 29.6 mm at 3 mL, and 26.5 mm at 4 mL. Significant differences from the control were observed for the 2 mL dose at 48 h and for the 3 and 4 mL doses from 48 h onward. A significant difference was also found between the 1 and 4 mL extract doses at two measurement times, 72 and 132 h (Table S12). The differences in mycelial diameter between cultures treated with equivalent doses of extract and acetone were 16.6 mm for 1 mL, 30.7 mm for 2 mL, 34.2 mm for 3 mL, and 29.3 mm for 4 mL; the difference for the 3 mL dose was significant at 60 h (Table S21).

### 2.3. Assay with Cladonia rangiferina Extract

#### 2.3.1. Supplementation onto the Medium Surface

Both tested extract doses significantly reduced mycelial growth relative to the solvent-free control, with significant differences recorded at 24–48 h for the 1 mL dose and from 24 h onward for the 2 mL dose. No significant differences in mycelial diameter were found between the two extract doses (Figure S11; Table S4). Compared with the acetone control, the 1 mL extract dose non-significantly inhibited the growth of *H. parviporum* for c. 168 h. After 192 h, however, mycelium began to grow faster and, by the end of the experiment, had reached a larger diameter than in the corresponding acetone control. In the culture treated with 2 mL of extract, no growth was observed until c. 96 h and then growth remained clearly slower than in the control, although the differences were not statistically significant (Table S20). At 312 h, the mean mycelial diameter in the culture treated with 1 mL of extract was 49.1 mm, which was 8.8 mm greater than in the culture treated with an equivalent acetone dose. At the same time, the pathogen inoculum in the culture treated with 2 mL of extract reached 13.1 mm in diameter, which was 26.4 mm less than in the culture treated with the same dose of acetone.

#### 2.3.2. Supplementation into the Liquid Medium

The mycelial growth rate decreased with increasing extract dose and remained below that of the control. Significant differences in mycelial diameter were observed for the 2 mL dose at 36–72 h and for the 4 mL dose at 216–228 h. Significant differences were also found between the 2 and 4 mL doses from 48 h onward and between the 1 and 4 mL doses from 144 h onward (Figure S12; Table S13). Extract doses of 1–2 mL stimulated mycelial growth, whereas the 3 mL dose inhibited mycelial growth dynamics compared with an equivalent solvent volume. The culture treated with 4 mL of extract initially grew at a similar rate to the corresponding acetone control, but after approximately 132 h its growth rate became increasingly reduced. No paired comparison between equivalent extract and acetone doses showed significant differences in growth dynamics (Table S21). The largest mean mycelial diameters recorded after 228 h were 61.7 mm at 1 mL, 61.1 mm at 2 mL, 57.0 mm at 3 mL, and 52.4 mm at 4 mL.

### 2.4. Assay with Cladonia digitata Extract

#### 2.4.1. Supplementation onto the Medium Surface

All three tested extract doses reduced mycelial growth relative to the control. Significant differences in mycelial growth dynamics were confirmed for the 1 mL extract dose at 24 h and for the 2 and 3 mL extract doses from 24 h onward. Significant differences were also found between the 1 and 3 mL extract doses at 252–264, 288, and 312 h (Figure S13; Table S5). At 312 h, mycelial diameter reached 59.5 mm in the culture treated with 1 mL of extract, 26.9 mm with 2 mL, and 8.9 mm with 3 mL. The 1 mL extract treatment produced a mycelial diameter 19.2 mm greater than that in the culture treated with an equivalent acetone dose, whereas the 2 and 3 mL extract treatments produced diameters smaller by 12.6 and 3.2 mm, respectively. No significant differences were found between paired treatments with equivalent extract and solvent volumes (Table S20).

#### 2.4.2. Supplementation into the Liquid Medium

After 228 h, mycelial diameters were 58.5 mm at 1 mL, 61.0 mm at 2 mL, 56.8 mm at 3 mL, and 52.0 mm at 4 mL, with no significant differences in mycelial diameter overall. A significant difference in growth dynamics was observed between the 2 and 3 mL extract doses at 60, 84, and 108–120 h (Figure S14; Table S14). No significant differences were observed between cultures treated with equivalent doses of extract and acetone (Table S21). Mycelial growth was stimulated in cultures treated with 1 and 2 mL of extract for most measurement times, whereas the 3 mL dose inhibited mycelial growth. The 4 mL extract dose initially had a stimulatory effect, followed by inhibition.

### 2.5. Assay with Cladonia uncialis Extract

#### 2.5.1. Supplementation onto the Medium Surface

Mycelial growth was significantly reduced in the culture treated with 1 mL of extract, with statistically significant differences from the control recorded from 24 to 108 h (Figure S15; Table S6). No significant differences were found in comparison with the culture treated with 1 mL of acetone (Table S20). The mean mycelial diameter after 312 h was 12.3 mm, which was 28.0 mm smaller than that measured in the culture treated with 1 mL of acetone. Higher extract doses, 2 and 3 mL, completely inhibited mycelial growth, as confirmed by significant differences in mycelial diameter from the control from 24 h onward (Table S6). A significant difference in growth dynamics between the culture treated with 2 mL of extract and that treated with an equivalent solvent dose occurred from 192 h onward. The comparison between cultures treated with 3 mL of extract and 3 mL of acetone showed no statistically significant differences (Table S20).

#### 2.5.2. Supplementation into the Liquid Medium

The extract increased mycelial inhibition as the tested dose increased. Compared with the control, mycelium in all extract-treated cultures grew more slowly, with significant differences in growth dynamics recorded for the 3 mL dose from 108 h and for the 4 mL dose from 48 h onward (Figure S16; Table S15). No significant differences in mycelial diameter were found between cultures treated with equivalent doses of extract and acetone (Table S21), despite the reduction in growth dynamics below the level observed in the acetone-treated cultures. After 228 h, mean mycelial diameter was 38.4 mm at 1 mL, 36.2 mm at 2 mL, 32.1 mm at 3 mL, and 22.6 mm at 4 mL. These values were smaller than those measured in the acetone-treated cultures by 16.3, 24.8, 31.7, and 33.2 mm, respectively.

### 2.6. Assay with Cetraria islandica Extract

#### 2.6.1. Supplementation onto the Medium Surface

All tested extract volumes, 1, 2, and 3 mL, completely inhibited mycelial growth. Mycelial diameter differed significantly from that in the control from 24 h onward (Table S7) and from that in the equivalent acetone treatment from 192 h onward for the 2 mL extract dose (Table S20).

#### 2.6.2. Supplementation into the Liquid Medium

Compared with the control, mycelial growth dynamics were stimulated in all extract-treated cultures up to 84 h (Figure S17). In the culture treated with 1 mL of extract, growth stimulation was maintained throughout the experiment, whereas in the culture treated with 2 mL of extract it persisted up to 180 h. In cultures treated with 3 and 4 mL of extract, growth dynamics subsequently decreased below those of the solvent-free control. No significant differences in mycelial diameter were found, except for a single difference at 216 h between the cultures treated with 1 and 4 mL of extract (Table S16). Cultures treated with 1 and 2 mL of extract showed stimulated mycelial growth compared with cultures treated with equivalent acetone doses. Higher extract doses, 3 and 4 mL, initially stimulated growth but were followed by inhibition. No significant differences in mycelial growth dynamics were found between paired treatments with equivalent doses of extract and acetone (Table S21). At 228 h, mean mycelial diameter decreased with increasing extract dose and reached 66.4 mm at 1 mL, 60.2 mm at 2 mL, 58.4 mm at 3 mL, and 46.6 mm at 4 mL.

### 2.7. Assay with Extract from Mixed Thalli of Cladonia arbuscula, C. rangiferina, and C. digitata

#### 2.7.1. Supplementation onto the Medium Surface

The mycelium was susceptible to the tested extract doses. A significantly smaller mycelial diameter than in the solvent-free control was found at 24 h in the culture treated with 1 mL of extract and from 24 h onward in cultures treated with 2 and 3 mL of extract (Figure S18; Table S8). In the culture treated with 1 mL of extract, mycelium showed growth dynamics similar to those in the culture treated with 1 mL of acetone up to 132 h, after which its growth slowed. In the culture treated with 2 mL of extract, mycelial growth began after 132 h and remained clearly weaker than in the culture treated with an equivalent acetone dose, although this difference was not statistically significant (Table S20). The 3 mL extract dose completely inhibited mycelial growth. The mean mycelial diameter after 312 h was 29.6 mm in the culture treated with 1 mL of extract and 8.3 mm in the culture treated with 2 mL. The differences between equivalent extract and acetone doses were 10.7 and 31.2 mm, respectively.

#### 2.7.2. Supplementation into the Liquid Medium

Inhibition of mycelial growth dynamics was observed in all extract-treated cultures (Figure S19). Compared with the control, the culture treated with 2 mL of extract grew significantly more slowly from 84 to 144 h and from 168 to 216 h. Mycelial diameter in cultures treated with 3 and 4 mL of extract was significantly smaller from 48 h onward. A significant difference in mycelial diameter between the 1 and 4 mL extract doses occurred at 60 h (Table S17). Compared with the acetone-treated cultures, the 3 mL extract treatment significantly inhibited growth dynamics at 228 h (Table S21). At 228 h, the mean mycelial diameter was 41.0 mm at 1 mL, 33.0 mm at 2 mL, 31.5 mm at 3 mL, and 30.8 mm at 4 mL. These values represented reductions in mycelial diameter relative to the corresponding acetone-treated cultures of 13.7, 28.0, 32.3, and 25.0 mm, respectively.

### 2.8. Assay with Extract from Mixed Thalli of Cladonia arbuscula, C. rangiferina, C. digitata, C. uncialis, and Cetraria islandica

#### 2.8.1. Supplementation onto the Medium Surface

All tested extract volumes, 1, 2, and 3 mL, completely inhibited mycelial growth. Mycelial diameter differed significantly from that in the control from 24 h onward (Table S9) and from that in the equivalent acetone treatment from 192 h onward for the 2 mL extract dose (Table S20).

#### 2.8.2. Supplementation into the Liquid Medium

All extract-treated cultures showed reduced mycelial growth dynamics (Figure S20). Compared with the control, significant inhibition occurred in cultures treated with 3 and 4 mL of extract from 48 h onward. In addition, significant differences in mycelial diameter between cultures treated with 1 and 4 mL of extract were recorded from 60 to 192 h and from 216 to 228 h (Table S18). At 228 h, the mean mycelial diameter was 43.4 mm at 1 mL, 38.5 mm at 2 mL, 31.9 mm at 3 mL, and 27.5 mm at 4 mL. Comparisons between cultures treated with equivalent doses of extract and acetone showed no significant differences in mycelial growth dynamics (Table S21). The differences in mean mycelial diameter between paired extract and acetone treatments were 11.3 mm at 1 mL, 22.5 mm at 2 mL, 31.9 mm at 3 mL, and 28.3 mm at 4 mL.

### 2.9. Assay with Pseudevernia furfuracea Extract

#### 2.9.1. Supplementation onto the Medium Surface

All tested extract doses, 1, 2, and 3 mL, completely inhibited the growth of *H. parviporum* mycelium throughout the entire cultivation period (Table S10). In the solvent-free control, isolate growth began as early as 24 h and reached 65.3 mm in diameter at the end of the experiment. In acetone-treated control cultures, isolate growth began later and later with increasing acetone dose, at 48, 84, and 228 h, respectively, with statistically significant differences in diameter after 192 h in cultures treated with 2 mL of acetone (Table S20).

#### 2.9.2. Supplementation into the Liquid Medium

All extract-treated cultures showed reduced growth dynamics of *H. parviporum* mycelium (Figure S21). The 3 and 4 mL extract doses significantly inhibited growth compared with the solvent-free control from 48 h onward. In addition, a significant difference in mycelial diameter between cultures treated with 1 and 4 mL of extract occurred after 108 h of cultivation (Table S19). Comparisons between cultures treated with equivalent doses of extract and acetone showed significant inhibition of mycelial growth at the 2 mL extract dose from 96 to 108 h and from 132 to 228 h, at the 3 mL dose from 60 h to the end of the experiment, and at the 4 mL dose from 84 h onward (Table S21). The differences in mean mycelial diameter between equivalent extract and acetone doses were 27.6, 46.6, 53.2, and 49.1 mm for doses of 1, 2, 3, and 4 mL, respectively.

### 2.10. Statistical Comparison Among Extracts Obtained from Different Lichen Species

#### 2.10.1. Supplementation onto the Medium Surface

Statistical analyses were also performed between paired cultures supplemented with corresponding doses of the tested lichen extracts. For cultures treated with 1 mL of extract, significant differences in mycelial growth dynamics were most frequently associated with extract from *Cladonia digitata* thalli. Mycelium supplemented with *C. digitata* extract grew significantly faster than mycelium treated with extracts obtained from *C. rangiferina*, *C. uncialis*, *Cetraria islandica*, *Pseudevernia furfuracea*, and mixed thalli of five lichen species (Table S20). No statistically significant differences in mycelial diameter were found among cultures treated with 2 or 3 mL of the tested extracts.

#### 2.10.2. Supplementation into the Liquid Medium

For cultures treated with 1 mL of extract, differences in mycelial growth dynamics were most frequently associated with extract from *Pseudevernia furfuracea* thalli. Cultures supplemented with *P. furfuracea* extract showed significant inhibition of mycelial growth compared with cultures treated with extracts from *Cladonia arbuscula*, *C. rangiferina*, *C. digitata* and *Cetraria islandica*, and the extract obtained from mixed thalli of three lichen species. The culture treated with 2 mL of *P. furfuracea* extract also showed a significant reduction in growth dynamics compared with the culture treated with extract from mixed thalli of five lichen species. At the 3 mL dose, *P. furfuracea* extract significantly inhibited mycelial growth dynamics in the same way as at the 1 mL dose, except when compared with extract from mixed thalli of three lichen species. A similar pattern was observed at the 4 mL dose: mycelium grown with *P. furfuracea* thallus extract showed significant inhibition of growth dynamics compared with cultures treated with extracts from *C. arbuscula*, *C. rangiferina*, *C. digitata*, and *Cetraria islandica* (Table S21).

### 2.11. Statistical Comparison of Mycelial Growth Dynamics Between the Two Supplementation Methods

Mycelium growing in the control culture under conditions optimized for supplementation onto the medium surface showed significant stimulation of growth dynamics during the initial growth phase. In contrast, mycelium growing in the control culture under conditions optimized for supplementation into the medium before solidification showed stimulated growth dynamics during the later phase of cultivation (Table S22A).

Comparison of mycelial growth dynamics in cultures treated with tested doses of acetone and extract showed significant stimulation in all cases when acetone or extract was supplemented into the medium before solidification, compared with cultures treated with equivalent doses of acetone or extract supplemented onto the solidified agar surface. Extract doses of 3 mL from *Cladonia arbuscula* and *C. rangiferina* thalli were not tested (Table S22B).

## 3. Discussion

### 3.1. Growth Dynamics of H. parviporum Mycelium in Control Samples Without Acetone

Control cultures of *Heterobasidion parviporum* isolates on unsupplemented Hansen’s medium were prepared independently at different times, under nominally identical laboratory conditions, as reference controls for two extract-supplementation variants: supplementation onto the surface of solidified medium and supplementation into the medium before solidification. These controls did not show identical growth dynamics. During the initial and final phases of mycelial growth, differences in mean colony diameter were even statistically significant, and the direction of these differences changed over time (Table S22A). This discrepancy was most likely caused by uncontrolled cultivation-related factors, such as the time of day and season at which isolates were inoculated, the physiological condition of the isolates themselves, and fluctuations in temperature and humidity in the mycological laboratory [61]. This interpretation is supported by the larger standard deviations recorded for cultures used as controls in the experiments in which extracts were supplemented into the medium.

### 3.2. Growth Dynamics of H. parviporum Mycelium in Control Samples with Acetone

The growth dynamics of *H. parviporum* in control samples with acetone supplemented onto the surface of the medium (Table S2) was markedly weaker than in the control sample without acetone and differed from the control samples in which acetone was mixed with liquid medium before solidification (Table S11). Despite prior evaporation of the solvent from the surface of the medium, higher acetone doses had a statistically significant effect; doses of 4 and 5 mL completely prevented measurable mycelial growth during the assay, although fungicidal activity was not tested.

A different response was observed when acetone was mixed with the liquid medium before solidification. In this case, no statistically significant differences were found relative to the control sample without acetone. It appears that sterilisation of the medium in a steam autoclave promoted a faster solvent evaporation and, consequently, a reduction in the negative effect of acetone on pathogen growth, although residual acetone was not quantified.

In contrast to the experiments in which acetone was supplemented onto the medium, the tests with 2 and 3 mL of acetone mixed into the medium showed slight stimulation of mycelial growth compared with the control without acetone. This response may reflect chemical hormesis, a phenomenon previously considered in studies using lichen thallus extracts against macrofungi [25].

All control samples with acetone mixed into the medium showed similar, statistically insignificant growth dynamics relative to the control sample without acetone (Table S11). Thus, the unknown factors discussed above as possible causes of significantly different growth dynamics in controls without acetone (Table S22A) seem to be controlled sufficiently during the experiments. Although these factors remain unidentified, the adopted methodological rigour was adequate and supports the conclusions presented here.

The reduced effect of acetone on mycelial growth in control tests with acetone supplemented into the medium was also confirmed by statistical analysis comparing colony diameters between the two acetone-supplementation methods: onto solidified medium and into the medium before solidification. At most measurement times, colony diameters were significantly larger in controls with 1, 2 and 3 mL acetone mixed into the medium; a 4 mL acetone dose was not tested in the control variant in which acetone was supplemented onto the solidified medium (Table S22B). Thus, the effect of acetone evaporation during autoclave sterilisation on the growth rate was indirectly confirmed.

It cannot be excluded that the reduced effect of acetone on mycelial growth was also caused by other factors, including more uniform dispersion of acetone in the medium and consequently lower local concentrations at the surface of the solidified medium. The hormetic effects mentioned above also cannot be ruled out. Moisture, understood here as hydration of the culture medium, did not appear to limit the growth dynamics of the tested isolates when acetone was used, because *H. parviporum* mycelium grew faster in the control sample with acetone mixed into the medium.

On this basis, direct comparison of mycelial growth dynamics between samples prepared using the two different supplementation methods is limited. Nevertheless, the comparison provides conclusions that would not have been possible with a less differentiated experimental design.

### 3.3. Growth Dynamics of H. parviporum Mycelium Under Extract Supplementation onto and into the Medium

The data clearly show that lichen extracts can exert both antifungal and profungal effects on *H. parviporum*, depending on lichen species, extract concentration and exposure time. Here, the term antifungal denotes a net reduction in growth rate, whereas profungal refers to stimulation of mycelial growth relative to the control samples. Such bidirectional responses indicate that lichen secondary metabolites do not act simply as generic toxins, but rather as context-dependent modulators of fungal physiology. This duality is ecologically important because it suggests that lichen-derived chemistry may fine-tune competitive relationships within wood-decaying fungal communities instead of uniformly suppressing potential pathogens.

### 3.4. Potential of Extracts from Cladonia arbuscula

Only trace concentrations of polyphenols were detected spectrophotometrically in acetone extracts from *Cladonia arbuscula* (Table 1), as also reported by Furmanek et al. [16]. Chromatographic analysis likewise indicated trace amounts of usnic acid (Table 2). In the acetone-extracted lichen-substance complex, which consisted of only a few compounds, usnic acid occurred at the highest concentration (Figure S1). This metabolite therefore most likely contributed substantially to the inhibition of *H. parviporum* mycelial growth.

When the extract was supplemented onto the surface of the solidified medium, the maximum relative reduction in pathogen growth reached 40–57 and 70–80 percentage points (p.p.) in relation to the control samples with and without acetone. When the extract was supplemented into the culture medium before solidification, mycelial growth inhibition ranged from 33–58 p.p. in relation to the control sample without acetone and from 19–59 p.p. in relation to the control samples with acetone. Thus, supplementation onto the solidified medium was more effective in terms of antifungal activity. In general, both supplementation methods produced a lower reduction in mycelial growth dynamics when the extract-treated samples were compared with controls containing an equivalent dose of acetone (Table 3 and Table 4). This comparison suggests that the solvent effect does not substantially alter the conclusions drawn from the analysis; this interpretation also applies to all other tested lichen extracts.

### 3.5. Potential of Extracts from Cladonia rangiferina

Only trace amounts of polyphenols were detected in extracts obtained from *Cladonia rangiferina* (Table 1). This result was unexpected because the concentration of these substances was several times lower than that reported for an acetone extract obtained using an identical procedure [16].

Although the extracted lichen-substance complex was chemically rich, only atranorin was putatively identified among the secondary metabolites of *C. rangiferina* (Figure S2). Fumarprotocetraric acid, which is listed in the literature among the main substances determining the chemistry of this species [63,64], was not detected (Table 2). Moreover, from the perspective of the chemical profile of the extracted lichen substances, atranorin was not dominant, in contrast to the profile obtained from *C. arbuscula* (Figures S1 and S2).

When supplemented onto the solidified medium, the extracted lichen-substance complex inhibited *H. parviporum* mycelial growth in the range of c. 25–82 p.p. compared with the control without acetone. However, compared with control samples containing an equivalent acetone dose, the mycelial growth dynamics showed not only a smaller reduction (max. c. −67 p.p.), but also proved to be stimulated (c. +22 p.p.) (Table 3).

When the extract was supplemented into the medium before solidification, stimulation of mycelial growth was even more evident (Table 3 and Table 4). Relative to the control without acetone, the reduction in growth dynamics reached a maximum of 16 p.p. (for the highest tested dose), while at the same time showing a stimulation effect to a maximum level of c. +12 p.p. at the 2 mL extract dose (Table 4). Compared with controls containing an equivalent acetone dose, an even smaller degree of reduction in mycelial growth dynamics was demonstrated (max. c. −11 p.p.), together with a greater level of mycelial growth stimulation (max. c. +22 p.p.). Test samples supplemented with 1 and 2 mL of extract therefore showed an essentially stimulatory effect on mycelial growth dynamics (Table 4).

**Table 3:** Effect of acetone-extracted lichen extracts supplemented onto the surface of solidified Hansen’s medium at different doses on the growth of *Heterobasidion parviporum* mycelium after specified experimental periods. Values are expressed as percentages of the mycelial diameter in the relevant control sample. Values above 100% indicate stimulation relative to the corresponding control.

Extract Source	Reference for Comparison	1 mL		2 mL		3 mL	
		156 h	312 h	156 h	312 h	156 h	312 h
*Cladonia arbuscula*	Without Acetone	21.5	30.8	19.1	30.2	–	
*Cladonia rangiferina*	Without Acetone	43.8	75.3	17.6	20.1	–	
*Cladonia digitata*	Without acetone	74.4	91.1	16.5	41.2	*	13.6
*Cladonia uncialis*	Without acetone	16.5	18.8	*	*	*	*
*Cetraria islandica*	Without acetone	*	*	*	*	*	*
Three-species mixture	Without acetone	43.8	45.3	15.9	12.7	*	*
Five-species mixture	Without acetone	*	*	*	*	*	*
*Pseudevernia furfuracea*	Without acetone	*	*	*	*	*	*
*Cladonia arbuscula*	Equivalent acetone dose	42.7	49.9	60.2	49.9	–	
*Cladonia rangiferina*	Equivalent acetone dose	87.1	121.8	55.5	33.2	–	
*Cladonia digitata*	Equivalent acetone dose	147.9	147.6	51.8	68.1	*	73.5
*Cladonia uncialis*	Equivalent acetone dose	32.7	30.5	*	*	*	*
*Cetraria islandica*	Equivalent acetone dose	*	*	*	*	*	*
Three-species mixture	Equivalent acetone dose	87.1	73.4	50.0	21.0	*	*
Five-species mixture	Equivalent acetone dose	*	*	*	*	*	*
*Pseudevernia furfuracea*	Equivalent acetone dose	*	*	*	*	*	*

Notes: Asterisks indicate no measurable increase in average mycelial diameter; dashes indicate treatments not tested. The three-species mixture consisted of *Cladonia arbuscula*, *C. rangiferina* and *C. digitata*. The five-species mixture consisted of *Cladonia arbuscula*, *C. rangiferina*, *C. digitata*, *C. uncialis* and *Cetraria islandica*.

**Table 4:** Effect of acetone-extracted lichen extracts supplemented into Hansen’s medium before solidification at different doses on the growth of *Heterobasidion parviporum* mycelium after specified experimental periods. Values are expressed as percentages of the mycelial diameter in the relevant control sample. Values above 100% indicate stimulation relative to the corresponding control.

Extract Source	Reference for Comparison	1 mL		2 mL		3 mL		4 mL	
		120 h	228 h	120 h	228 h	120 h	228 h	120 h	228 h
*Cladonia arbuscula*	Without acetone	66.5	61.0	52.8	48.5	44.5	47.4	41.8	42.5
*Cladonia rangiferina*	Without acetone	100.4	98.9	111.8	97.9	99.2	91.3	82.9	84.0
*Cladonia digitata*	Without acetone	97.3	93.7	108.4	97.7	87.8	91.0	90.1	83.3
*Cladonia uncialis*	Without acetone	66.9	61.5	63.5	58.0	55.1	51.4	36.5	36.2
*Cetraria islandica*	Without acetone	114.1	106.4	110.3	96.5	112.9	93.6	90.5	74.7
Three-species mixture	Without acetone	74.1	65.7	55.5	52.9	56.6	50.5	53.2	49.3
Five-species mixture	Without acetone	76.4	69.5	63.9	61.7	56.6	51.1	51.7	43.4
*Pseudevernia furfuracea*	Without acetone	51.7	43.4	28.5	23.1	22.0	17.0	*	10.7
*Cladonia arbuscula*	Equivalent acetone dose	81.0	69.6	53.2	49.7	40.5	46.4	51.2	47.5
*Cladonia rangiferina*	Equivalent acetone dose	122.2	112.8	112.6	100.2	90.3	89.3	101.4	93.9
*Cladonia digitata*	Equivalent acetone dose	118.5	106.9	109.2	100.0	79.9	89.0	110.2	93.2
*Cladonia uncialis*	Equivalent acetone dose	81.5	70.2	64.0	59.3	50.2	50.3	44.6	40.5
*Cetraria islandica*	Equivalent acetone dose	138.9	121.4	111.1	98.7	102.8	91.5	110.7	83.5
Three-species mixture	Equivalent acetone dose	90.3	74.9	55.9	51.1	51.5	49.4	65.1	55.2
Five-species mixture	Equivalent acetone dose	93.0	79.3	64.4	63.1	51.5	50.0	58.1	49.3
*Pseudevernia furfuracea*	Equivalent acetone dose	63.0	49.5	28.7	23.6	20.1	16.6	*	12.0

Notes: Asterisks indicate no measurable increase in average mycelial diameter. The three-species mixture consisted of *Cladonia arbuscula*, *C. rangiferina* and *C. digitata*. The five-species mixture consisted of *Cladonia arbuscula*, *C. rangiferina*, *C. digitata*, *C. uncialis* and *Cetraria islandica*.

### 3.6. Potential of Extracts from Cladonia digitata

The total content of determined polyphenols in extracts from *Cladonia digitata* thalli was higher than in extracts from *C. arbuscula* and *C. rangiferina* (Table 1) and was comparable to the concentration reported for an acetone extract reported by [16]. Chromatographic analysis did not reveal the presence of thamnolic acid, the secondary metabolite characteristic of this species and reported, for example, by Smith et al. [63] (Table 2; Figure S3). Nevertheless, the dissolved lichen-substance complex was relatively rich (Figure S3).

Despite the absence of thamnolic acid, the extracted lichen complexes supplemented onto the solidified medium showed a broad range of inhibitory potential. Relative to the control without acetone, mycelial growth dynamics showed a reduction ranging from c. 9 p.p. at the 1 mL extract dose to a complete reduction in growth at the 3 mL dose. Conversely, as in the case of the *C. rangiferina* extract, the biological potential of the extract relative to the control with an equivalent acetone dose also included stimulation of mycelial growth. The response ranged from complete inhibition at the 3 mL dose to a stimulation effect of c. +48 p.p. at the 1 mL dose (Table 3).

The 3 mL extract dose caused a marked delay in the onset of measurable mycelial growth, which was observed only during the second half of the culture period. This indicates, at least in this case, that the lichen substances present in the extract may exert a prolonged inhibitory effect on pathogen mycelial growth, although not necessarily a fungicidal effect (Table 3).

When the *C. digitata* extract was supplemented into the medium before solidification, a phenomenon similar to that observed for *C. arbuscula* and *C. rangiferina* extracts was recorded: a smaller reduction in growth dynamics relative to the control without acetone, reaching a maximum of c. 20 p.p. at the 4 mL dose, and more frequent stimulation relative to samples with an equivalent acetone dose, reaching a maximum of +18 p.p. at the 1 mL dose (Table 3 and Table 4).

Although the positive effect of *C. digitata* extracts supplemented into liquid medium on *H. parviporum* mycelial growth decreased over time, the profungal effects were observed across a wider range of extract doses. In addition, in samples supplemented with 4 mL of extract, the mid-culture stimulation of mycelial growth was ultimately reduced to slight inhibition. This indicates that supplementation with a higher extract dose does not necessarily produce a stronger limitation of pathogen growth dynamics (Table 4). A similar, although less pronounced, pattern was observed for *C. rangiferina* extracts at 3 and 4 mL doses (Table 4).

### 3.7. Potential of Extracts from Cladonia uncialis

Spectrophotometric determination showed that the total polyphenol content in the tested extracts was at the limit of quantification or measurement error, or was absent altogether in the extract supplemented into the medium (Table 1). Compared with the concentration of substances in an extract obtained using an identical method [16], this result was markedly lower, although still within a generally low range of substance content. Nevertheless, in the extract supplemented into the liquid medium, chromatographic analysis indicated a modest qualitative metabolite profile with a signal putatively assigned to usnic acid (Figure S4A,B; Table 2).

It is therefore likely that the trace but dominant concentration of usnic acid, whose antifungal activity has been repeatedly reported [25], was the main determinant strongly inhibiting *H. parviporum* mycelial growth, also in samples in which the extract was supplemented onto the solidified medium. Relative to the control without acetone, the reduction of mycelial growth at the 1 mL dose of *C. uncialis* extract was c. 81–87 p.p. Relative to the control with an equivalent 1 mL acetone dose, this reduction decreased to c. 67–69 p.p. Doses of 2 and 3 mL of the same extract completely prevented measurable mycelial growth (Table 3).

Mycelium growing in samples with *C. uncialis* extract mixed with the medium before solidification, as in the case of extracts obtained from other lichen species, grew significantly better than in samples in which the extract was supplemented onto the medium. Relative to the control without acetone, the highest reduction was c. 33–64 p.p. and relative to controls with equivalent acetone doses, it ranged from c. 18–59 p.p. (Table 4). In contrast to the complete inhibition of mycelial growth in samples with 2 and 3 mL of extract supplemented onto the medium, supplementation into the medium enabled growth at equivalent doses, although growth remained limited (Table 3 and Table 4).

### 3.8. Potential of Extracts from Cetraria islandica

The response of *H. parviporum* mycelium to extracts obtained from *Cetraria islandica* was among the most interesting results of the experiments assessing the effects of lichen substances from individual lichen species.

Spectrophotometric analysis of acetone extracts produced a negative result (Table 1). Protolichesterinic acid was putatively identified chromatographically in these extracts (Table 2), although it was not dominant in the poor complex of extracted lichen substances (Figure S5A,B). The absence of detectable polyphenols synthesised by *C. islandica* is consistent with the spectrophotometric analysis reported by Furmanek et al. [16]. Interestingly, the results obtained by Tekiela et al. [64] did not confirm any of the secondary metabolites known from the literature as components of the chemical profile of *C. islandica* [63]. This may indicate, among other things, the influence of environmental factors on their biosynthesis, e.g., altitude [65] and soil conditions [66]. The uniqueness of extracts from *C. islandica* also results from the chromatographic signal putatively assigned to protolichesterinic acid, which belongs to the class of higher aliphatic fatty acids (Table 1 and Table 2) [5] and is poorly soluble in acetone [67]. It is possible that, instead of protolichesterinic acid, a derivative or isomer of this compound, lichesterinic acid, was extracted; the two compounds have identical molecular weights [5,62,68].

When *C. islandica* extract was supplemented onto the medium, *H. parviporum* cultures did not grow in any of the tested samples (Table 3). Against this background, the results obtained when the extract was supplemented into the medium were especially surprising. Relative to the control without acetone, mycelial growth dynamics showed both a reduction (max. c. −25 p.p.) and growth stimulation (max. c. +14 p.p.), with the highest dose of 4 mL causing the strongest inhibition. Relative to controls with equivalent acetone doses, stimulation was even stronger and more frequent, reaching a maximum level at the 1 mL extract dose (max. c. +39 p.p.). At the same time, inhibition was limited to a maximum reduction of c. −16 p.p. at the 4 mL dose (Table 4).

It is worth noting that, similarly to supplementation of *C. rangiferina* and *C. digitata* extracts into the medium, the growth dynamics of *H. parviporum* mycelium changed over time: stimulation occurred during the earlier phase of culture and, irrespective of dose, turned into inhibition towards the end (Table 4).

### 3.9. Potential of Extracts Obtained from Mixed Thalli of Three Lichen Species

In the acetone extract obtained from mixed thalli of *Cladonia arbuscula*, *C. rangiferina* and *C. digitata*, the concentration of polyphenols determined in two independent samples ranged from 46 to 62 $\mu g\cdot {\mathrm{mL}}^{-1}$. Interestingly, it was higher than the sum of polyphenol concentrations detected in extracts obtained from thalli of the individual species (Table 1), as also observed in another experiment [16]. This appears to be due to environmental conditions that determine variable metabolite production in the field, including soil properties [66] and habitat light and temperature [69], as already discussed for the extract from *Cetraria islandica*. Spatial variability in metabolite concentration, such as that reported for usnic acid, may also be relevant [70]. Consequently, metabolites present below the detection threshold in individual extracts may have reached detectable concentrations in the mixed extract.

Additional, unrecognised factors arising from interactions among lichen substances present in the thalli of different species during joint extraction may also influence the dissolution of lichen substances. This may reflect an as yet undetermined breakdown or transformation of acetone-dissolved compounds into other components, as suggested by the stability of atranorin in acetone and in fresh lichen thalli [23]. A similar phenomenon of higher polyphenol concentration during extraction from mixed thalli of several species was demonstrated by Furmanek et al. [16], although for a different combination of mixed thalli: *Cladonia arbuscula*, *C. rangiferina* and *Cetraria islandica*. Nevertheless, based on current knowledge, the influence of interactions among lichen substances present in thalli of different species on their enhanced or reduced solubility during extraction remains a working hypothesis.

The extract from mixed thalli of three lichen species proved to be a relatively rich matrix of compounds (Figure S6) compared with the other lichen extracts tested (Figures S1–S8). Chromatograms supported the putative identification of thamnolic and fumarprotocetraric acids (Figure S6B), as well as usnic acid and atranorin (Figure S6C), with the strongest signals assigned to usnic and thamnolic acids (Figure S6B,C). Usnic acid could originate mainly from *C. arbuscula* thalli, fumarprotocetraric acid and atranorin from *C. rangiferina*, and thamnolic acid from *C. digitata* thalli [63].

Interestingly, the compound matrix of the extract from mixed thalli of these three species (Figure S6C) was slightly poorer than that separated from *C. arbuscula* (Figure S1B) and markedly poorer than the matrix extracted from *C. rangiferina* thalli (Figure S2B). In the case of *C. digitata*, the compound matrix obtained from the mixture (Figure S6B) showed similarity in UV wavelength, with $\lambda_{\max}=219$ nm, to the extract from the single species *C. digitata*, with $\lambda_{\max}=220$ nm (Figure S3B). This is a value ($\lambda_{\max}=218;260;285;312$ nm) close to the data presented by [62] for thamnolic acid. The similar UV wavelength suggests that also in the extract from *C. digitata* there is a complex of substances including at least thamnolic acid and perhaps trace amounts of fumarprotocetraric acid, as reported for ethanol extract from *C. digitata* [13]. A general comparison of the compound complex containing thamnolic and fumarprotocetraric acids (Figure S6B) indicates a markedly poorer chemical composition than the qualitative profile of lichen substances contained in the extract from individual *C. digitata* thalli (Figure S3A–E; Table 2). This issue seems to suggest possible interactions among extracted lichen substances and can be treated as a premise in favour of the working hypothesis proposed above.

In cultures with the extract from mixed thalli of the three lichen species supplemented onto the medium, the reduction in *H. parviporum* mycelial growth dynamics ranged from c. 55 to 87 p.p. relative to the control without acetone for doses of 1 and 2 mL, respectively. The 3 mL extract dose completely inhibited measurable mycelial growth (Table 3). Relative to controls with an equivalent acetone dose, the reduction in mycelial growth dynamics was lower (c. 23 to 79 p.p.) (Table 3). This example indicates a substantial effect of extract dose on toxicity, with the effect becoming more “flattened” over time at the higher dose. It therefore appears that, in this case, the effects of blocking the synthesis of pathogen cell-building compounds by acetone-dissolved lichen substances were persistent and occurred even at low metabolite concentrations.

Cultures with the extract supplemented into the medium showed different growth dynamics. Relative to the control without acetone, increasing doses of 1–4 mL extract reduced *H. parviporum* mycelial growth dynamics by c. 26–51 p.p. This reduction was confirmed by comparison with controls containing an equivalent acetone dose, although the reduction was lower, in the range of c. 10–51 p.p., with the greatest reduction occurring with a supplementation of 3 mL of extract. The highest dose, 4 mL, was less toxic than the 2 and 3 mL doses (Table 4).

### 3.10. Potential of Extracts Obtained from Mixed Thalli of Five Lichen Species

The acetone extract obtained from mixed thalli of five lichen species, *Cladonia arbuscula*, *C. rangiferina*, *C. digitata*, *C. uncialis* and *Cetraria islandica*, showed the expected higher concentration of total polyphenols compared with the extract from mixed thalli of three epigeic species (Table 1). The concentration of determined polyphenols in this extract was also higher than the total concentration in all five extracts obtained from thalli of the individual lichen species (Table 1) [13]. This phenomenon supports the newly proposed hypothesis concerning possible interactions among substances present in thalli of different lichen species. This is also indicated by the qualitative profile of the extract, which was poorer in secondary metabolites reported by Smith et al. [63] than the chemical composition of the extract from mixed thalli of three epigeic lichen species (Table 2). Essentially, only two compounds were putatively identified: usnic acid and atranorin or protocetraric acid (Table 2). The signal putatively assigned to usnic acid could have originated mainly from thalli of *C. arbuscula* (Figure S1B) and *C. uncialis* (Figure S4B). Based on the retention times, Rt, of extracts from *C. rangiferina* and mixed thalli of the three epigeic species, it is more likely that the compound complex contained atranorin originating from *C. rangiferina* thalli than protocetraric acid (Table 2).

The composition of secondary metabolites in the extract from mixed thalli of five lichen species showed a poorer compound matrix than both the extract from mixed thalli of three lichen species (Figures S6 and S7) and the extracts obtained from individual *C. rangiferina* (Figure S2B) and *C. digitata* thalli (Figure S3A–E). However, the matrix was identical, apart from the peak attributed to the presumably present atranorin, to the matrix of compounds dissolved in the extract from *C. arbuscula* (Figures S1B and S7B), almost identical to the matrix from *C. uncialis* (Figures S4B and S7B), and different from that obtained for compounds from *Cetraria islandica* (Figure S5B). A dominant chromatographic signal was putatively assigned to usnic acid in the tested extract (Figure S7B).

*H. parviporum* mycelia did not grow in any sample in which the extract obtained from the five lichen species was supplemented onto the medium, indicating a 100 p.p. reduction in growth dynamics (Table 3). This contrasted with samples in which the extract was supplemented into the medium. In the latter case, only inhibition occurred, becoming stronger with increasing experiment duration and increasing concentration of both lichen substances and acetone itself acting on the pathogen (Table 3 and Table 4). Relative to the control without acetone, mycelial growth reduction increased with extract dose from 1 to 4 mL and was in the range of c. 24–57 p.p. Relative to controls containing an equivalent acetone dose, only the 1 mL extract dose inhibited *H. parviporum* growth more markedly, whereas the inhibitory effects of acetone alone and extracts at doses of 2, 3 and 4 mL were almost comparable. The reduction in mycelial growth dynamics was lower but comparable, reaching c. 7–51 p.p. (Table 4).

The inhibitory effect of the highest dose, 4 mL, of extracts obtained from mixed thalli of both three and five lichen species was essentially similar and slightly weaker than the potential of substances contained in 3 mL of extract (Table 4). This pattern suggests a similar inhibition mechanism resulting from a comparable set of active substances extracted from the two thallus mixtures, which contained overlapping species.

### 3.11. Potential of Extracts from Pseudevernia furfuracea

The concentration of total polyphenols in extracts from thalli of the epiphytic species *Pseudevernia furfuracea* was the highest among all tested extracts, including those obtained from mixed thalli of several species (Table 1). Similarly, Furmanek et al. [16] obtained a high concentration of metabolites from this species using a water–ethanol solvent. In contrast to the extraction result presented by Tekiela et al. [64], the chromatographic analysis indicated the putative presence of both atranorin and physodic acid (Table 2), as reported among the metabolites of *P. furfuracea* [63].

Based on m/z values, physodic acid can be assigned to several retention times, $R_{t}$ (Table 2; Figure S8B–G). However, based on the UV spectrum reported for atranorin, $\lambda_{\max}=212$, 263 and 314 nm [62], atranorin is most likely present at $R_{t}=6.636$ or $R_{t}=6.883$ (Figure S8B–C). The dominant chromatographic signal was putatively assigned to chloroatranorin, with m/z=407 and $R_{t}=7.905$ (Figure S8G), which had previously been shown in the chemical profile of *P. furfuracea* [62,71].

As observed for the extract from *Cetraria islandica* thalli and the extract from mixed thalli of three epigeic lichen species, the *P. furfuracea* extract supplemented onto the medium completely prevented the growth of *H. parviporum* mycelia (Table 3). In contrast, the extract from thalli of this epiphyte supplemented into the medium only strongly limited culture growth. Relative to the control without acetone, the reduction in pathogen mycelial growth dynamics was in the range of c. 48–100 p.p. Relative to the culture with an equivalent acetone dose, extract inhibition was slightly lower only at the 1 mL dose. In the remaining cases, the reduction in mycelial growth was similar relative to both controls, without and with acetone, and was in the range of c. 37–100 p.p. (Table 4).

Similarly to the test with the 3 mL dose of *C. digitata* extract supplemented onto the medium, the test with the 4 mL dose of *P. furfuracea* extract also showed delayed growth, which occurred only during the second half of the experiment (Table 4). This indicates a fungistatic effect caused by the complex of lichen substances present in this extract rather than a fungicidal effect. Likewise, the frequent absence of pathogen mycelial growth in tests with extract supplemented onto the medium cannot be interpreted as direct evidence of fungicidal potential (Table 3).

### 3.12. Effect of the Lichen-Substance Complex on the Growth Dynamics of Heterobasidion parviporum Mycelium

#### Effect of Extract-Supplementation Method

The measured differences in *H. parviporum* mycelial growth dynamics between the two supplementation methods (Table 3 and Table 4) have not been the subject of particular interest so far [25] and seem to result from at least several reasons.

In general, the stronger fungistatic potential of extracts supplemented onto the surface of the medium was caused by at least three factors: the type of solvent used, namely acetone; residual acetone on the medium surface after evaporation; and the concentration of acetone-dissolved lichen substances on the restricted surface of the agar medium.

The response of the tested *H. parviporum* mycelia to acetone, which mainly limited growth dynamics, was expected because fungi are not evolutionarily adapted to acetone exposure, although possible chemical hormesis was demonstrated in the control samples (Table S11) [25,64]. Moreover, the qualitative and quantitative profile of acetone extracts appears to differ from that of water extracts, as indicated by differences in total polyphenol concentrations between acetone and water extracts [16].

Acetone residues remaining after evaporation from the medium, which could penetrate deeper outer layers of Hansen’s medium, could limit mycelial growth dynamics, as evidenced by comparing samples with an extract supplemented into the medium. In addition, local residual acetone on the medium surface could have contributed to mycelial inhibition, which was observed as delayed mycelial growth in the case of extracts from *Cladonia digitata* (Table 3) and *Pseudevernia furfuracea* (Table 4). Sterilisation of the medium with the acetone extract mixed into it seems to have substantially promoted acetone evaporation and dispersed residual acetone throughout the medium. Thus, locally higher acetone concentrations were neutralised and their effect on mycelial growth dynamics was reduced, perhaps markedly (Tables S22B, Table 3 and Table 4).

Similarly, the concentration of lichen substances on the external surface of the medium, together with acetone residue, contributed to stronger fungistatic potential. It is unknown to what depth dissolved lichen substances could penetrate the outer part of the solidified medium [25]; however, compared with supplementation into the medium, this does not seem to be decisive. Moreover, comparison of mycelial dynamics between the two extract-supplementation methods does not confirm previously suggested fungicidal effects against *H. parviporum* (Table 3 and Table 4). Even if such effects occurred, the present data do not provide direct evidence of fungicidal potential. Only transplantation of mycelial inocula onto new, unsupplemented agar medium could demonstrate whether the tested cultures retained viability.

The measured profungal effects, mainly in the method in which extracts were mixed with the medium and therefore exerted a systemic effect, may appear unexpected [64], but they also indirectly support the hypothesis that stimulation of macrofungal mycelium may occur more frequently under natural conditions, especially when rainwater acts as the solvent [16,25]. Such stimulation may result from the reduced influence of acetone and from lower local concentrations of lichen substances caused by their dispersion in the agar medium, which may weaken inhibitory allelopathic effects [25].

The potential of the extracts may also have been influenced by the temperature used during sterilisation of the medium containing mixed extracts, owing to the chemical stability of lichen substances. For lichen-substance complexes, this issue remains unresolved [16,25], but the disintegration of the chemical structure of isolated usnic acid has been observed [72]. In addition, atranorin isolated directly from lichen thalli may have a more stable chemical structure than a previously isolated and redissolved metabolite [23]. This indirectly indicates greater stability of lichen substances in the extract as a substance complex, at least for the signal putatively assigned to atranorin (Table 4). It remains unclear how and to what extent sterilisation temperature determined the potential of lichen extracts. Nevertheless, the measured mycelial growth dynamics occurred across a wide range (Table 4), probably influenced by interactions among substances [16,25]. The experiment therefore indirectly suggests, but does not chemically confirm, the relative thermal stability of compounds responsible for fungistatic effects within the lichen-substance complex [16,23].

### 3.13. Mycelial Growth Dynamics in Cultures with Extracts from Different Lichen Species

#### 3.13.1. Extract Supplementation onto the Medium

The obtained results indicate interspecific differences among the extracted lichen-substance complexes. Because the tested fungus, *H. parviporum*, acts as a parasite [46], fungistatic effects on mycelial growth dynamics are the expected and desirable outcome from an applied perspective.

Despite the absence of mycelial growth in samples treated with extracts from *Cetraria islandica*, mixed thalli of five epigeic lichen species and *P. furfuracea*, significant statistical correlations were demonstrated among the remaining extracts tested (Table S20).

Interspecific relationships among the lichens from which the extracts were obtained were demonstrated only for samples treated with 1 mL of extract. Among these samples, the strongest and statistically significant inhibition was measured for the *Cladonia uncialis* extract. Conversely, extracts from *C. rangiferina* and *C. digitata* showed the weakest inhibitory potential (here: stimulation of mycelial growth). Statistical analysis did not reveal any significant differences in mycelial growth dynamics between samples treated with the *C. arbuscula* extract and those treated with the extract from mixed thalli of three epigeic lichen species, both of which showed relatively strong inhibition of mycelial growth dynamics.

At extract doses of 2 and 3 mL, mycelial inhibition was sufficiently strong that interspecific effects of the lichen-substance complexes became undetectable (Table S20).

##### Mycelial Response to Dose Volume

Mycelial dynamics in test samples compared with control samples containing equivalent acetone doses showed several dose-response patterns (Table 3). Based on the results obtained, the following response types can be distinguished: complete absence of mycelial growth, interpreted as putative complete inhibition, as observed for extracts from *Cetraria islandica*, mixed thalli of five lichen species and *P. furfuracea*; increased mycelial inhibition at higher extract dose, as observed for extracts from mixed thalli of three lichen species, *Cladonia uncialis*, *C. rangiferina* and *C. digitata*; and stimulation of mycelial growth followed by inhibition at higher dose volumes, as observed for the *C. digitata* extract.

These responses are consistent with conclusions from a critical analysis of previous studies on mycelial responses to lichen substances [25]. Notably, the use of extracts obtained with identical methodological rigour allows the observation of different dose-effect relationships for a single fungal species (Table 3), indicating different application possibilities, including stimulatory effects [27,38].

In the context of the expected inhibition of *H. parviporum* mycelial growth dynamics, the most advantageous lichen-substance complexes are those that completely reduced measurable mycelial growth throughout the experimental period. Extracts showing a relatively strong, although not necessarily statistically significant, reduction in mycelial growth dynamics also meet the expected criteria, including the use of a larger dosage (Table 3 and Table S20).

The effect of initial inhibition of mycelial growth dynamics followed by stimulation at the final stage of the experiment, as observed for the 1 mL dose of *C. rangiferina* extract, indicates continuous metabolism of dissolved lichen substances by *H. parviporum* mycelium (Table 3). Progressive utilisation of lichen substances seems to influence interactions within the lichen-substance complex [16,25], thereby changing its biological potential. Dissolved lichen substances may be used as a carbon source, as emphasised by Stark and Hyvärinen [73], and perhaps also as a nitrogen source [74].

In addition, the tested fungal species belongs to Basidiomycota, a group with substantial potential to decompose more complex chemical compounds [25]. This may facilitate access to nutrient resources in the form of lichen metabolites.

#### 3.13.2. Extract Supplementation into the Liquid Medium

Comparison with control samples containing equivalent acetone doses showed that the extract from the epiphytic species *P. furfuracea* had the highest inhibitory potential against *H. parviporum* mycelial growth dynamics. A relatively strong and statistically significant inhibitory effect was also demonstrated for the extract from *C. arbuscula*. In contrast, the inhibitory potential of extracts from *C. uncialis* and from mixed thalli of three and five epigeic lichen species was only partly significant or statistically insignificant (Table S21). Indeed, the weakest inhibition was observed for extracts from *Cladonia rangiferina* and *Cetraria islandica* (Table S21).

Compared with the dose-effect relationship observed in samples with extracts supplemented onto the medium, the results for extracts supplemented into the medium indicated additional effects. First, lower extract doses stimulated mycelial growth, followed by inhibition at intermediate doses and, in some cases, again a possible stimulation effect at the highest tested dose; this pattern was observed for extracts from *C. rangiferina*, *C. digitata* and partly *Cetraria islandica* (Table 4). Second, some extracts showed increasing inhibition at lower dose volumes, followed by weaker inhibition at the highest tested dose; this pattern was observed for extracts from *C. arbuscula* and mixed thalli of three and five lichen species (Table 4). These effects are consistent with results reported by other researchers, especially the phenomenon of low-dose stimulation [25].

The weaker inhibition of mycelial growth dynamics, including stimulation, seems to be determined by interactions among lichen substances [16,25] and/or possible variability in nutrient uptake by mycelial hyphae [75,76]. However, the regular pattern of reduced mycelial growth potential at the highest tested dose volumes suggests a specific biochemical effect. One possible explanation is chemical hormesis, which often has a U-shaped form [77,78,79]. Assuming that U-shaped chemical hormesis is common, it can also be expected that the hormetic effect may be observed in other tested extracts when different dose volumes and extract strengths are used, thus producing different biological effects [80,81]. Interestingly, the most common magnitude of the hormetic response is approximately 50% [81], which is consistent with the present results, where the maximum stimulation of mycelial growth dynamics was c. +48 p.p. relative to the control sample, as observed for the 1 mL dose of *C. digitata* extract supplemented onto the medium (Table 3) [25].

From the perspective of interactions among lichen compounds and their metabolism by the tested fungal species, interpretation of the effects is analogous to that presented above—for extracts supplemented onto the medium. However, stimulation of mycelial growth during the initial stage of culture, followed by a lower profungal effect at the end of the experiment, as observed for the 1 mL dose of extracts from *Cladonia rangiferina*, *C. digitata* and *Cetraria islandica*, may indicate that the mycelium initially metabolises lichen substances as readily available carbon sources, such as primary metabolites, sugars and polyols. Less readily available nutrient sources, including secondary metabolites generally associated with antifungal potential, may then be used later [25,28]. This highlights the need to interpret biological effects at the level of the whole lichen-substance complex [13,25].

It can be assumed that low concentrations of secondary metabolites may function as signalling molecules that transiently enhance metabolic activity and hyphal extension rates, although this requires experimental confirmation. The non-linear growth responses detected across the dose gradient are therefore consistent with a shift from stimulatory to inhibitory modes of action as the balance between nutrient and stress-inducing effects changes.

### 3.14. Specific Issues Concerning the Potential of Lichen Extracts

#### 3.14.1. Effect of Trace Amounts of Lichen Substances on Mycelial Growth Dynamics and the Potential Inhibition Mechanism

The negative spectrophotometric result and trace concentrations of total polyphenols in extracts, mainly from *Cetraria islandica*, *Cladonia rangiferina* and *C. uncialis* (Table 1), together with their effects on mycelial growth dynamics, demonstrate that even trace amounts of lichen substances, including secondary metabolites (Table 2), can exert clear and statistically significant profungal or fungistatic effects. Profungal effects were observed for *C. rangiferina* and *Cetraria islandica* extracts, whereas fungistatic effects were observed for *C. uncialis* and *Cetraria islandica* extracts (Tables S20 and S21).

The wide variability in mycelial growth dynamics observed in samples with *C. islandica* extract may indicate potential difficulties in the practical application of this lichen-substance complex. Such difficulties may be determined by the previously indicated dependence on the dispersion of secondary metabolites in the substrate [20,82,83].

Moreover, it can be suspected that protolichesterinic acid, as a representative of the fatty-acid class (Table 2), may induce a fungistatic effect through a mechanism related to disruption of the lipophilic part of the fungal cell membrane, as suggested for dermatophytes [84]. It is possible that the observed fungistatic effects result from inhibition of fungal DNA replication, as indicated by studies on anti-proliferative activity [85]. In the case of usnic acid present in the *C. uncialis* extract (Table 2), a possible mechanism is uncoupling of oxidative phosphorylation in mitochondria [86] or inhibition of DNA and RNA synthesis, as demonstrated in bacteria [87]. In general, lichen secondary metabolites, including the putatively identified atranorin, fumarprotocetraric acid and physodic acid (Table 2), may act through oxidation-related mechanisms [88]. However, the mechanisms underlying the inhibitory and stimulatory potential of lichen-substance complexes, including secondary metabolites, on mycelial growth dynamics in macrofungi remain insufficiently recognised.

The more frequent putative identification of usnic acid and atranorin results not only from their variable concentrations in lichen thalli [89,90], but also from the accumulation of these compounds in the outer layer of the lichen thallus, the cortex, which may facilitate solvent accessibility; this has been shown for usnic acid [73]. Their solubility is also determined by the affinity of acetone, the simplest ketone with a carbonyl group [91], for the structure of polyketide compounds [92].

#### 3.14.2. Application Aspects of Acetone Extracts

The direct application of acetone-extracted lichen-substance complexes as plant-protection agents is not permitted because acetone can adversely affect soil microbial biota, including the tested fungal species (Table 3). Neutralisation of the extraction solvent (Table 4) and dissolution of the dry extract in water could enable the application of lichen-substance complexes. However, changing the solvent introduces additional issues, including changes in substance solubility and interactions among substances [25,28]. These factors require testing both in the laboratory and under field conditions. Therefore, the response of mycelium to dry extract residues redissolved in water remains unknown and requires further research. A more practical approach may be to dilute acetone extracts with water.

For application purposes, the effect of chemical hormesis should also be considered, because a U-shaped response was observed in the analysed mycelial growth dynamics (Table 4) [78]. Determining the position of the dose-response (effect) relationship should allow more precise determination (optimisation of the dose and rigour of the extraction process) of extract dose volume and extract strength to induce the expected effect on mycelial growth dynamics.

In the context of limiting *H. parviporum* mycelial growth, without necessarily producing fungicidal effects, the delayed onset of growth observed in tests with extracts from *C. digitata* thalli (Table 3) and *P. furfuracea* thalli (Table 4) is particularly interesting. Assuming interspecific effects among fungal mycelia, a temporal shift in the growth of the tested pathogen could potentially allow the development of more desirable fungal species, such as ectomycorrhizal or antagonistic species. The potential of lichen complexes obtained with acetone should therefore be examined in more detailed studies in the laboratory and forest nursery. Nevertheless, the present results highlight the possible practical relevance of mycelial inhibition in forest nurseries.

In an ecological context, the profungal potential of acetone-extracted lichen complexes [64] supports the hypothesis that lichen-substance complexes dissolved in rainwater may more commonly produce profungal effects [16,25,82], although studies by Prokopiev et al. [83] indicate more complex relationships, including effects on ectomycorrhizal fungi. Irrespective of the effects of the lichen-substance complex on *H. parviporum* mycelial growth dynamics, the biosynthesis of high-energy secondary metabolites can be advantageous for the tested lichen species in the context of optimal defence theory [8,93].

From an ecological perspective, contact between lichen metabolites and *H. parviporum* mycelium is not purely theoretical. In managed and semi-natural spruce forests, lichen thalli and fragments frequently occur on deadwood surfaces, stumps and coarse woody debris, which can also serve as infection courts and colonisation substrates for *H. parviporum*. Mechanical disturbance, wet–dry cycles and decomposition can release lichen secondary metabolites, including water-extractable substances [16], into microhabitats where *H. parviporum* initiates or maintains colonisation. The in vitro assays therefore mimic a realistic scenario in which lichen-derived compounds form part of the local chemical environment that may either suppress or, under some conditions, facilitate pathogen growth.

The antifungal profiles observed for several lichen extracts highlight their potential as sources of lead compounds for novel, more ecosystem-compatible strategies to manage *Heterobasidion* root rot. In principle, such metabolites or enriched fractions could be developed into protective stump treatments [53] or surface coatings that complement existing chemical controls and genetic resistance breeding in Norway spruce, *Picea abies*. However, any practical application will require careful evaluation of phytotoxicity to host tissues, stability under field conditions, non-target effects on beneficial fungi, and cost-effective extraction or synthesis. The present results should therefore be viewed as an initial screening step that prioritises lichen species and extract types for more integrative in planta and field-based experiments.

#### 3.14.3. Bioprospecting Potential of Lichen-Substance Complexes

Given the strongest levels of mycelial inhibition demonstrated by the tested extracts (Table 3 and Table 4) and the putatively identified secondary metabolites (Table 2), several cautious conclusions can be drawn regarding their bioprospecting potential. Because the metabolite assignments were not confirmed by MS/MS or authentic standards, the following associations remain tentative.

The similar potential of extracts from *C. arbuscula*, *C. uncialis* and mixed thalli of five epigeic lichen species (Table 4) may have been associated with usnic acid content (Figures S1, S4 and S7). This may be related to the ability of acetone to dissolve metabolites accumulated in the extracellular layer of the lichen thallus [73].

The potential of the extract from mixed thalli of three epigeic species may have been associated with usnic acid, together with putatively identified thamnolic acid (Figure S6). The comparable inhibition of mycelial growth by extracts from *C. arbuscula*, *C. uncialis* and mixed thalli of three lichen species is consistent with a possible contribution of usnic acid to the observed inhibition (Table 4).

The fungistatic potential of the *P. furfuracea* extract may have been associated with physodic acid, chloroatranorin and atranorin (Figure S8). Because of the complex compound matrix of this extract, more definitive conclusions are not justified.

Overall, the experiment was based on the cultivation of *H. parviporum* mycelium in 270 individual Petri dishes of test samples. In this context, the use of five biological replicates per treatment supports within-experiment repeatability and facilitates reproduction of the experimental protocol. Independent repetition of the complete experiment would provide further confirmation of reproducibility.

### 3.15. Limitations and Future Prospects

This study has several limitations that indicate clear directions for future work. First, all assays were conducted under controlled in vitro conditions, which do not fully represent the physical and biological complexity of root and stump environments. Second, potential negative effects of lichen extracts on spruce tissues or on non-target members of the wood-inhabiting mycobiome were not assessed. Third, the study focused on crude acetone extracts, whereas the active compounds and possible synergistic interactions within each extract remain to be clarified. Addressing these aspects via in planta experiments and chemically fractionated bioassays will be essential for translating the promising inhibitory effects reported here into ecologically robust and operationally feasible control measures.

Moreover, the stability of the extracted lichen-substance complexes under UV exposure, changing moisture conditions, and different medium or soil pH values was not assessed and requires further study.

As discussed, usnic acid is one of the most promising secondary metabolites for further investigation against *H. parviporum* mycelial growth. To our knowledge, the potential of isolated usnic acid and other individual lichen secondary metabolites against *H. parviporum* sensu stricto has not yet been investigated and should be further explored [25].

More specific chemical analysis of the acetone extracts, including MS/MS spectrometry and comparison with authentic standards, is required to confirm the putatively identified lichen secondary metabolites. The compounds reported in the present article should therefore be regarded as putatively identified, including usnic acid despite its consistent chromatographic signal.

## 4. Materials and Methods

### 4.1. Tested Fungal Species

Pure cultures of *Heterobasidion parviporum* used in the experiments were obtained from the phytopathological laboratory of the Department of Forest Ecosystem Protection, University of Agriculture in Krakow, courtesy of Dr C. Bartnik.

**Figure 1:**
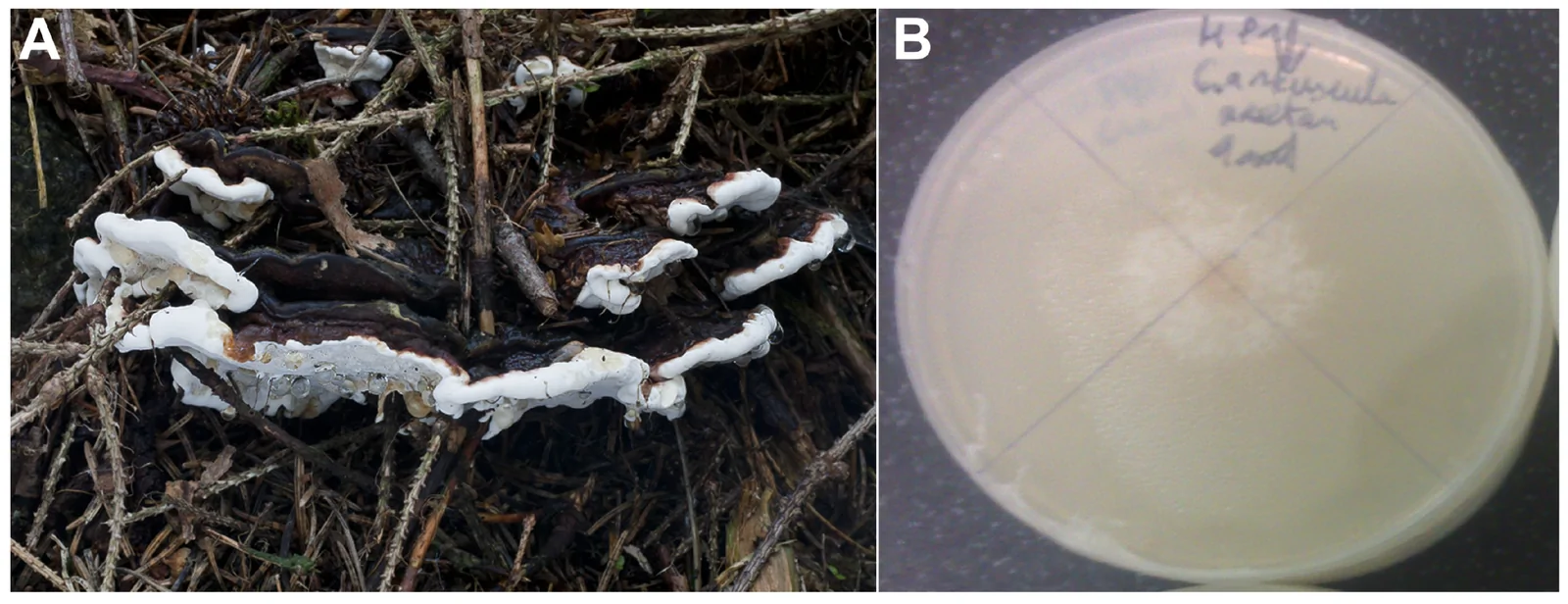
*Heterobasidion parviporum* Niemelä & Korhonen. (**A**) Sporocarp; photograph by Lucie Zíbarová, source: https://www.mykologie.net/index.php/houby/podle-morfologie/chorose/item/685:heterobasidion-parviporum (accessed on 2 July 2026). (**B**) Mycelium cultured on Hansen’s medium under in vitro conditions; photograph by Łukasz Furmanek.

The pathogen *Heterobasidion parviporum* Niemelä & Korhonen, identified and described by [94], develops on roots and at the bases of trees, singly or in groups, forming perennial, corticioid to weakly bracket-shaped fruiting bodies (Figure 1). The margin of the fruiting body is white to yellowish, uneven and wavy; the upper surface is reddish-brown to dark reddish-brown and blackish-brown in the central part. The surface is matt, often wrinkled and bumpy. The tubular hymenophore is white to cream, with pores up to 0.2 mm in diameter, narrower than in other species of this genus. Basidiospores are white to pale cream.

The mycelium of *H. parviporum* in in vitro culture on Hansen’s medium has a delicate, whitish, loose and semi-transparent filamentous structure in the younger part. In older parts of the colony, it becomes cream-coloured and more compact over time (Figure 1B).

### 4.2. Extracted Lichen Species

The experiment used five large-thallus terricolous lichen species, namely *Cladonia arbuscula* (Wallr.) Flot., *C. rangiferina* (L.) Weber, *C. digitata* (L.) Baumg., *C. uncialis* (L.) F.H. Wigg. and *Cetraria islandica* (L.) Ach., and one large-thallus epiphytic species, *Pseudevernia furfuracea* (L.) Zopf. These species were selected because they are frequent in pine and spruce forests widespread in central and northern Europe, which also provide habitats for *H. parviporum*, and because they can be readily identified using morphological and chemical characters described in taxonomic keys [63]. The results on the solubility of total lichen polyphenols, especially for *P. furfuracea*, were also important [16]; in the event of promising results, additional lichen samples could be collected rapidly for further extraction.

Specimens of the tested lichen species came from the Janów Forests in eastern Poland and the Gorce Mountains in the Western Carpathians, southern Poland. These collections were also used in studies on the solubility of lichen metabolites in rainwater, which form part of a larger research project on the effects of water extracts on macromycetes [16], and on the cytotoxic and antioxidant potential of lichen substances against human cancer cells. Detailed localities and herbarium vouchers for the lichen species used, except *Cetraria islandica*, are provided in [13,14]. *Cetraria islandica* used in the present experiment was collected separately in a pine forest on the Równina Biłgorajska Plain, ca. 0.5 km S of the town of Zaklików, eastern Poland, 50°44′55″ N, 22°06′05″ E, 185 m a.s.l., 19 June 2018, leg. P. Czarnota, comparative voucher no. Ex GPN 8641 [14]. Whole lichen thalli were divided into smaller fragments in the laboratory during dry cleaning to remove organic and mineral substances but they were not ground before extraction. Portions of the collections used in the study are stored as duplicates in the Herbarium of Gorce National Park (GPN; curator P. Czarnota) in Poręba Wielka, Poland.

### 4.3. Preliminary Experiment

#### Determination of Growth Medium and Culture Temperature

In the first stage of the experiment, culture tests were performed in Petri dishes to optimise the in vitro growth conditions of *H. parviporum* mycelium with respect to culture medium and temperature. The basic criterion for optimisation was the mycelial growth rate, measured as the time required to overgrow the entire surface of the tested medium. Mycelial cultures were inoculated on three pre-selected agar media: PDA, modified PDA medium supplemented with maltose, and Hansen’s medium (recipe in Table S1). Mycelial growth on each medium was observed at 20, 22, 24, 26 and 28 °C. This temperature range was adopted on the basis of literature data [95].

On this basis, cultures on Hansen’s medium at 28 °C were selected for the main experiment on the potential activity of lichen substances against *H. parviporum* mycelium. These conditions were also determined to be optimal for comparative experiments on the responses of other macromycetes to lichen extracts, which constituted subsequent stages of the research conducted by the team.

### 4.4. Determination of Extract Doses

#### 4.4.1. Supplementation onto the Surface of a Solidified Medium

First, an experiment was conducted to determine the optimal time interval between applying acetone solvent or acetone extracts onto solidified Hansen’s medium, i.e., the time required for acetone evaporation, and inoculation with *H. parviporum* mycelium. This technical and methodological step was performed to minimise the potential negative effect of acetone itself on the culture.

Approximately 20 mL of Hansen’s medium (recipe in Table S1) was poured into each Petri dish. After solidification, acetone was supplemented onto the medium surface at volumes of 1, 2, 3, 4 and 5 mL. The dishes were covered with lids, without parafilm protection, and left in a laminar-flow cabinet with the airflow switched on at ca. 21 °C. Visual observations of acetone evaporation were carried out at hourly intervals. Because complete evaporation of 5 mL of acetone only took place after nearly 24 h, and *H. parviporum* cultures in control samples supplemented with 4 and 5 mL of acetone showed no growth during this period, subsequent experiments were performed with extract doses of 1, 2 and 3 mL. Exceptionally, because of slow mycelial growth in control tests with 3 mL of acetone, only doses of 1 and 2 mL were used in experiments with extracts of *Cladonia arbuscula* and *C. rangiferina*.

#### 4.4.2. Supplementation into a Non-Solidified Medium

For tests in which analogous volumes of acetone or extracts were supplemented into the medium before solidification, evaporation tests were not performed, as they were considered irrelevant. To maintain the most comparable experimental conditions, including drying of the control sample without extract or solvent, fungal cultures were inoculated onto autoclaved media containing 1, 2, 3 and 4 mL of extract mixed in before solidification after 22 h. This interval corresponded to the time required for evaporation of 4 mL of acetone supplemented onto the solidified medium. For technical reasons, extract supplementation at a dose of 5 mL was omitted.

### 4.5. Preparation of Acetone Extracts

The acetone extracts were prepared according to the methodology for acetone extraction of lichen thalli described previously by our team [16]. The suggested extraction approaches and the methodology for measuring the growth dynamics of mycelial cultures of macrofungal species described by [25] were also taken into account.

The tested extracts were prepared separately from purified thalli of six individual lichen species, namely *Cetraria islandica*, *Cladonia arbuscula*, *C. rangiferina*, *C. digitata*, *C. uncialis* and *Pseudevernia furfuracea*, and from mixed thalli combining three species (*C. arbuscula* + *C. rangiferina* + *C. digitata*) or five species (*C. arbuscula* + *C. rangiferina* + *C. digitata* + *C. uncialis* + *Cetraria islandica*).

Thalli of each lichen species were extracted at a ratio of 1 g per 100 mL acetone, or at corresponding proportions, in glass beakers for 10 min at room temperature. The extracts obtained were poured through gauze to separate the extracted lichen thalli. To obtain extracts from mixed thalli of several lichen species, the following proportions were used: 1 g of thallus of each species per 100 mL acetone, e.g., 3 g per 100 mL for extracts from three combined species or 5 g per 100 mL for extracts from five combined species, or corresponding proportions thereof.

For supplementation onto a solid medium, the previously determined doses of 1, 2 and 3 mL were dispensed using an automatic pipette. The dishes were then left to allow acetone and extracts to evaporate from the culture medium. Extracts supplemented onto the surface of the culture medium were not sterilised in an autoclave.

For supplementation into the liquid medium before pouring into Petri dishes, appropriate volumes of acetone for the control sample and acetone extract from individual species for the test sample were prepared. These were then mixed with Hansen’s medium in flasks in proportions giving the tested doses of 1, 2, 3 and 4 mL per Petri dish, while maintaining the medium volume at approximately 20 mL. The medium prepared in this way was sterilised in an autoclave.

### 4.6. Spectrophotometric Analysis of Extracts

Spectrophotometric analysis was performed to determine the total polyphenol content using the Folin–Ciocalteu method (Table 1). The analytical method and measuring apparatus were identical to those described in [16]. The concentration of phenolic compounds extracted with acetone was determined before extract supplementation using Folin–Ciocalteu reagent (POCH, Gliwice, Poland). A UV–Vis Evolution 300 spectrophotometer (Thermo) was used, and the data were interpreted using VISION software, version 4.20. Each of the three biological samples (N = 3) was read three times in the spectrophotometer.

Since it was impossible to inoculate *H. parviporum* cultures simultaneously in both supplementation variants, owing to the time required for acetone applied onto the medium to evaporate, extraction of lichen substances was carried out independently for these two variants. Consequently, extracts from thalli of the same species differed in polyphenol concentration and therefore also in the concentration of biologically active substances (Table 1).

### 4.7. Chromatography of Acetone Extracts

The qualitative profile of acetone extracts prepared for supplementation was determined by ultra-performance liquid chromatography with photodiode-array and electrospray-ionisation mass-spectrometric detection (UPLC–PDA–ESI–MS) only for extracts supplemented into the liquid medium. It was assumed that extracts used in experiments with supplementation onto solid media, obtained from the same lichen collections, would not differ qualitatively. The quantitative profile of the extracts was not determined.

Because acetone could evaporate from the flask during sterilisation of extracts in an autoclave, extract samples taken for chromatographic separation were not sterilised. Consequently, the chromatographic analysis described the extracts before autoclaving, and their chemical composition after sterilisation was not assessed.

Analyses were performed using a Waters ACQUITY chromatograph (Micromass, Manchester, UK) coupled with a photodiode-array detector and a tandem quadrupole mass detector (TQD). Separation was performed on a C_18_ BEH column, 100 mm × 2.1 mm, with a particle size of 1.7 μm (Waters). A 0.1% aqueous solution of formic acid (eluent A) and acetonitrile (eluent B) were used as eluents, with the following gradient: 20% B and 80% A to 100% B and 0% A over 8 min. Separations were performed at a mobile-phase flow rate of 0.35 mL min^−1^ and a column temperature of 50 °C. The analysis time was 9.5 min, and the injection volume was 5 μL. The mass-detector operating parameters were as follows: capillary voltage, 3.5 kV; sampling cone voltage, 45 V. The ion-source and desolvation temperatures were 120 °C and 350 °C, respectively. Nitrogen at a flow rate of 800 L h^−1^ was used as the carrier gas. Detection in negative-ion mode was performed in the *m*/*z* range of 120–1200. Data collection and analyses were performed using MassLynx 4.1 software (Waters).

Samples of acetone extracts from lichen thalli were prepared for chromatographic analysis as follows:

1. To retain those lichen substances present in the acetone extract on the solid-phase extraction filter, 150 mL of distilled water was added to 50 mL of extract.
2. The resulting 200 mL of diluted acetone lichen extract was filtered through a 0.45 μm MCE syringe filter, 33 mm in diameter.
3. Solid-phase extraction of the diluted extracts was performed using Oasis Prime HLB Plus Light filters. A volume of 200 mL of diluted acetone extract was used, except for the extract obtained from *Pseudevernia furfuracea*, for which 145 mL of the 200 mL diluted extract was filtered. Lichen substances retained on the filter were eluted with 5 mL of methanol.
4. A 0.2 mL aliquot of lichen extract was transferred to a vial for chromatographic analysis, and 0.8 mL of distilled water was added to obtain a final volume of 1 mL.

Putative identification of lichen secondary metabolites was based on mass-to-charge ratio (*m*/*z*) values, retention times (Rt), and UV spectra compared with data from [62] (Table 2). MS/MS fragmentation was not performed. No analysis of potential primary metabolites of lichens was performed.

### 4.8. Mycelium Culture Conditions

The medium was sterilised in flasks in an autoclave at 121 °C for 20 min. It was poured into Petri dishes using a FlexiPump peristaltic pump (Interscience) at a volume of approximately 20 mL per dish. In tests with extracts supplemented onto the solidified surface of the medium, the extracts were applied using an automatic pipette. The medium with the extract prepared in this way was stored in closed Petri dishes in a laminar-flow cabinet with the UV lamp switched on to reduce the likelihood of contamination.

After 22 h, *H. parviporum* mycelial inocula approximately 5 mm in diameter were placed in the centre of each dish in five replicates for each tested extract dose (N = 5). After inoculation, the dishes were sealed with parafilm and placed in a thermostatic cabinet maintained at a constant temperature of 28 °C.

Mycelial growth was determined every 12 h by measuring two perpendicular diameters of the colony (Figure 1B) to an accuracy of 1 mm. Measurements were terminated when the entire surface of the medium had been overgrown in at least one of the five biological replicates representing a given extract dose or, in the case of slow growth, after a predefined time set for the control test. The poisoned food technique (PFT) was used, following methodological suggestions in [25]. Culture time was defined as the last measurement hour before the medium was completely overgrown.

The control tests consisted of: (1) cultures of *H. parviporum* mycelium on the same Hansen’s medium without supplemented solvent, in five replicates (N = 5); and (2) cultures of the pathogen with acetone supplemented onto the surface of the solidified medium or into the liquid medium before solidification, at doses analogous to those used in the test samples, in five replicates for each dose (N = 5).

### 4.9. Statistics

At each measurement time, differences in the dimensions of *H. parviporum* mycelium among supplementation variants, doses and extracts obtained from particular lichen species were analysed using the non-parametric Kruskal–Wallis test for multiple independent samples or groups (Tables S2–S21) or the Mann–Whitney U test for two independent samples or groups (Table S22). Analyses were performed in STATISTICA, version 13.1.

## 5. Conclusions

Based on the presented analysis of the biological potential of acetone-extracted lichen-substance complexes on the growth dynamics of *H. parviporum* mycelium, the following conclusions can be drawn:

1. Acetone-extracted lichen complexes exerted both antifungal and profungal effects.
2. The biological potential exerted on the tested fungus was broad and included complete inhibition of mycelial growth dynamics, fungistatic effects and stimulatory effects. The most frequently observed response was fungistatic, whereas stimulation of mycelial growth dynamics reached a value of c. +50 p.p.
3. Complete inhibition of mycelial growth dynamics cannot be interpreted as a fungicidal effect on the basis of the present results.
4. Acetone exerted a strong fungistatic effect and should be minimised and controlled through the adopted research methodology in order to obtain the most accurate results.
5. The biological potential of lichen-substance complexes was strongly dependent on the dispersion of lichen substances, indicating differences between local and systemic effects.
6. The observed effects on mycelial growth dynamics demonstrated a U-shaped chemical hormesis pattern.
7. Analysis of the qualitative profile of secondary metabolites in extracts obtained from mixed thalli of three and five epigeic species allows one to formulate a hypothesis concerning unrecognised interactions among metabolites present in mixed thalli of different lichen species and their altered solubility from lichen thalli. However, the small number of biological replicates of test extracts does not allow broader conclusions to be drawn.
8. The extracted lichen-substance complex appears to retain biological activity after exposure to high temperature, although the chemical stability of individual secondary compounds was not directly assessed.
9. The most effective extracts limiting the growth of *H. parviporum* mycelium were those obtained from *C. arbuscula*, *C. uncialis* and *P. furfuracea*.
10. Mycelial growth stimulation was achieved using lichen-substance complexes mainly from thalli of *Cladonia digitata*, *C. rangiferina* and *Cetraria islandica*.
11. The qualitative profile of acetone extracts showed that, among secondary metabolites, inhibitory effects on mycelial growth dynamics were primarily associated with extracts in which usnic acid or a complex comprising physodic acid, chloroatranorin and atranorin was putatively identified.
12. The results indirectly support the hypothesis that lichen-substance complexes may exert more frequent stimulatory effects on macromycetes under natural environmental conditions [25].
13. The delayed onset of mycelial growth indicates the need to cultivate fungi for as long as possible, preferably until the mycelium reaches the Petri-dish wall [25].
14. For application purposes, more detailed experiments should be conducted using acetone extracts, e.g., by dilution with water.

## Figures and Tables

**Table 1:** Concentrations of polyphenols in acetone extracts used in experiments on the potential activity of lichen substances against *Heterobasidion parviporum* mycelium.

Lichen Extract	Phenol Concentration (μg mL^−1^), Mean ± SD
Experiment with extract supplementation onto the medium after solidification	
*Cetraria islandica*	Negative result
*Cladonia arbuscula*	4.68 ± 0.71
*Cladonia digitata*	12.04 ± 0.47
*Cladonia rangiferina*	3.60 ± 0.95
*Cladonia uncialis*	0.04 ± 0.02
*Cladonia arbuscula* + *C. rangiferina* + *C. digitata*	43.60 ± 0.85
*Cladonia arbuscula* + *C. rangiferina* + *C. digitata* + *C. uncialis* + *Cetraria islandica*	62.84 ± 0.50
*Pseudevernia furfuracea*	199.76 ± 0.63
Experiment with extract supplementation into the medium before solidification	
*Cetraria islandica*	Negative result
*Cladonia arbuscula*	15.00 ± 1.50
*Cladonia digitata*	32.12 ± 0.86
*Cladonia rangiferina*	0.80 ± 0.34
*Cladonia uncialis*	Negative result
*Cladonia arbuscula* + *C. rangiferina* + *C. digitata*	62.50 ± 1.15
*Cladonia arbuscula* + *C. rangiferina* + *C. digitata* + *C. uncialis* + *Cetraria islandica*	74.40 ± 1.40
*Pseudevernia furfuracea*	325.80 ± 0.25

**Table 2:** Chemical composition of lichens known from the literature and secondary metabolites putatively identified by UPLC–PDA–ESI–MS in acetone extracts supplemented into Hansen’s medium. Molecular weights of secondary metabolites are given according to [62]. Chromatograms for extracts obtained from the tested lichen species and their mixtures are presented in the Supplementary Materials (Figures S1–S8).

Lichen Extract	Chemical Composition Based on Literature Data [63]	Chemical Composition Based on UPLC–PDA–ESI–MS			
		Chemical Class of the Putatively Identified Metabolite [5]	*R_t_* (min)	*m*/*z*	Putatively Identified Secondary Metabolite
*Cladonia arbuscula*	Psoromic and usnic acids	Usnic acid derivatives	7.599	343	Usnic acid
*Cladonia rangiferina*	Fumarprotocetraric acid; atranorin	β-orcinol depside	7.599	373	Atranorin
*Cladonia digitata*	Thamnolic acid	—	—	—	Not detected; UV spectrum probably indicated thamnolic acid and fumarprotocetraric acid; see the three-lichen extract below
*Cladonia uncialis*	Usnic acid; ±squamatic acid	Usnic acid derivatives	7.573	343	Usnic acid
*Cetraria islandica*	Fumarprotocetraric acid; protocetraric acid; protolichesterinic acid	Higher aliphatic fatty acids	8.544	323	Protolichesterinic acid
Three-lichen extract	*C. arbuscula*: psoromic and usnic acids; *C. rangiferina*: fumarprotocetraric acid and atranorin; *C. digitata*: thamnolic acid	Usnic acid derivatives	7.582	343	Usnic acid
		β-orcinol depsidone	5.163	471	Fumarprotocetraric acid
		β-orcinol depside	7.582	373	Atranorin
		β-orcinol depside	5.163	419	Thamnolic acid
Five-lichen extract	*C. arbuscula*: psoromic and usnic acids; *C. rangiferina*: fumarprotocetraric acid and atranorin; *C. digitata*: thamnolic acid; *C. uncialis*: usnic acid and ±squamatic acid; *C. islandica*: fumarprotocetraric acid, protocetraric acid and protolichesterinic acid	Usnic acid derivatives	7.565	343	Usnic acid
		β-orcinol depside or β-orcinol depsidone	7.565	373	Atranorin, more likely, or protocetraric acid, less likely
*Pseudevernia furfuracea*	Physodic acid; atranorin	Orcinol depsidone	6.636	469	Physodic acid
			6.883		
			7.036		
			7.147		
		β-orcinol depside	7.607	373	Atranorin
		β-orcinol depside	7.905	407	Chloroatranorin

Notes: The three-lichen extract consisted of mixed thalli of *Cladonia arbuscula*, *C. rangiferina* and *C. digitata*. The five-lichen extract consisted of mixed thalli of *C. arbuscula*, *C. rangiferina*, *C. digitata*, *C. uncialis* and *Cetraria islandica*. A dash indicates that no corresponding metabolite assignment or m/z value was provided.

## Data Availability

Data are contained within the article and Supplementary Materials.

## Supplementary Materials

The following supporting information can be downloaded at https://www.mdpi.com/article/10.3390/molecules31162882/s1, Table S1: Composition of Hansen’s medium used to cultivate *Heterobasidion parviporum* mycelium (quantities per 1 L of distilled water); Table S2: Growth dynamics of *Heterobasidion parviporum* mycelium measured *in vitro* at 12 h intervals in cultures receiving 0–5 mL of acetone on the surface of solidified Hansen’s medium and incubated at 28 °C; Table S3: Growth dynamics of *Heterobasidion parviporum* mycelium measured *in vitro* at 12 h intervals in cultures treated with 1 and 2 mL of acetone extract from *Cladonia arbuscula* applied onto the surface of solidified Hansen’s medium and incubated at 28 °C; Table S4: Growth dynamics of *Heterobasidion parviporum* mycelium measured *in vitro* at 12 h intervals in cultures treated with 1 and 2 mL of acetone extract from *Cladonia rangiferina* applied onto the surface of solidified Hansen’s medium and incubated at 28 °C; Table S5: Growth dynamics of *Heterobasidion parviporum* mycelium measured *in vitro* at 12 h intervals in cultures treated with 1–3 mL of acetone extract from *Cladonia digitata* applied onto the surface of solidified Hansen’s medium and incubated at 28 °C; Table S6: Growth dynamics of *Heterobasidion parviporum* mycelium measured *in vitro* at 12 h intervals in cultures treated with 1–3 mL of acetone extract from *Cladonia uncialis* applied onto the surface of solidified Hansen’s medium and incubated at 28 °C; Table S7: Growth dynamics of *Heterobasidion parviporum* mycelium measured *in vitro* at 12 h intervals in cultures treated with 1–3 mL of acetone extract from *Cetraria islandica* applied onto the surface of solidified Hansen’s medium and incubated at 28 °C; Table S8: Growth dynamics of *Heterobasidion parviporum* mycelium measured *in vitro* at 12 h intervals in cultures treated with 1–3 mL of acetone extract prepared from mixed thalli of *Cladonia arbuscula* + *Cladonia rangiferina* + *Cladonia digitata*, applied onto the surface of solidified Hansen’s medium and incubated at 28 °C; Table S9: Growth dynamics of *Heterobasidion parviporum* mycelium measured *in vitro* at 12 h intervals in cultures treated with 1–3 mL of acetone extract prepared from mixed thalli of *Cladonia arbuscula* + *Cladonia rangiferina* + *Cladonia digitata* + *Cladonia uncialis* + *Cetraria islandica*, applied onto the surface of solidified Hansen’s medium and incubated at 28 °C; Table S10: Growth dynamics of *Heterobasidion parviporum* mycelium measured *in vitro* at 12 h intervals in cultures treated with 1–3 mL of acetone extract from *Pseudevernia furfuracea* applied onto the surface of solidified Hansen’s medium and incubated at 28 °C; Table S11: Growth dynamics of *Heterobasidion parviporum* mycelium measured *in vitro* at 12 h intervals in cultures receiving 0–4 mL of acetone incorporated into liquid Hansen’s medium before solidification and incubated at 28 °C; Table S12: Growth dynamics of *Heterobasidion parviporum* mycelium measured *in vitro* at 12 h intervals in cultures treated with 1–4 mL of acetone extract from *Cladonia arbuscula* incorporated into liquid Hansen’s medium before solidification and incubated at 28 °C; Table S13: Growth dynamics of *Heterobasidion parviporum* mycelium measured *in vitro* at 12 h intervals in cultures treated with 1–4 mL of acetone extract from *Cladonia rangiferina* incorporated into liquid Hansen’s medium before solidification and incubated at 28 °C; Table S14: Growth dynamics of *Heterobasidion parviporum* mycelium measured *in vitro* at 12 h intervals in cultures treated with 1–4 mL of acetone extract from *Cladonia digitata* incorporated into liquid Hansen’s medium before solidification and incubated at 28 °C; Table S15: Growth dynamics of *Heterobasidion parviporum* mycelium measured *in vitro* at 12 h intervals in cultures treated with 1–4 mL of acetone extract from *Cladonia uncialis* incorporated into liquid Hansen’s medium before solidification and incubated at 28 °C; Table S16: Growth dynamics of *Heterobasidion parviporum* mycelium measured *in vitro* at 12 h intervals in cultures treated with 1–4 mL of acetone extract from *Cetraria islandica* incorporated into liquid Hansen’s medium before solidification and incubated at 28 °C; Table S17: Growth dynamics of *Heterobasidion parviporum* mycelium measured *in vitro* at 12 h intervals in cultures treated with 1–4 mL of acetone extract prepared from mixed thalli of *Cladonia arbuscula* + *Cladonia rangiferina* + *Cladonia digitata*, incorporated into liquid Hansen’s medium before solidification and incubated at 28 °C; Table S18: Growth dynamics of *Heterobasidion parviporum* mycelium measured *in vitro* at 12 h intervals in cultures treated with 1–4 mL of acetone extract prepared from mixed thalli of *Cladonia arbuscula* + *Cladonia rangiferina* + *Cladonia digitata* + *Cladonia uncialis* + *Cetraria islandica*, incorporated into liquid Hansen’s medium before solidification and incubated at 28 °C; Table S19: Growth dynamics of *Heterobasidion parviporum* mycelium measured *in vitro* at 12 h intervals in cultures treated with 1–4 mL of acetone extract from *Pseudevernia furfuracea* incorporated into liquid Hansen’s medium before solidification and incubated at 28 °C; Table S20: Comparison of *Heterobasidion parviporum* mycelial growth dynamics between cultures treated with eight lichen acetone-extract variants and cultures receiving equivalent acetone volumes (1–3 mL), applied onto the surface of solidified Hansen’s medium, incubated at 28 °C, and measured at 12 h intervals; Table S21: Comparison of *Heterobasidion parviporum* mycelial growth dynamics between cultures treated with eight lichen acetone-extract variants and cultures receiving equivalent acetone volumes (1–4 mL), incorporated into liquid Hansen’s medium before solidification, incubated at 28 °C, and measured at 12 h intervals; Table S22: Comparison of *Heterobasidion parviporum* mycelial growth dynamics between application onto the surface of solidified Hansen’s medium and incorporation into the liquid medium before solidification: (A) solvent-free controls and (B) cultures receiving equivalent volumes (1–3 mL) of acetone or eight lichen acetone-extract variants. Cultures were incubated at 28 °C and measured at 12 h intervals; Figure S1: UPLC–PDA–ESI–MS analysis of the acetone extract from *Cladonia arbuscula*. (A) UPLC chromatogram; (B) negative-ion mass spectrum at Rt = 7.599 min. KUS, usnic acid; Figure S2: UPLC–PDA–ESI–MS analysis of the acetone extract from *Cladonia rangiferina*. (A) UPLC chromatogram; (B) negative-ion mass spectrum at Rt = 7.599 min. ATR, atranorin; Figure S3: UPLC–PDA–ESI–MS analysis of the acetone extract from *Cladonia digitata*. (A) UPLC chromatogram; (B–E) negative-ion mass spectra at Rt = 5.119, 6.432, 6.934, and 7.514 min, respectively; Figure S4: UPLC–PDA–ESI–MS analysis of the acetone extract from *Cladonia uncialis*. (A) UPLC chromatogram; (B) negative-ion mass spectrum at Rt = 7.573 min. KUS, usnic acid; Figure S5: UPLC–PDA–ESI–MS analysis of the acetone extract from *Cetraria islandica*. (A) UPLC chromatogram; (B) negative-ion mass spectrum at Rt = 8.544 min. KPS, protolichesterinic acid; Figure S6: UPLC–PDA–ESI–MS analysis of the acetone extract prepared from mixed thalli of *Cladonia arbuscula* + *Cladonia rangiferina* + *Cladonia digitata*. (A) UPLC chromatogram; (B,C) negative-ion mass spectra at Rt = 5.163 and 7.582 min, respectively. ATR, atranorin; KFP, fumarprotocetraric acid; KTL, thamnolic acid; KUS, usnic acid; Figure S7: UPLC–PDA–ESI–MS analysis of the acetone extract prepared from mixed thalli of *Cladonia arbuscula* + *Cladonia rangiferina* + *Cladonia digitata* + *Cladonia uncialis* + *Cetraria islandica*. (A) UPLC chromatogram; (B) negative-ion mass spectrum at Rt = 7.565 min. ATR/KPC, atranorin or protocetraric acid; KUS, usnic acid; Figure S8: UPLC–PDA–ESI–MS analysis of the acetone extract from *Pseudevernia furfuracea*. (A) UPLC chromatogram; (B–G) negative-ion mass spectra at Rt = 6.636, 6.883, 7.036, 7.147, 7.607, and 7.905 min, respectively. ATR, atranorin; KFD, physodic acid; Figure S9: Growth dynamics (mean colony diameter) of *Heterobasidion parviporum* mycelium at 28 °C in cultures treated with 1 and 2 mL of acetone or acetone extract from *Cladonia arbuscula* (N = 5 per treatment), with both treatments applied onto the surface of solidified Hansen’s medium; Figure S10: Growth dynamics (mean colony diameter) of *Heterobasidion parviporum* mycelium at 28 °C in cultures treated with 1–4 mL of acetone or acetone extract from *Cladonia arbuscula* (N = 5 per treatment), with both treatments incorporated into liquid Hansen’s medium before solidification; Figure S11: Growth dynamics (mean colony diameter) of *Heterobasidion parviporum* mycelium at 28 °C in cultures treated with 1 and 2 mL of acetone or acetone extract from *Cladonia rangiferina* (N = 5 per treatment), with both treatments applied onto the surface of solidified Hansen’s medium; Figure S12: Growth dynamics (mean colony diameter) of *Heterobasidion parviporum* mycelium at 28 °C in cultures treated with 1–4 mL of acetone or acetone extract from *Cladonia rangiferina* (N = 5 per treatment), with both treatments incorporated into liquid Hansen’s medium before solidification; Figure S13: Growth dynamics (mean colony diameter) of *Heterobasidion parviporum* mycelium at 28 °C in cultures treated with 1–3 mL of acetone or acetone extract from *Cladonia digitata* (N = 5 per treatment), with both treatments applied onto the surface of solidified Hansen’s medium; Figure S14: Growth dynamics (mean colony diameter) of *Heterobasidion parviporum* mycelium at 28 °C in cultures treated with 1–4 mL of acetone or acetone extract from *Cladonia digitata* (N = 5 per treatment), with both treatments incorporated into liquid Hansen’s medium before solidification; Figure S15: Growth dynamics (mean colony diameter) of *Heterobasidion parviporum* mycelium at 28 °C in cultures treated with 1–3 mL of acetone or acetone extract from *Cladonia uncialis* (N = 5 per treatment), with both treatments applied onto the surface of solidified Hansen’s medium; Figure S16: Growth dynamics (mean colony diameter) of *Heterobasidion parviporum* mycelium at 28 °C in cultures treated with 1–4 mL of acetone or acetone extract from *Cladonia uncialis* (N = 5 per treatment), with both treatments incorporated into liquid Hansen’s medium before solidification; Figure S17: Growth dynamics (mean colony diameter) of *Heterobasidion parviporum* mycelium at 28 °C in cultures treated with 1–4 mL of acetone or acetone extract from *Cetraria islandica* (N = 5 per treatment), with both treatments incorporated into liquid Hansen’s medium before solidification; Figure S18: Growth dynamics (mean colony diameter) of *Heterobasidion parviporum* mycelium at 28 °C in cultures treated with 1–3 mL of acetone or acetone extract prepared from mixed thalli of *Cladonia arbuscula* + *Cladonia rangiferina* + *Cladonia digitata* (N = 5 per treatment), with both treatments applied onto the surface of solidified Hansen’s medium; Figure S19: Growth dynamics (mean colony diameter) of *Heterobasidion parviporum* mycelium at 28 °C in cultures treated with 1–4 mL of acetone or acetone extract prepared from mixed thalli of *Cladonia arbuscula* + *Cladonia rangiferina* + *Cladonia digitata* (N = 5 per treatment), with both treatments incorporated into liquid Hansen’s medium before solidification; Figure S20: Growth dynamics (mean colony diameter) of *Heterobasidion parviporum* mycelium at 28 °C in cultures treated with 1–4 mL of acetone or acetone extract prepared from mixed thalli of *Cladonia arbuscula* + *Cladonia rangiferina* + *Cladonia digitata* + *Cladonia uncialis* + *Cetraria islandica* (N = 5 per treatment), with both treatments incorporated into liquid Hansen’s medium before solidification; Figure S21: Growth dynamics (mean colony diameter) of *Heterobasidion parviporum* mycelium at 28 °C in cultures treated with 1–4 mL of acetone or acetone extract from *Pseudevernia furfuracea* (N = 5 per treatment), with both treatments incorporated into liquid Hansen’s medium before solidification.
