# Supplementary material for: Antifungal and Profungal Potentials of Acetone-Extracted Complexes of Lichen Substances on Macromycete Heterobasidion parviporum Mycelium Growth Dynamics—The First In Vitro Approach
Source: Molecules. 2026 Aug 18;31(16):2882. doi: 10.3390/molecules31162882 (PMC13515570; doi:10.3390/molecules31162882)
Supplement: Supplementary file 1 [file molecules-31-02882-s001.zip › Figures_S1-S8_UPLC-PDA-ESI-MS_Analyses.pdf]

Figure S1: UPLC–PDA–ESI–MS analysis of the acetone extract from *Cladonia arbuscula*. (A) UPLC chromatogram; (B) negative-ion mass spectrum at Rt = 7.599 min. KUS, usnic acid.

A

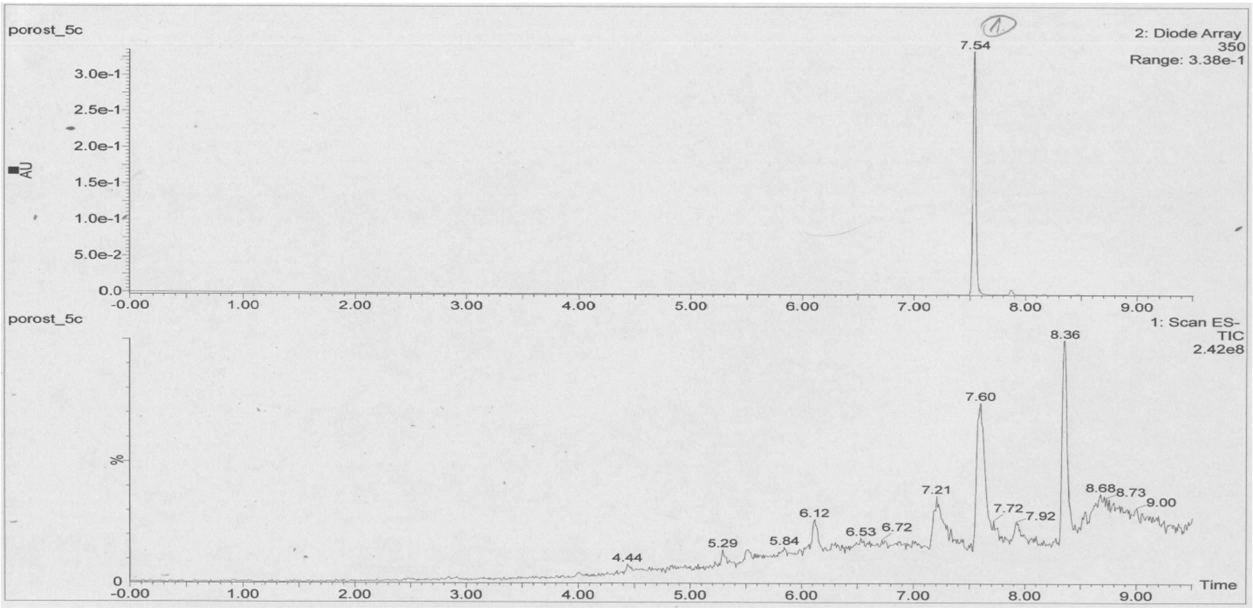

B

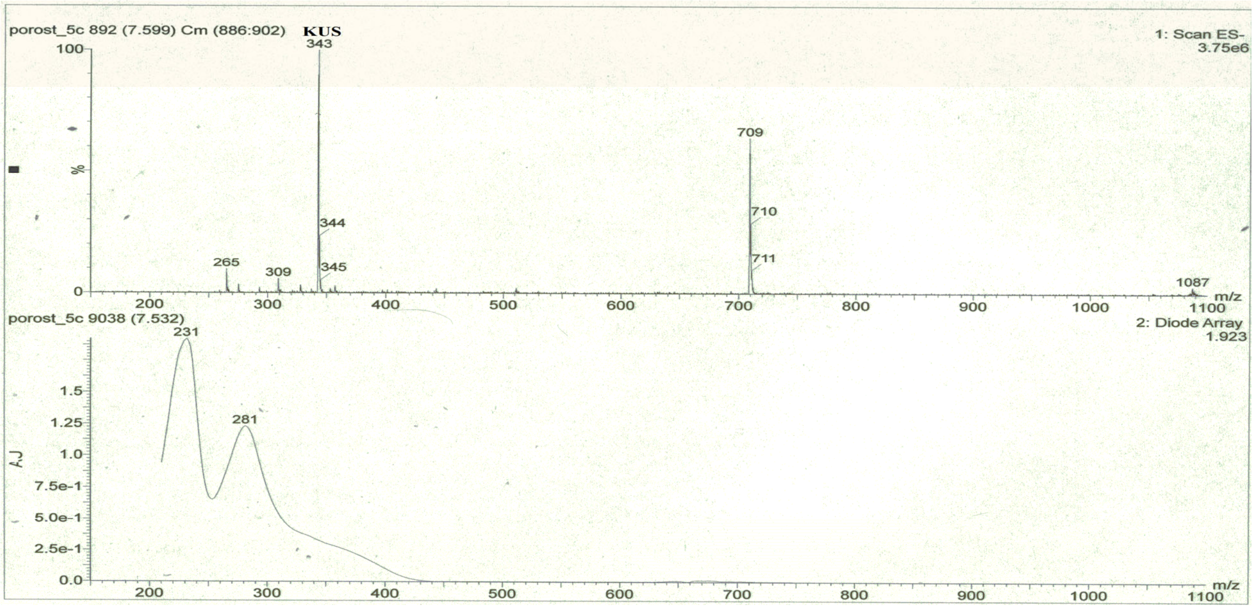

Figure S2: UPLC–PDA–ESI–MS analysis of the acetone extract from *Cladonia rangiferina*. (A) UPLC chromatogram; (B) negative-ion mass spectrum at Rt = 7.599 min. ATR, atranorin.

A

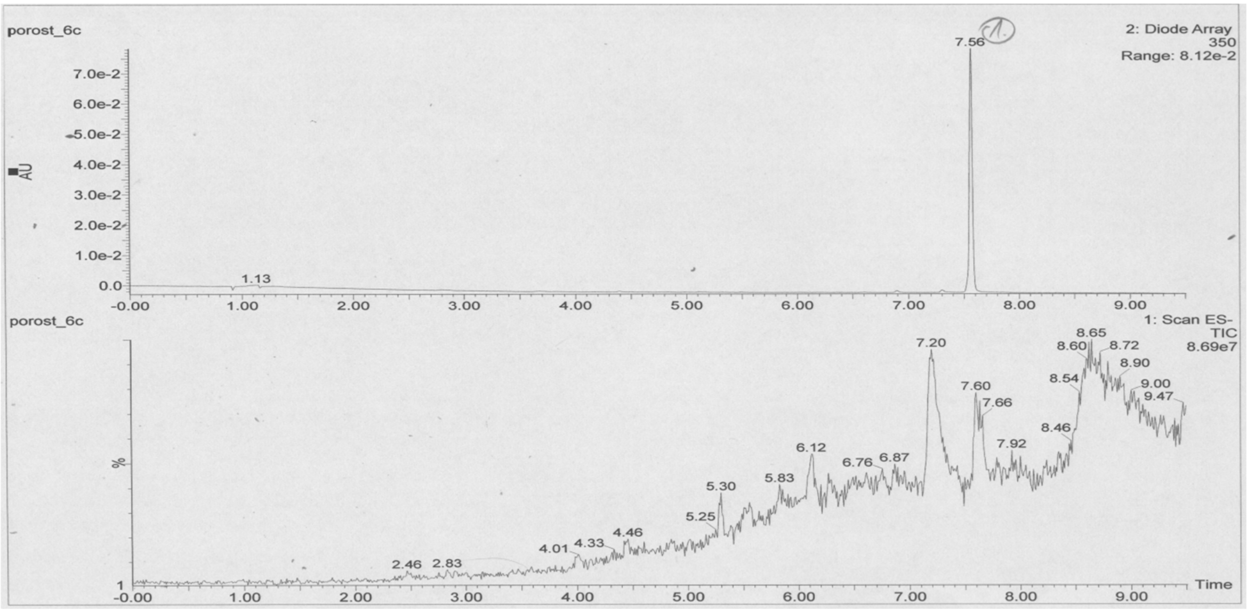

B

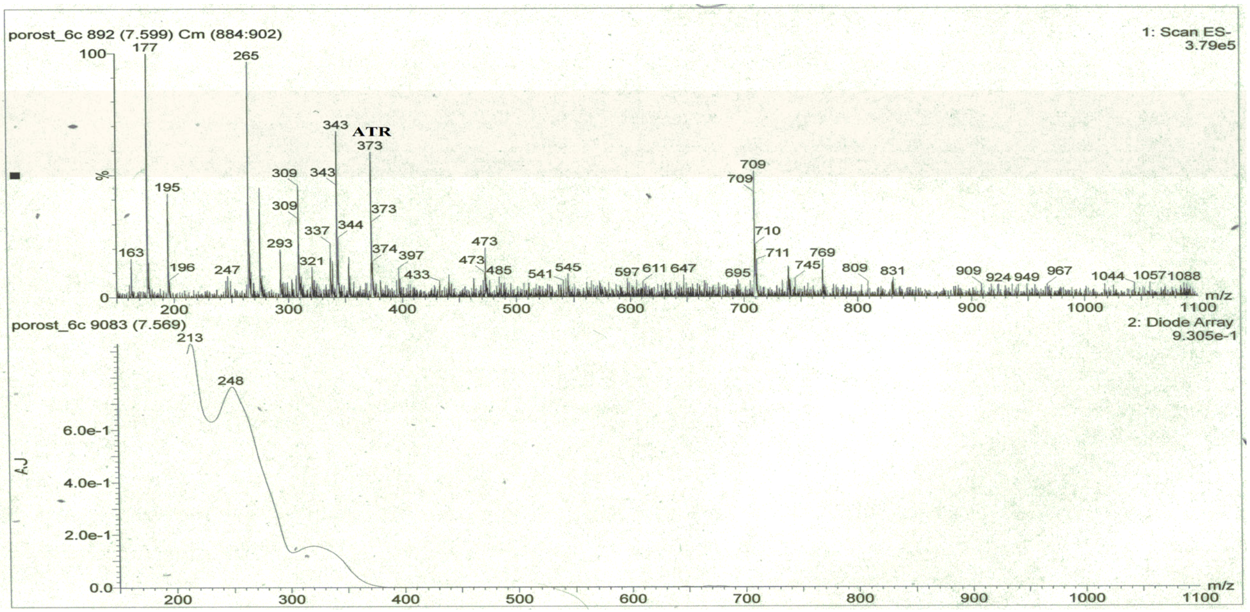

Figure S3: UPLC–PDA–ESI–MS analysis of the acetone extract from *Cladonia digitata*. (A) UPLC chromatogram; (B–E) negative-ion mass spectra at Rt = 5.119, 6.432, 6.934, and 7.514 min, respectively.

A

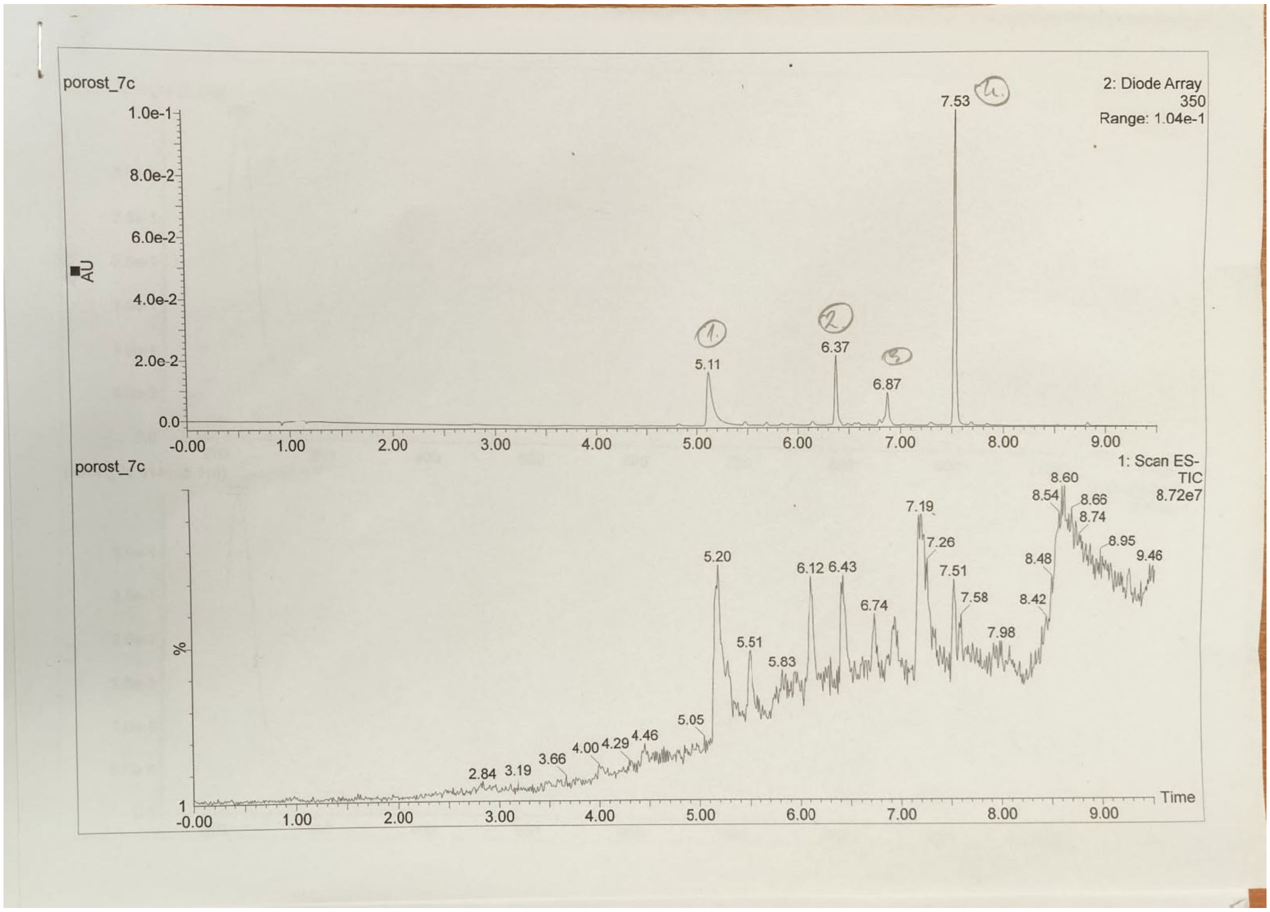

B

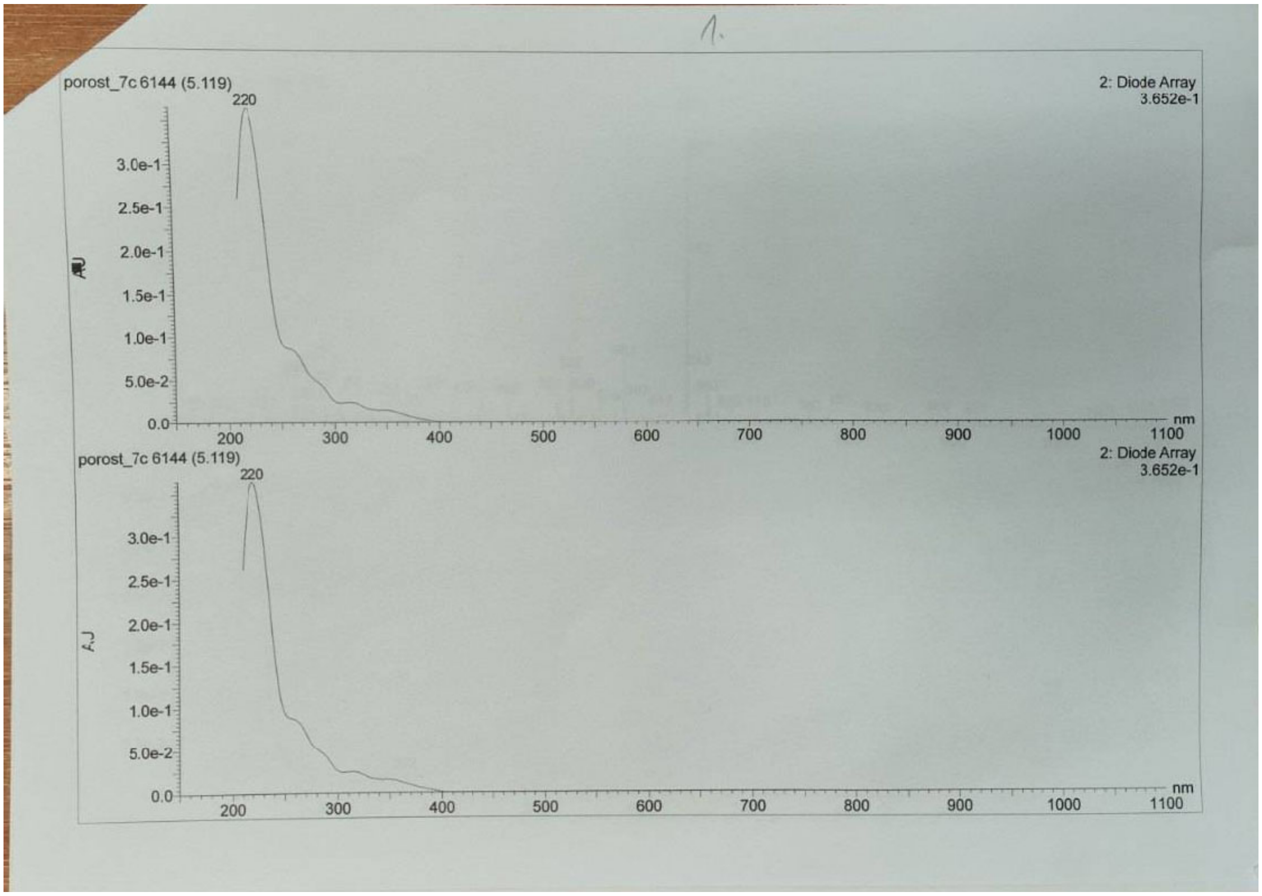

1.
porost_7c 6144 (5.119)
2: Diode Array
3.652e-1
220
3.0e-1
2.5e-1
2.0e-1
1.5e-1
1.0e-1
5.0e-2
0.0
AU
nm
200
300
400
500
600
700
800
900
1000
1100
porost_7c 6144 (5.119)
2: Diode Array
3.652e-1
220

C

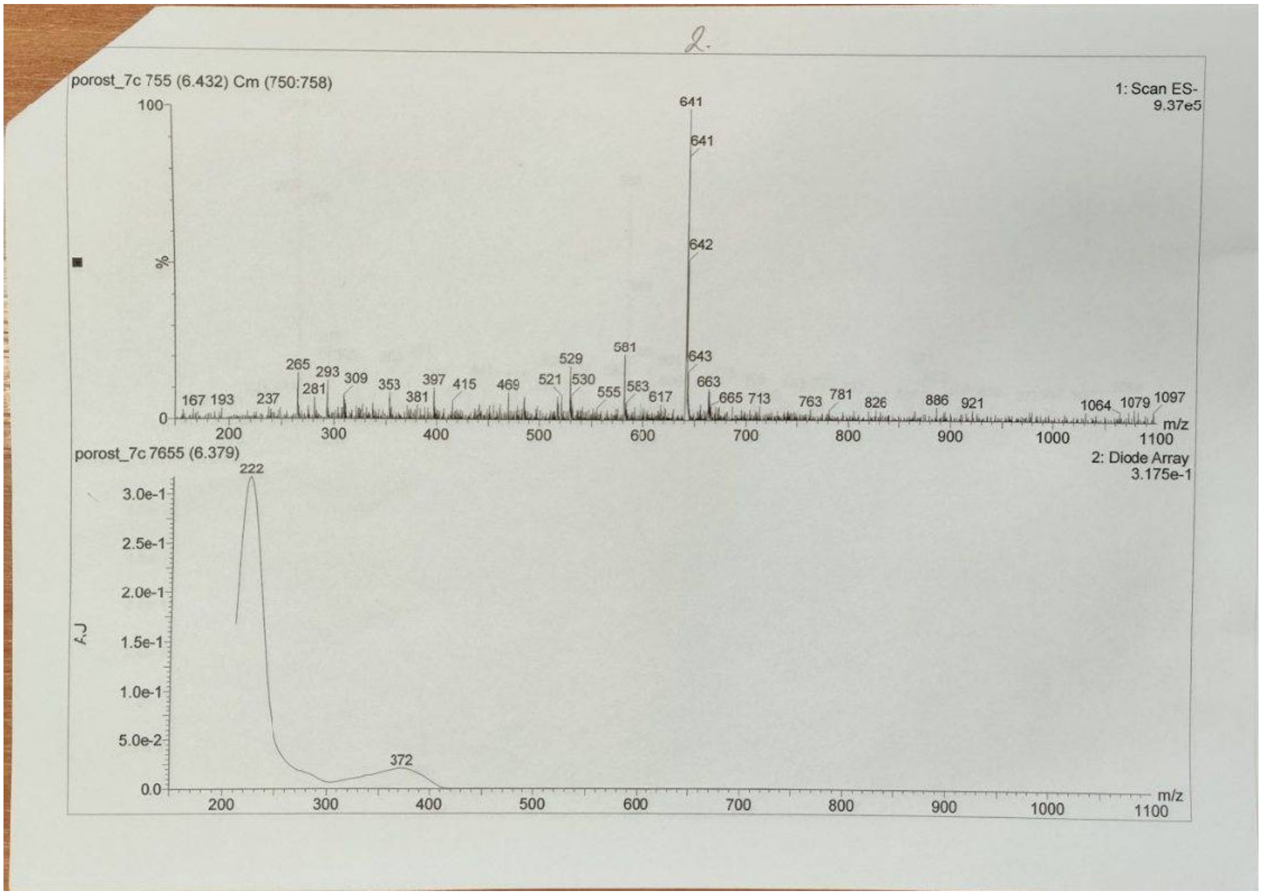

2.
porost_7c 755 (6.432) Cm (750:758)
1: Scan ES-
9.37e5
100
%
0
641
642
643
581
529
530
265
293
309
353
397
415
469
521
555
563
617
663
665
713
763
781
826
886
921
1064
1079
1097
167
193
237
281
381
m/z
200
300
400
500
600
700
800
900
1000
1100
porost_7c 7655 (6.379)
2: Diode Array
3.175e-1
222
372
3.0e-1
2.5e-1
2.0e-1
1.5e-1
1.0e-1
5.0e-2
0.0

D

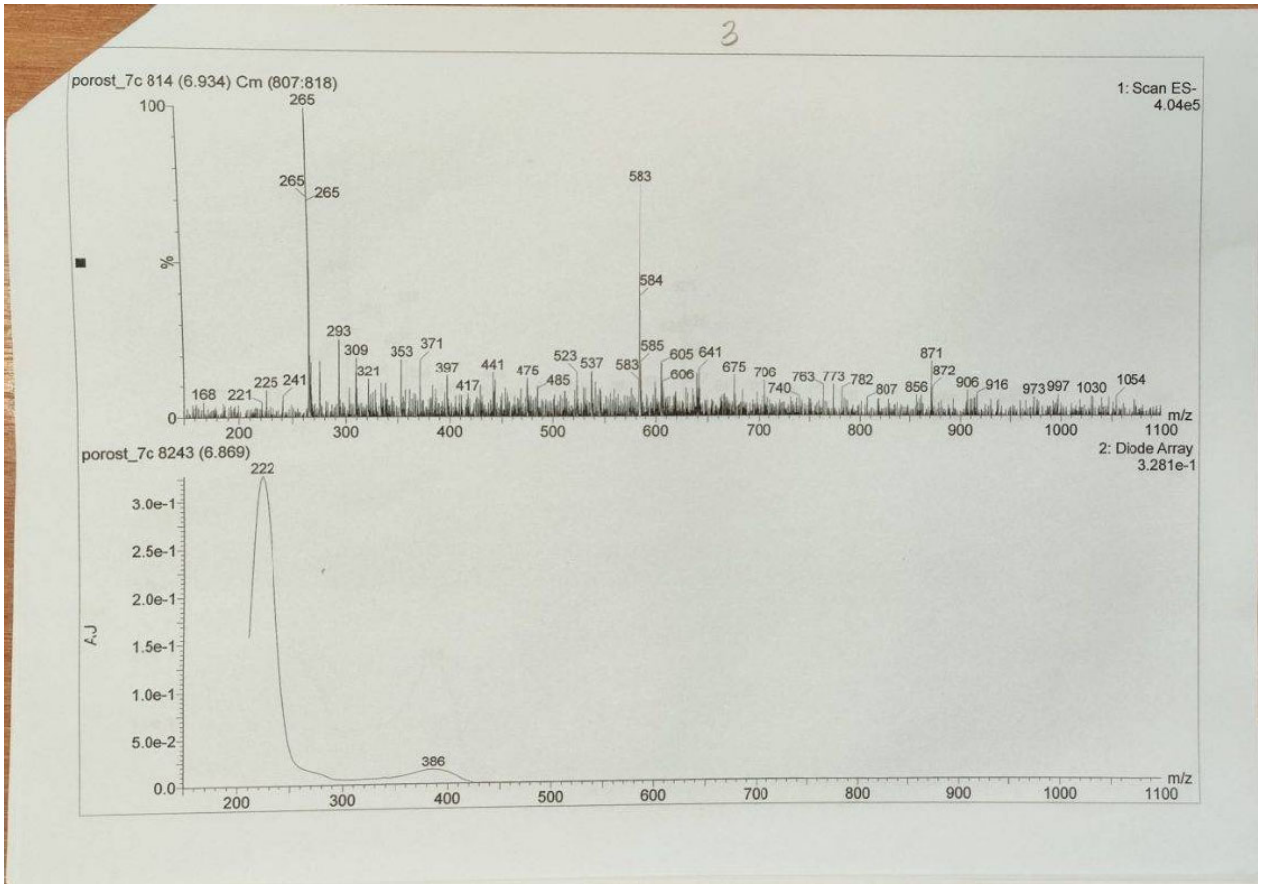

3
porost_7c 814 (6.934) Cm (807:818)
1: Scan ES-
4.04e5
%
m/z
porost_7c 8243 (6.869)
2: Diode Array
3.281e-1
AU
m/z

E

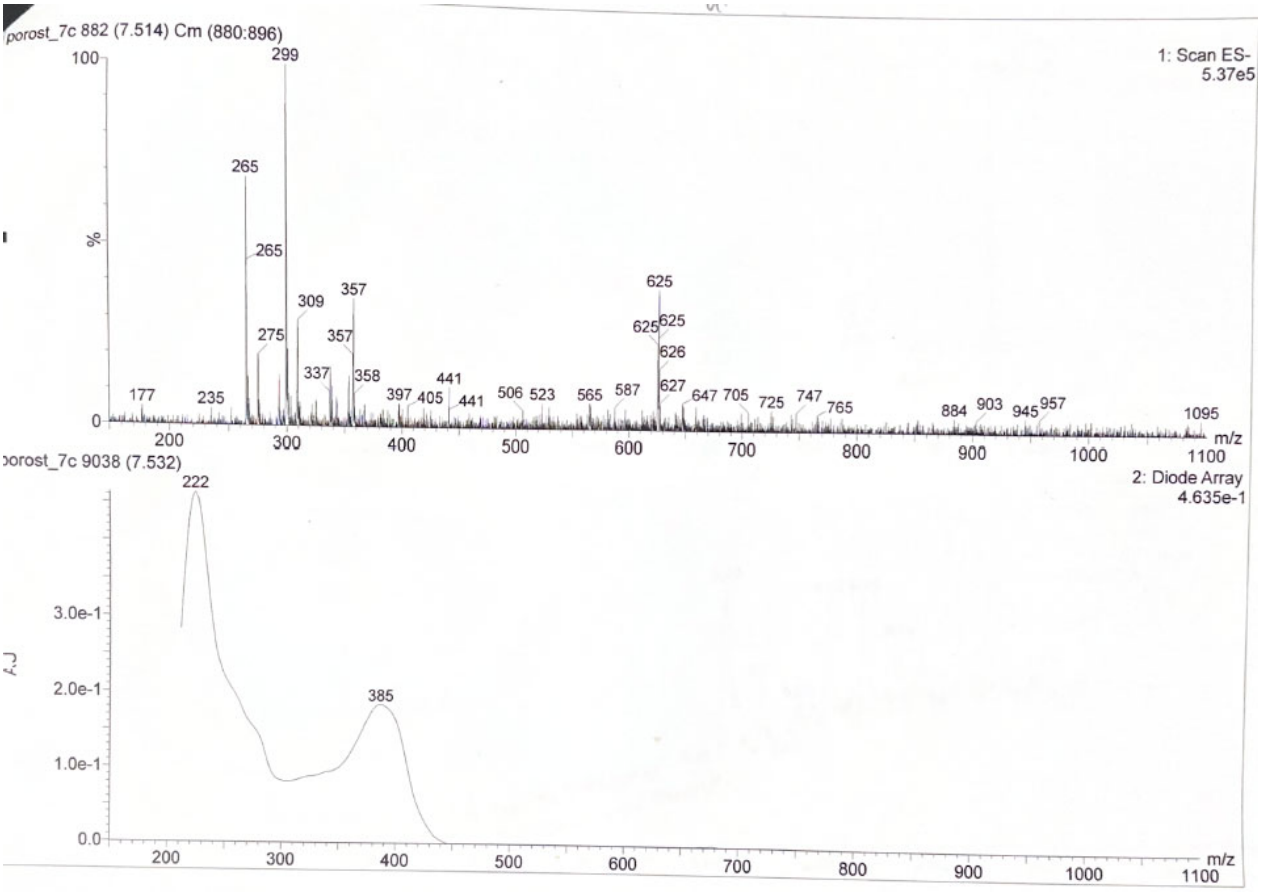
porost_7c 882 (7.514) Cm (880:896)
1: Scan ES-
5.37e5
100
%
0
299
265
265
357
309
275
357
337
358
441
397
405
441
506
523
565
587
625
625
625
626
627
647
705
725
747
765
177
235
884
903
945
957
1095
200
300
400
500
600
700
800
900
1000
1100
m/z
porost_7c 9038 (7.532)
2: Diode Array
4.635e-1
222
3.0e-1
2.0e-1
1.0e-1
0.0
385
m/z

Figure S4: UPLC–PDA–ESI–MS analysis of the acetone extract from *Cladonia uncialis*. (A) UPLC chromatogram; (B) negative-ion mass spectrum at Rt = 7.573 min. KUS, usnic acid.

A

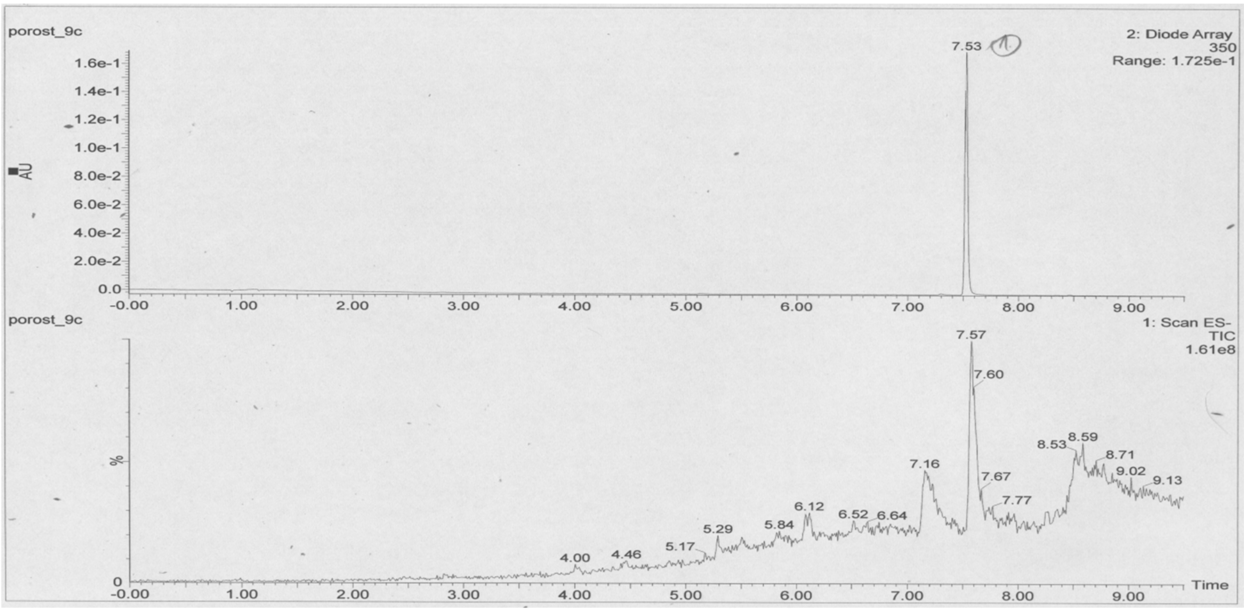

B

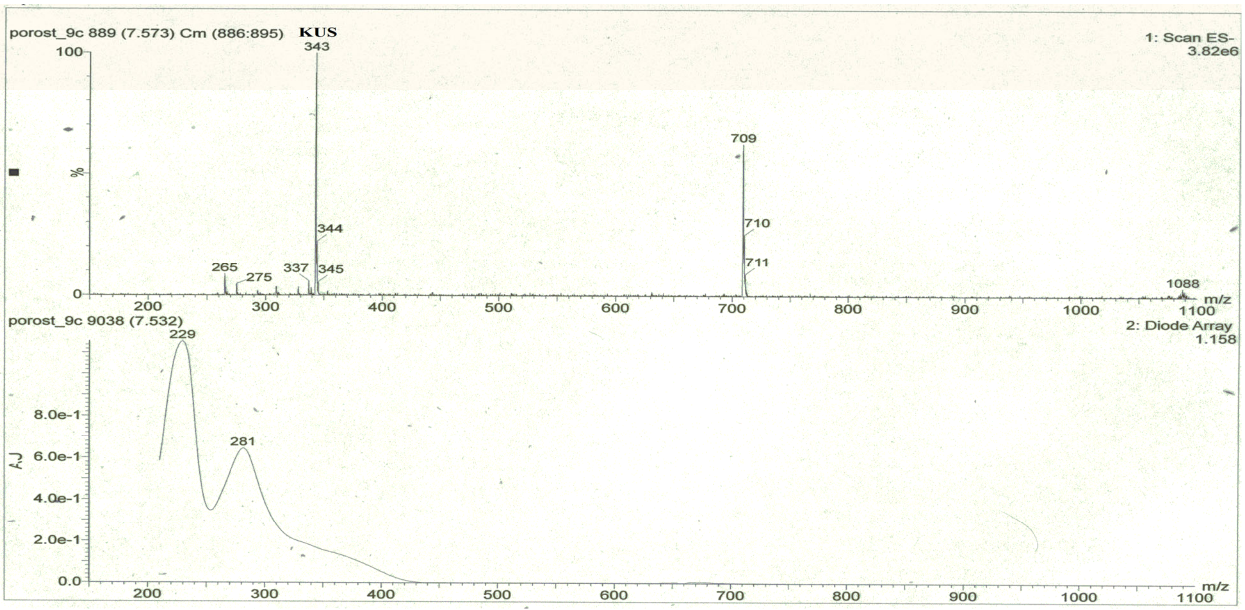

Figure S5: UPLC–PDA–ESI–MS analysis of the acetone extract from *Cetraria islandica*. (A) UPLC chromatogram; (B) negative-ion mass spectrum at Rt = 8.544 min. KPS, protolichesterinic acid.

A

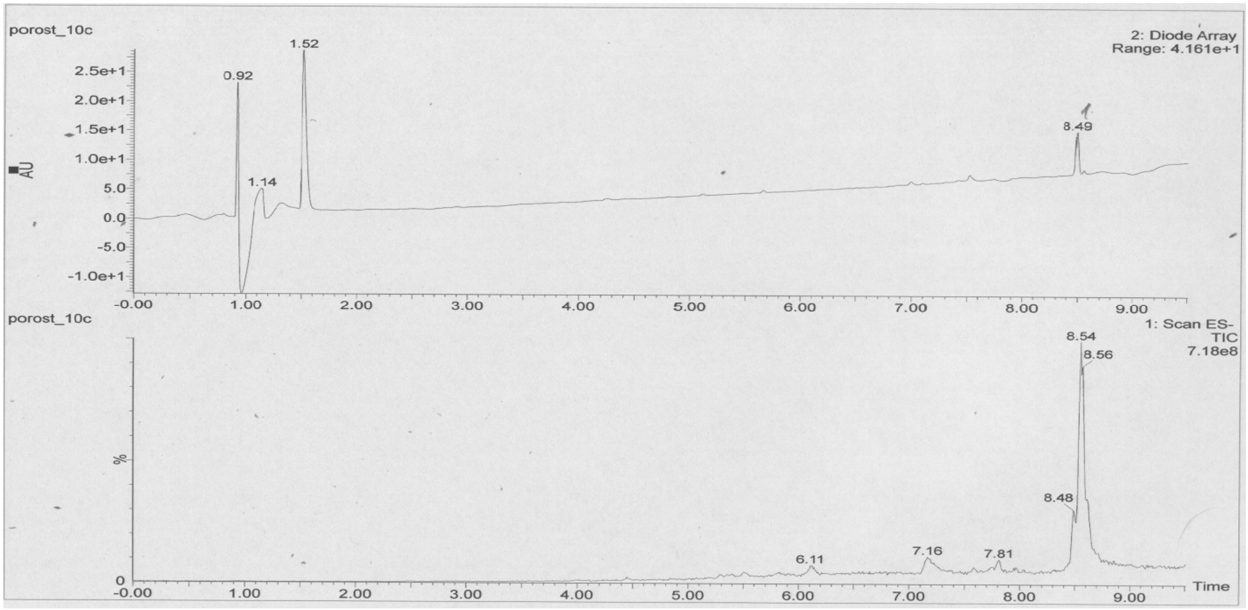

B

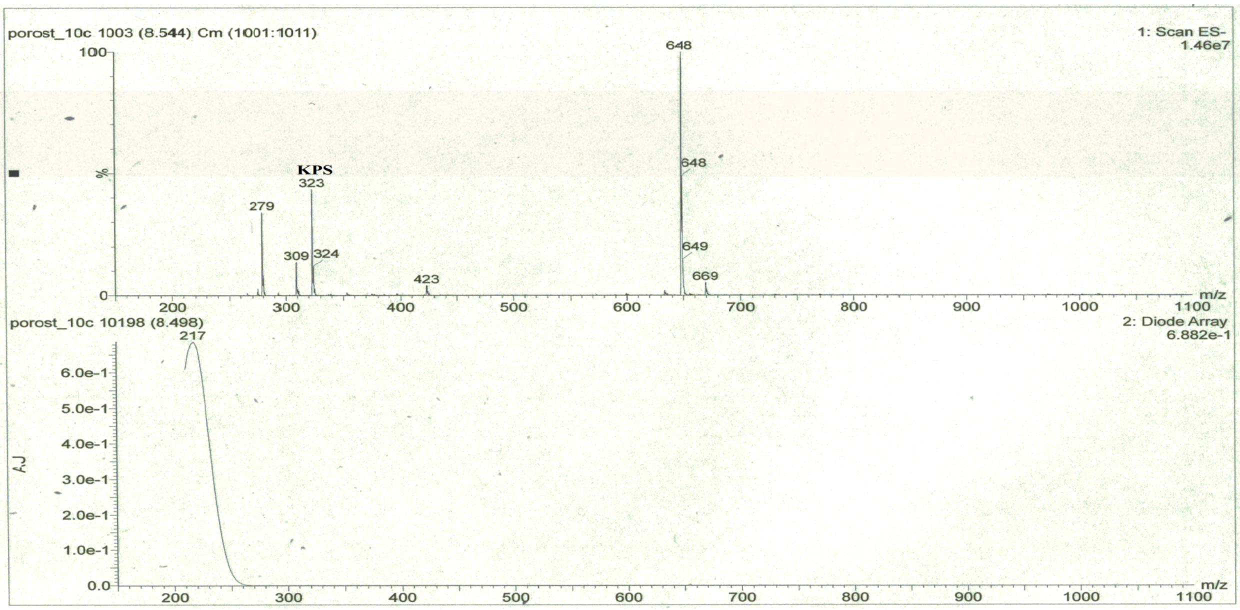

Figure S6: UPLC–PDA–ESI–MS analysis of the acetone extract prepared from mixed thalli of *Cladonia arbuscula* + *Cladonia rangiferina* + *Cladonia digitata*. (A) UPLC chromatogram; (B,C) negative-ion mass spectra at Rt = 5.163 and 7.582 min, respectively. ATR, atranorin; KFP, fumarprotocetraric acid; KTL, thamnolic acid; KUS, usnic acid.

A

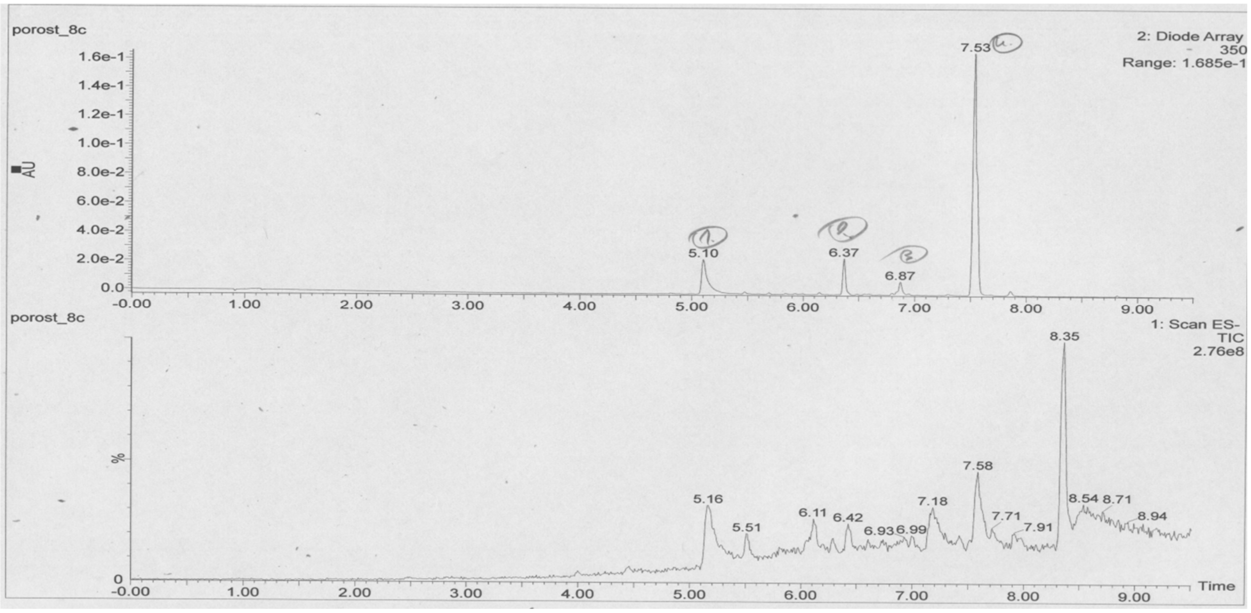

B

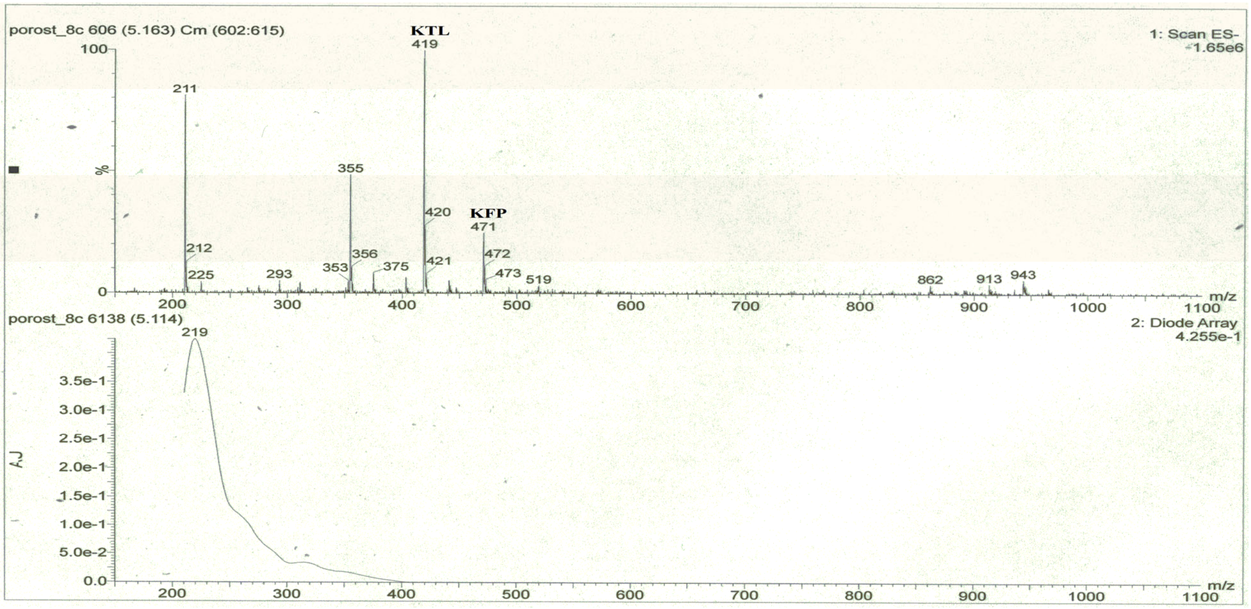

C

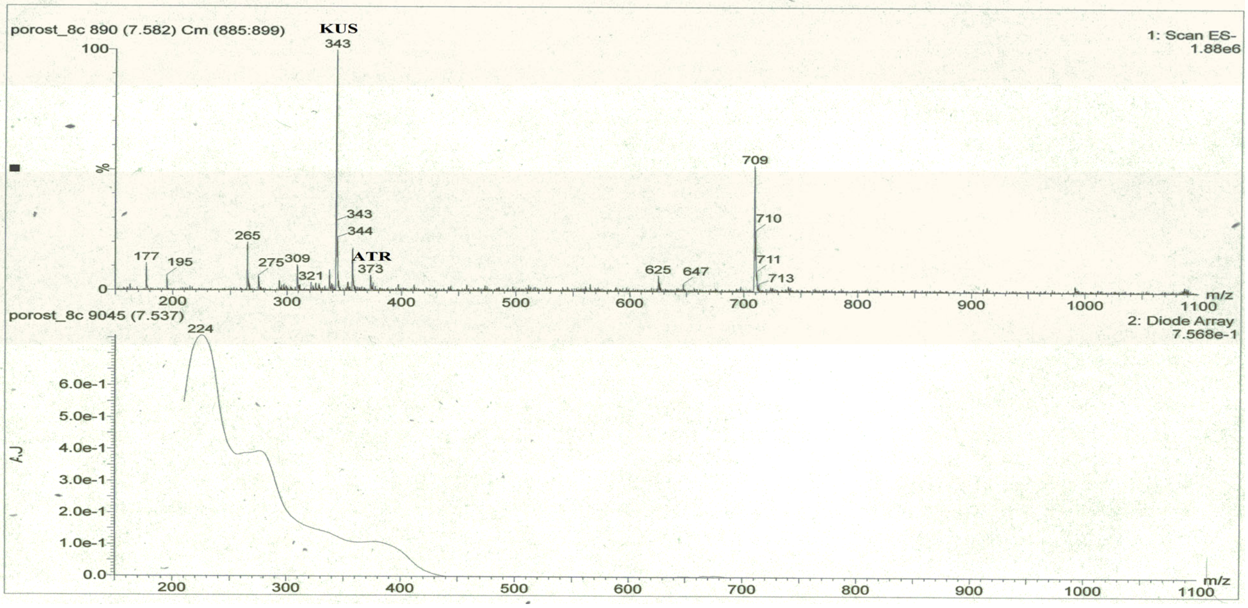

porost_8c 890 (7.582) Cm (885:899)
KUS
343
1: Scan ES-
1.88e6
709
710
343
344
265
ATR
373
177
195
275
309
321
625
647
711
713
100
%
0
m/z
porost_8c 9045 (7.537)
224
2: Diode Array
7.568e-1
6.0e-1
5.0e-1
4.0e-1
3.0e-1
2.0e-1
1.0e-1
0.0
AU
200
300
400
500
600
700
800
900
1000
1100
m/z

Figure S7: UPLC–PDA–ESI–MS analysis of the acetone extract prepared from mixed thalli of *Cladonia arbuscula* + *Cladonia rangiferina* + *Cladonia digitata* + *Cladonia uncialis* + *Cetraria islandica*. (A) UPLC chromatogram; (B) negative-ion mass spectrum at Rt = 7.565 min. ATR/KPC, atranorin or protocetraric acid; KUS, usnic acid.

A

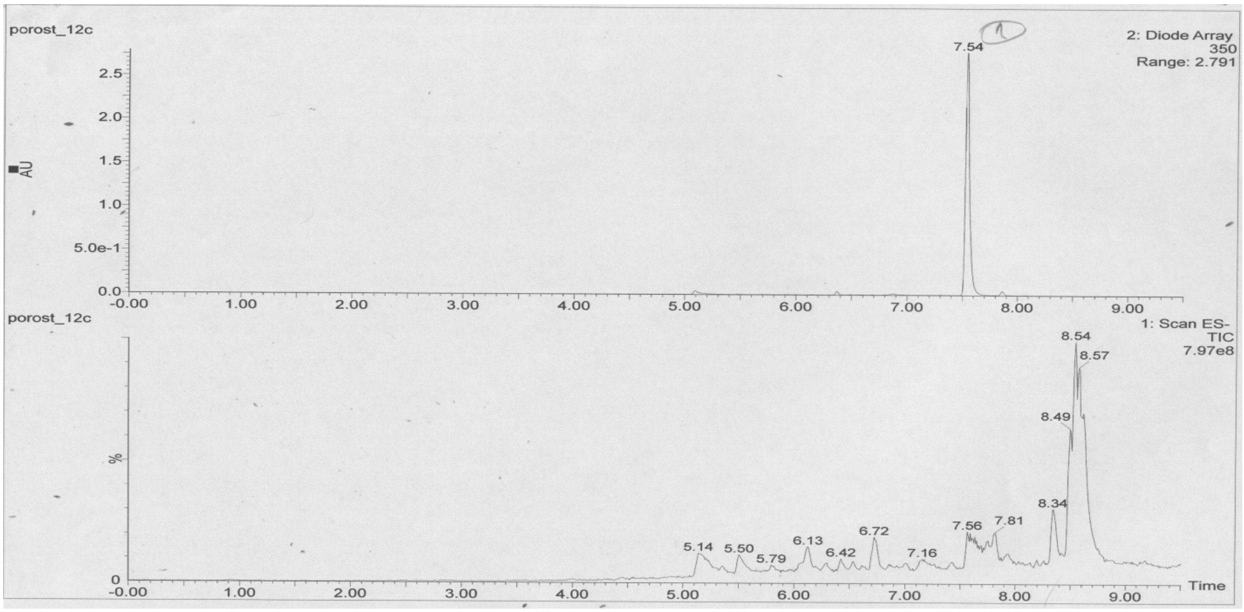

B

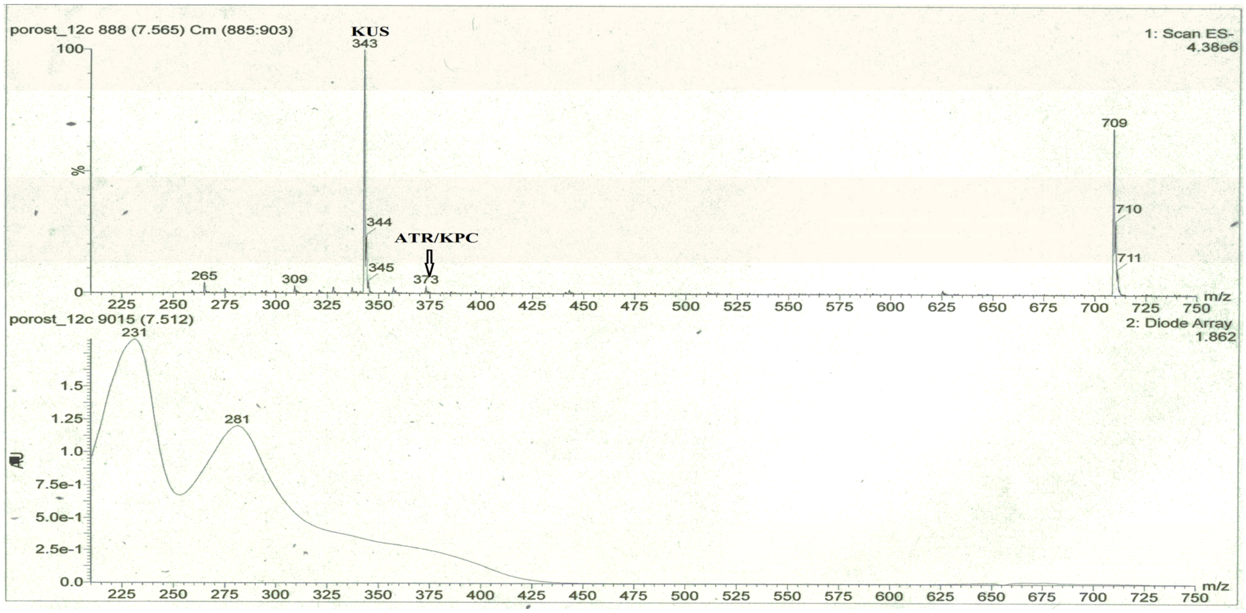

Figure S8: UPLC–PDA–ESI–MS analysis of the acetone extract from *Pseudevernia furfuracea*. (A) UPLC chromatogram; (B–G) negative-ion mass spectra at Rt = 6.636, 6.883, 7.036, 7.147, 7.607, and 7.905 min, respectively. ATR, atranorin; KFD, physodic acid.

A

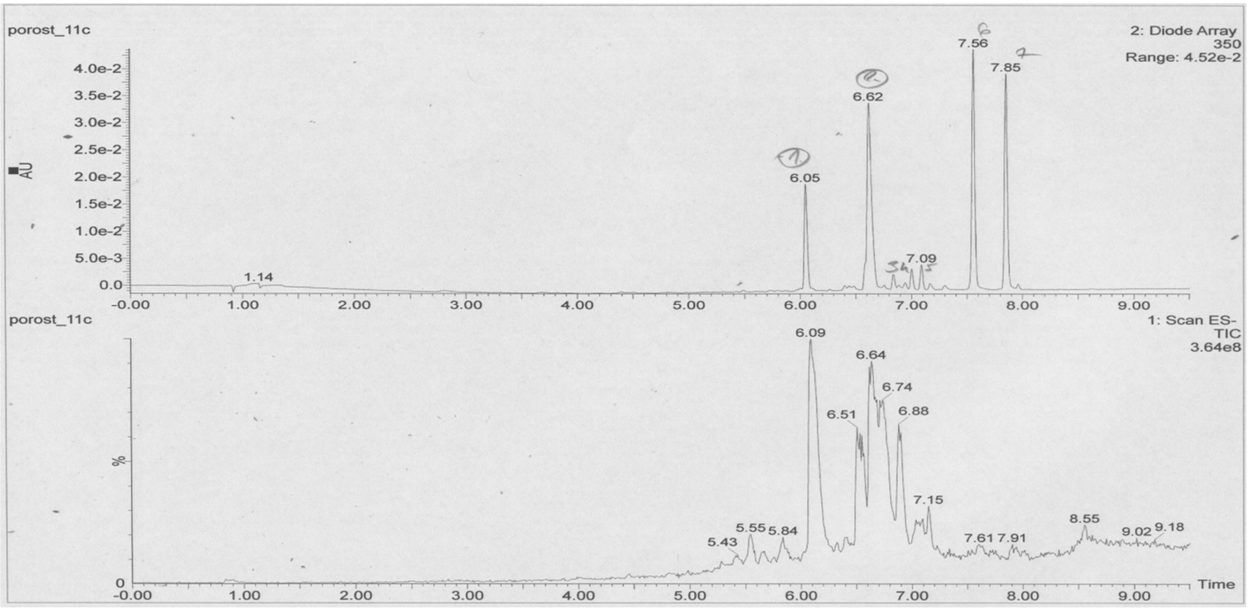

B

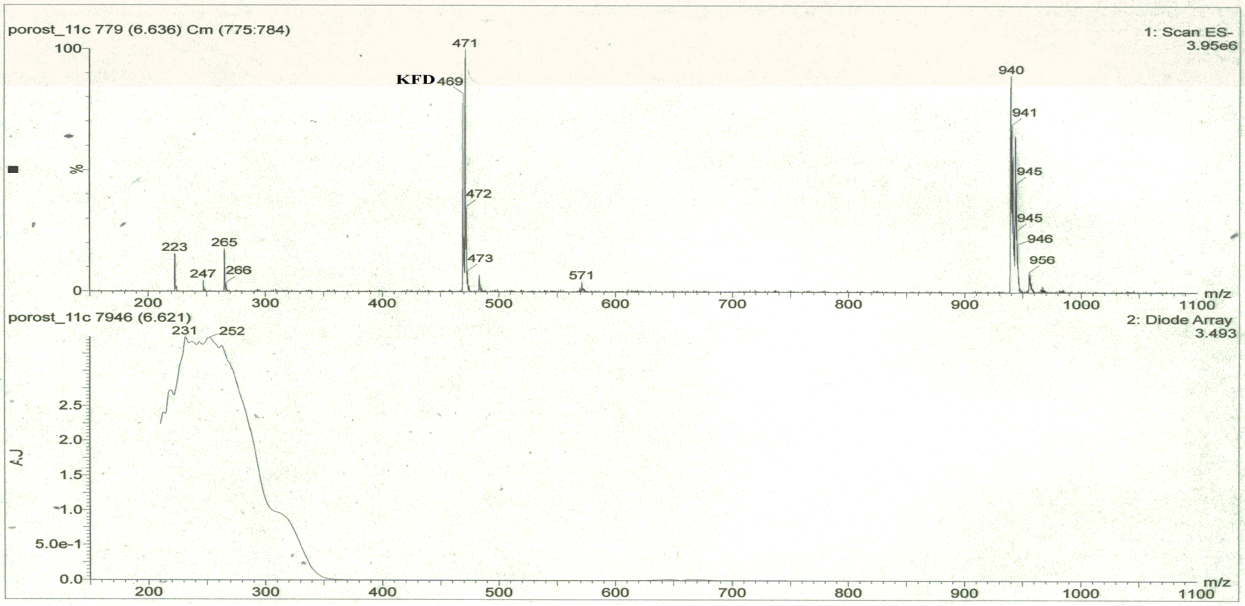

C

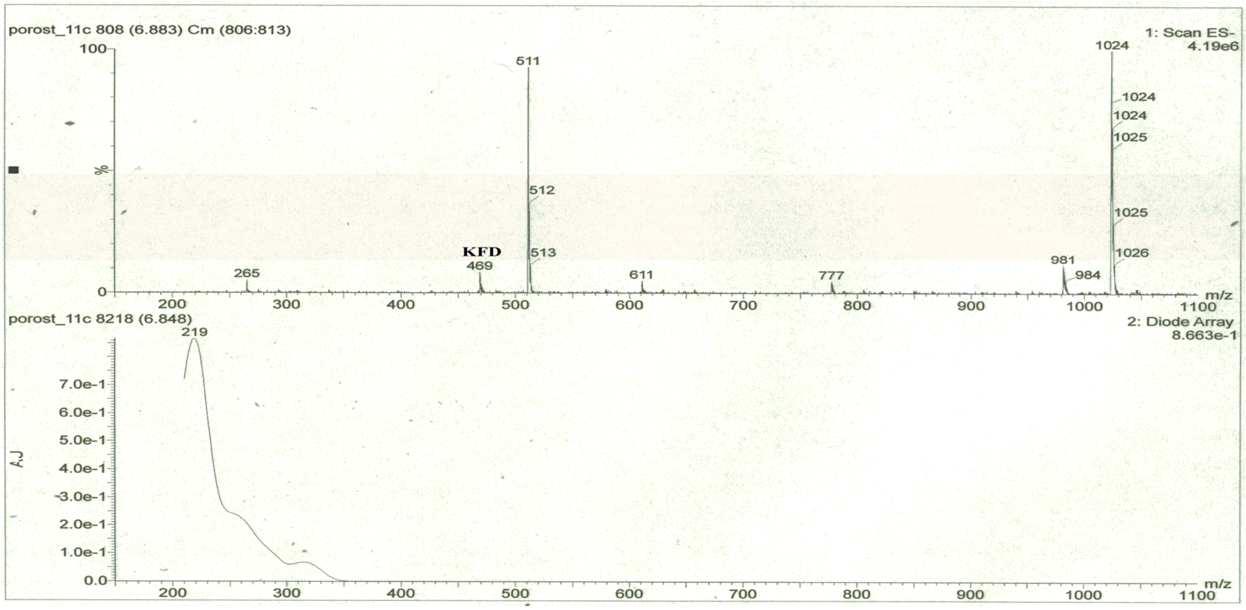
porost_11c 808 (6.883) Cm (806:813)
1: Scan ES-
4.19e6
100
%
0
511
512
513
KFD
469
265
611
777
981
984
1024
1024
1024
1025
1025
1026
m/z
200
300
400
500
600
700
800
900
1000
1100
porost_11c 8218 (6.848)
2: Diode Array
8.663e-1
219
7.0e-1
6.0e-1
5.0e-1
4.0e-1
3.0e-1
2.0e-1
1.0e-1
0.0
AU

D

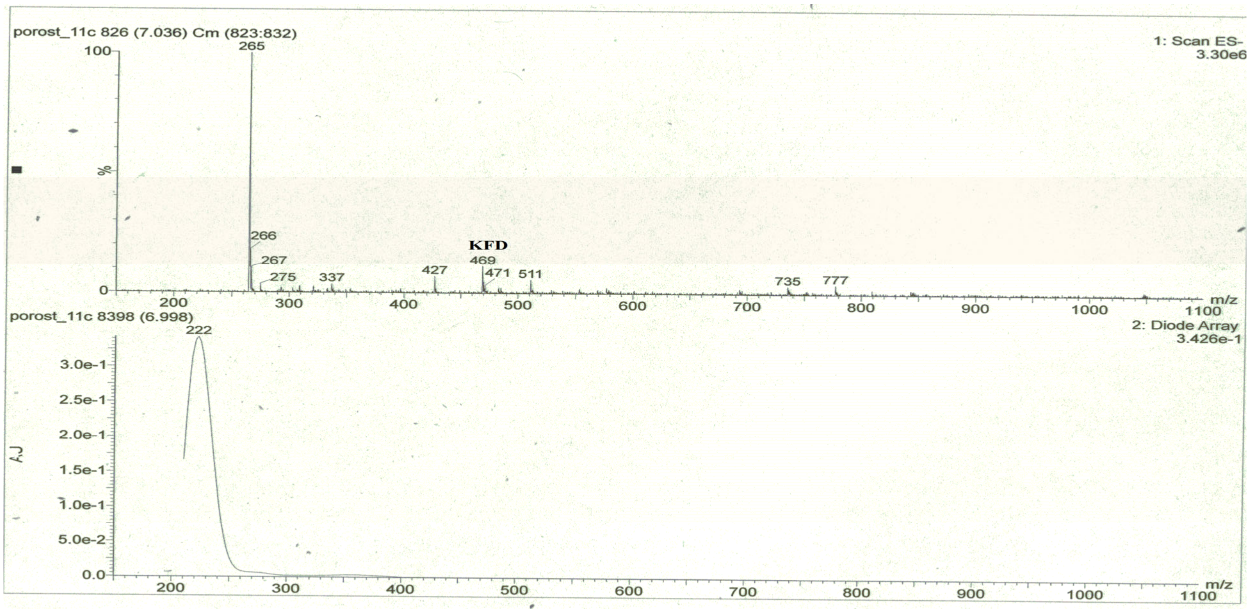
porost_11c 826 (7.036) Cm (823:832)
1: Scan ES-
3.30e6
100
%
0
265
266
267
275
337
427
KFD
469
471
511
735
777
m/z
200
300
400
500
600
700
800
900
1000
1100
porost_11c 8398 (6.998)
2: Diode Array
3.426e-1
222
3.0e-1
2.5e-1
2.0e-1
1.5e-1
1.0e-1
5.0e-2
0.0
AU

E

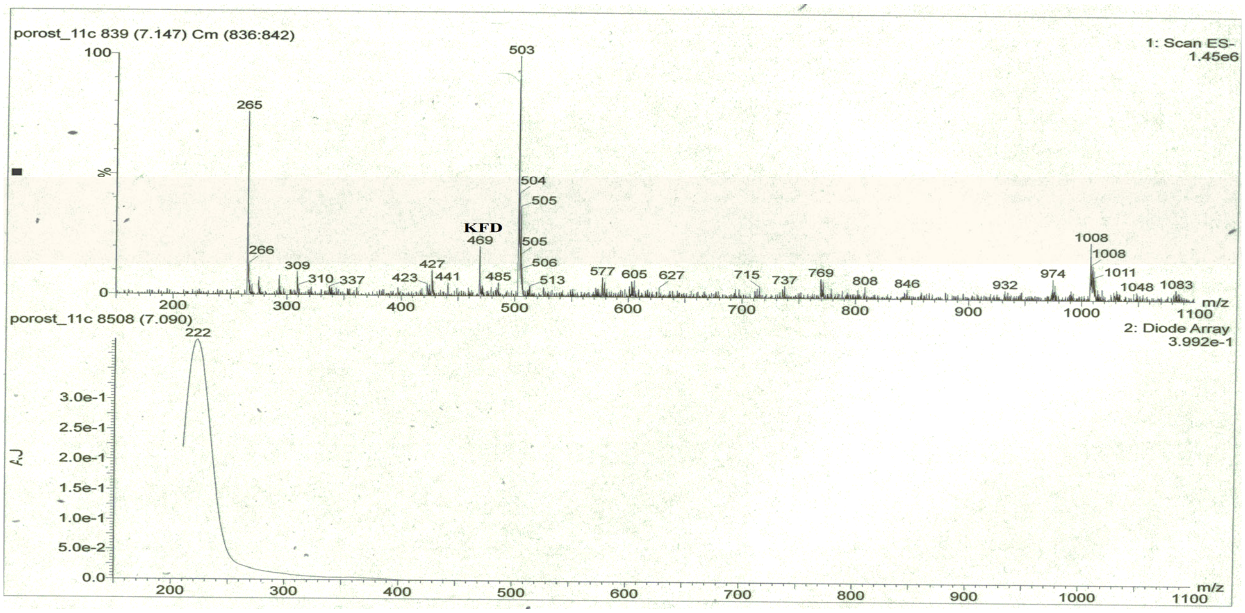
porost_11c 839 (7.147) Cm (836:842)
1: Scan ES-
1.45e6
503
265
KFD
469
1008
m/z
porost_11c 8508 (7.090)
222
2: Diode Array
3.992e-1
AU

F

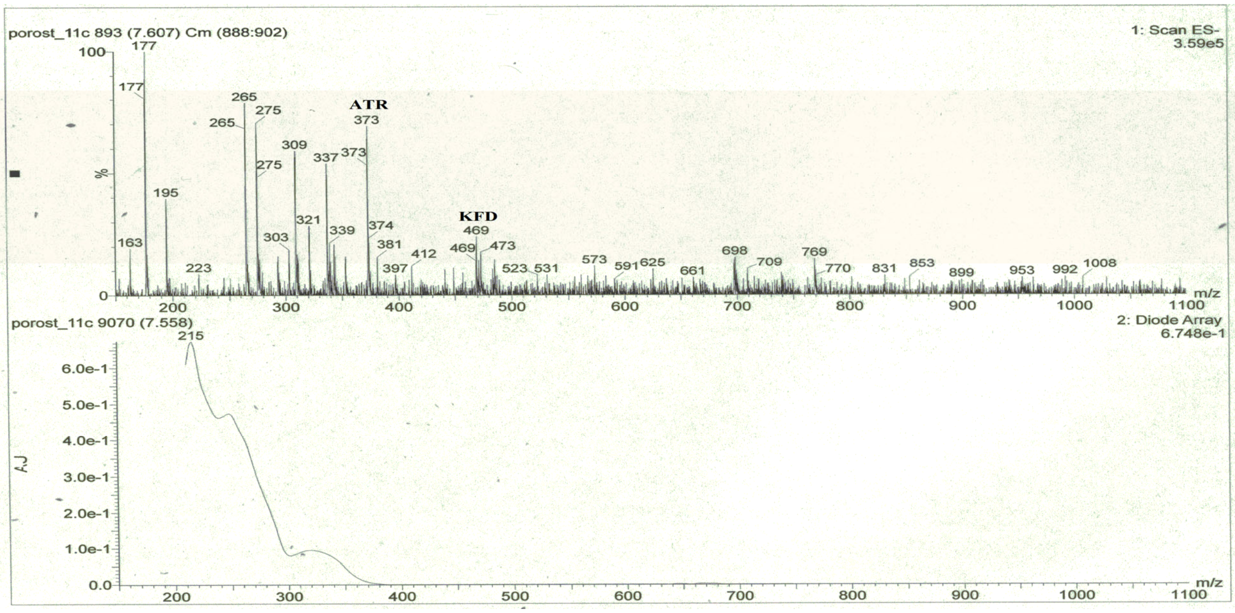
porost_11c 893 (7.607) Cm (888:902)
1: Scan ES-
3.59e5
177
265
275
ATR
373
309
337
KFD
469
698
769
m/z
porost_11c 9070 (7.558)
215
2: Diode Array
6.748e-1
AU

G

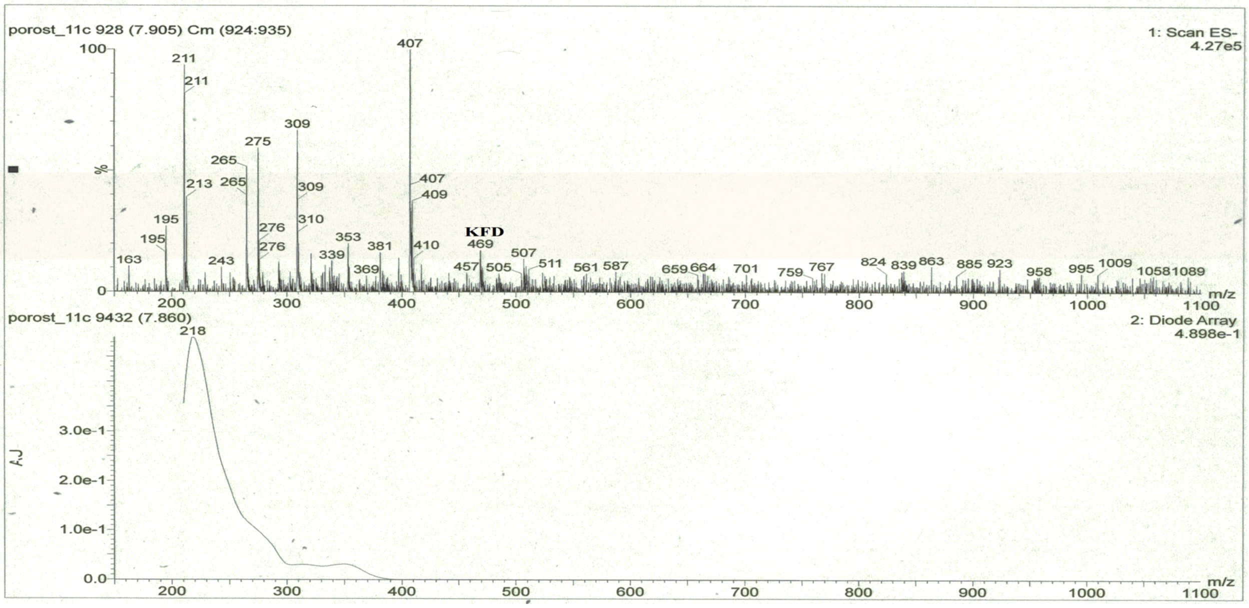
porost_11c 928 (7.905) Cm (924:935)
1: Scan ES-
4.27e5
%
KFD
m/z
porost_11c 9432 (7.860)
2: Diode Array
4.898e-1
AU
m/z
