# Supplementary material for: Antifungal and Profungal Potentials of Acetone-Extracted Complexes of Lichen Substances on Macromycete Heterobasidion parviporum Mycelium Growth Dynamics—The First In Vitro Approach
Source: Molecules. 2026 Aug 18;31(16):2882. doi: 10.3390/molecules31162882 (PMC13515570; doi:10.3390/molecules31162882)

Figure S9: Growth dynamics (mean colony diameter) of *Heterobasidion parviporum* mycelium at 28 °C in cultures treated with 1 and 2 mL of acetone or acetone extract from *Cladonia arbuscula* (N = 5 per treatment), with both treatments applied onto the surface of solidified Hansen's medium.

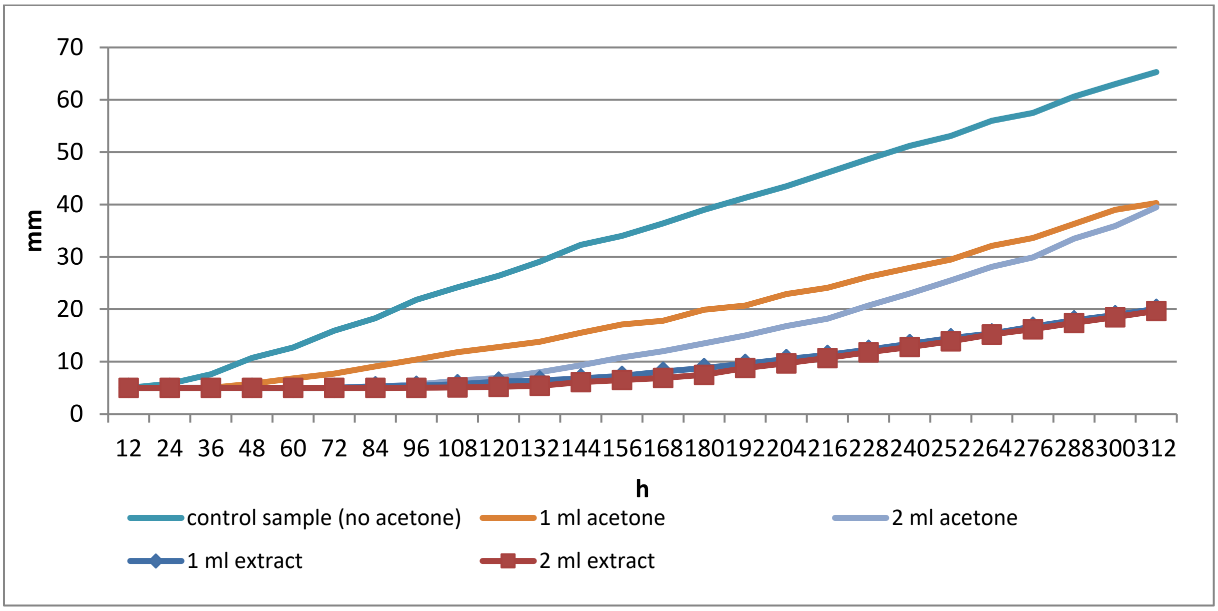

Figure S10: Growth dynamics (mean colony diameter) of *Heterobasidion parviporum* mycelium at 28 °C in cultures treated with 1–4 mL of acetone or acetone extract from *Cladonia arbuscula* (N = 5 per treatment), with both treatments incorporated into liquid Hansen's medium before solidification.

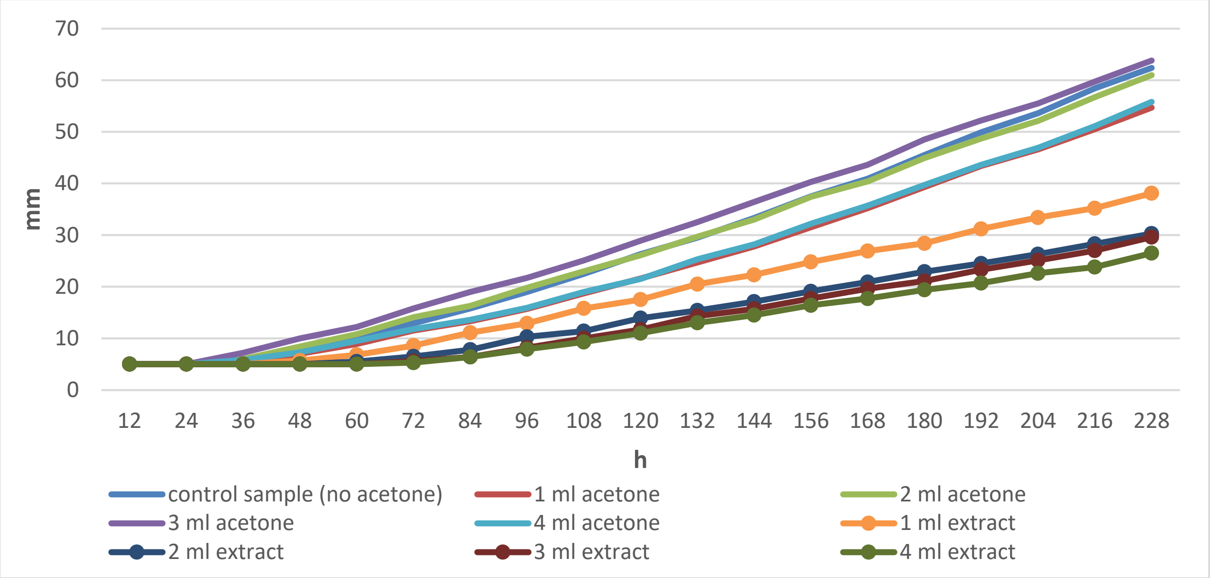

Figure S11: Growth dynamics (mean colony diameter) of *Heterobasidion parviporum* mycelium at 28 °C in cultures treated with 1 and 2 mL of acetone or acetone extract from *Cladonia rangiferina* (N = 5 per treatment), with both treatments applied onto the surface of solidified Hansen's medium.

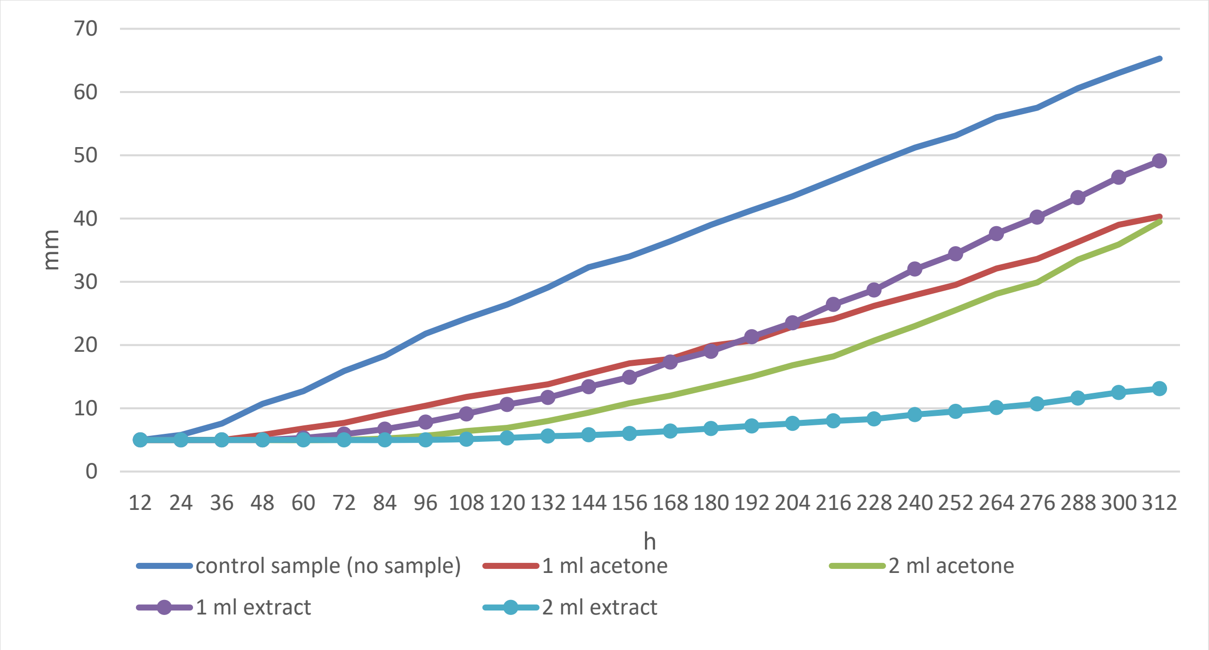

Figure S12: Growth dynamics (mean colony diameter) of *Heterobasidion parviporum* mycelium at 28 °C in cultures treated with 1–4 mL of acetone or acetone extract from *Cladonia rangiferina* (N = 5 per treatment), with both treatments incorporated into liquid Hansen's medium before solidification.

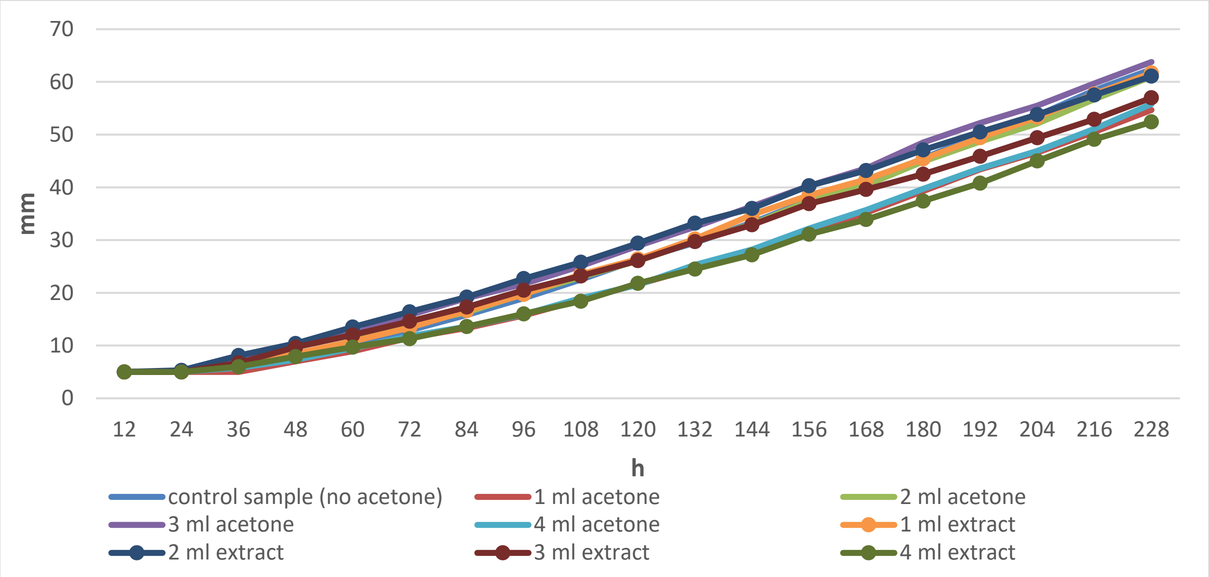

Figure S13: Growth dynamics (mean colony diameter) of *Heterobasidion parviporum* mycelium at 28 °C in cultures treated with 1–3 mL of acetone or acetone extract from *Cladonia digitata* (N = 5 per treatment), with both treatments applied onto the surface of solidified Hansen's medium.

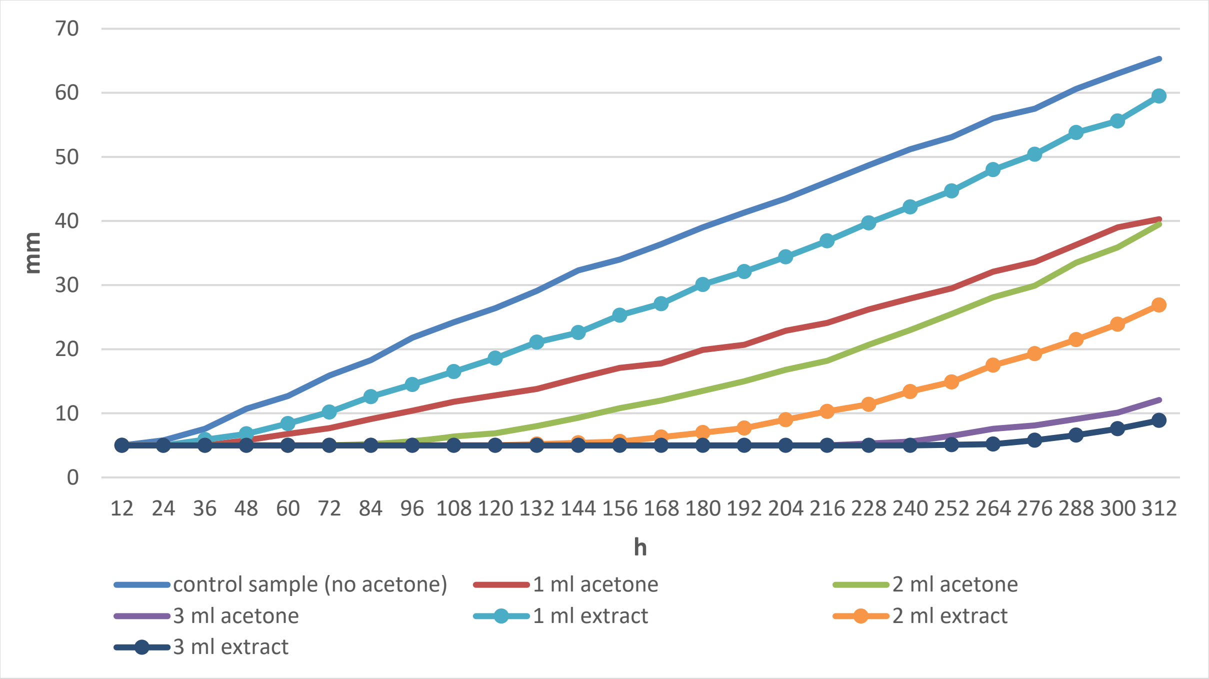

Figure S14: Growth dynamics (mean colony diameter) of *Heterobasidion parviporum* mycelium at 28 °C in cultures treated with 1–4 mL of acetone or acetone extract from *Cladonia digitata* (N = 5 per treatment), with both treatments incorporated into liquid Hansen's medium before solidification.

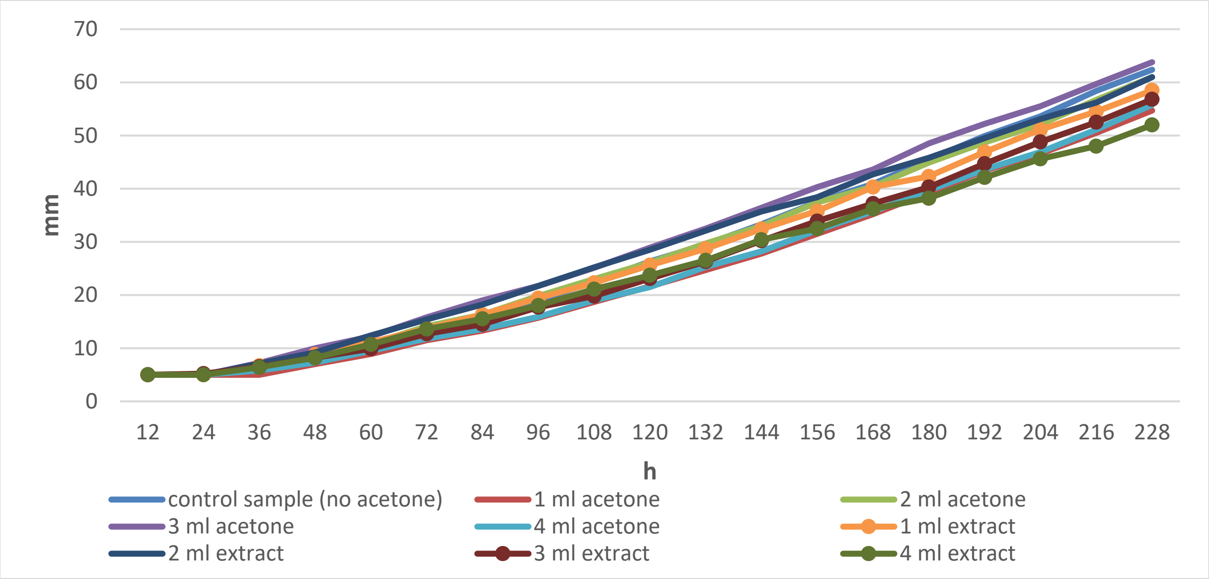

Figure S15: Growth dynamics (mean colony diameter) of *Heterobasidion parviporum* mycelium at 28 °C in cultures treated with 1–3 mL of acetone or acetone extract from *Cladonia uncialis* (N = 5 per treatment), with both treatments applied onto the surface of solidified Hansen's medium.

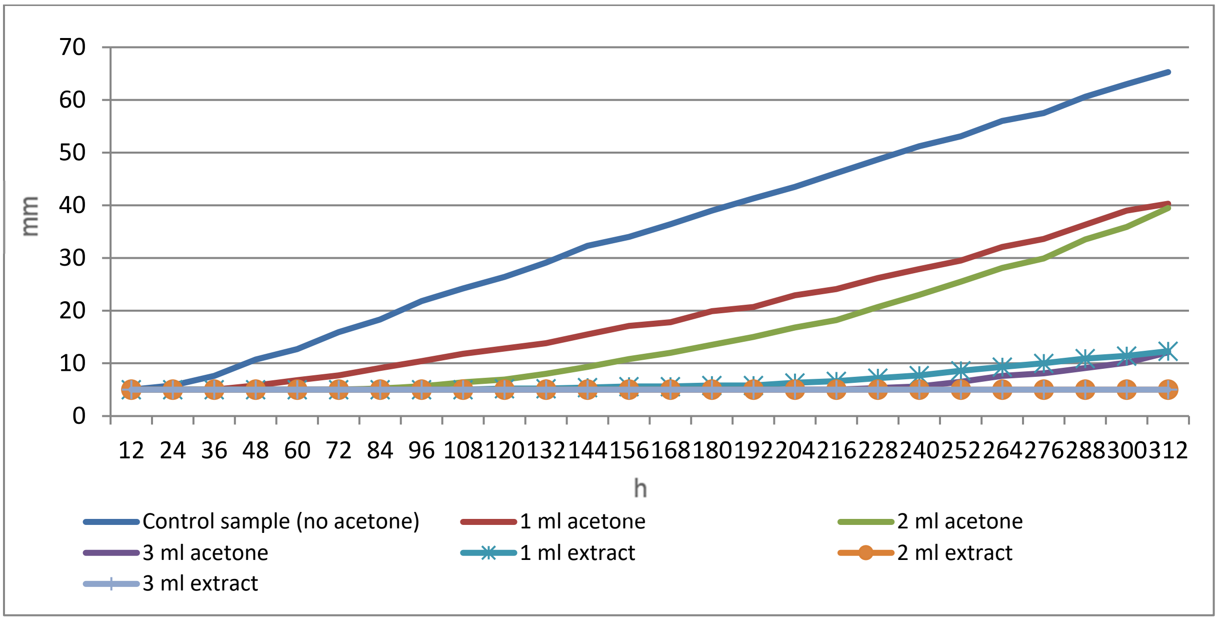

Figure S16: Growth dynamics (mean colony diameter) of *Heterobasidion parviporum* mycelium at 28 °C in cultures treated with 1–4 mL of acetone or acetone extract from *Cladonia uncialis* (N = 5 per treatment), with both treatments incorporated into liquid Hansen's medium before solidification.

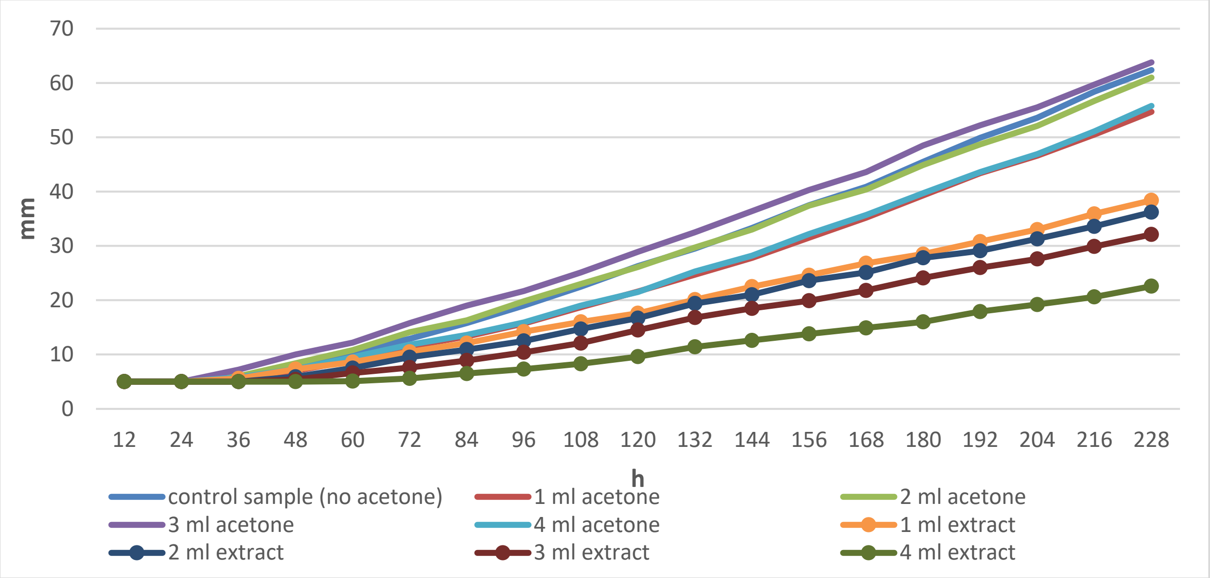

Figure S17: Growth dynamics (mean colony diameter) of *Heterobasidion parviporum* mycelium at 28 °C in cultures treated with 1–4 mL of acetone or acetone extract from *Cetraria islandica* (N = 5 per treatment), with both treatments incorporated into liquid Hansen's medium before solidification.

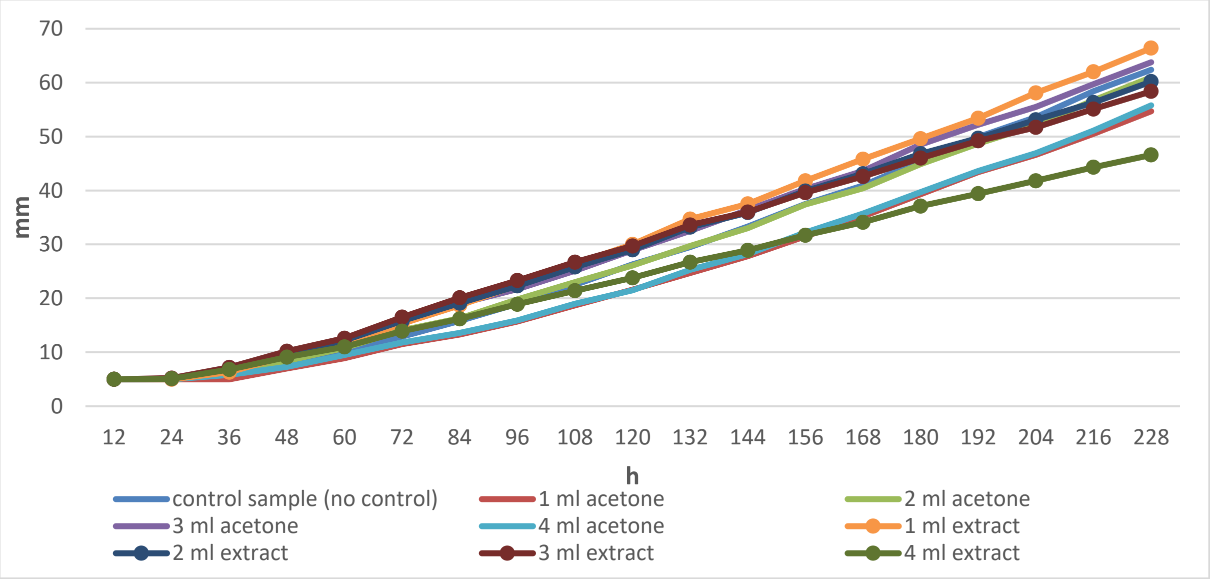

Figure S18: Growth dynamics (mean colony diameter) of *Heterobasidion parviporum* mycelium at 28 °C in cultures treated with 1–3 mL of acetone or acetone extract prepared from mixed thalli of *Cladonia arbuscula* + *Cladonia rangiferina* + *Cladonia digitata* (N = 5 per treatment), with both treatments applied onto the surface of solidified Hansen's medium.

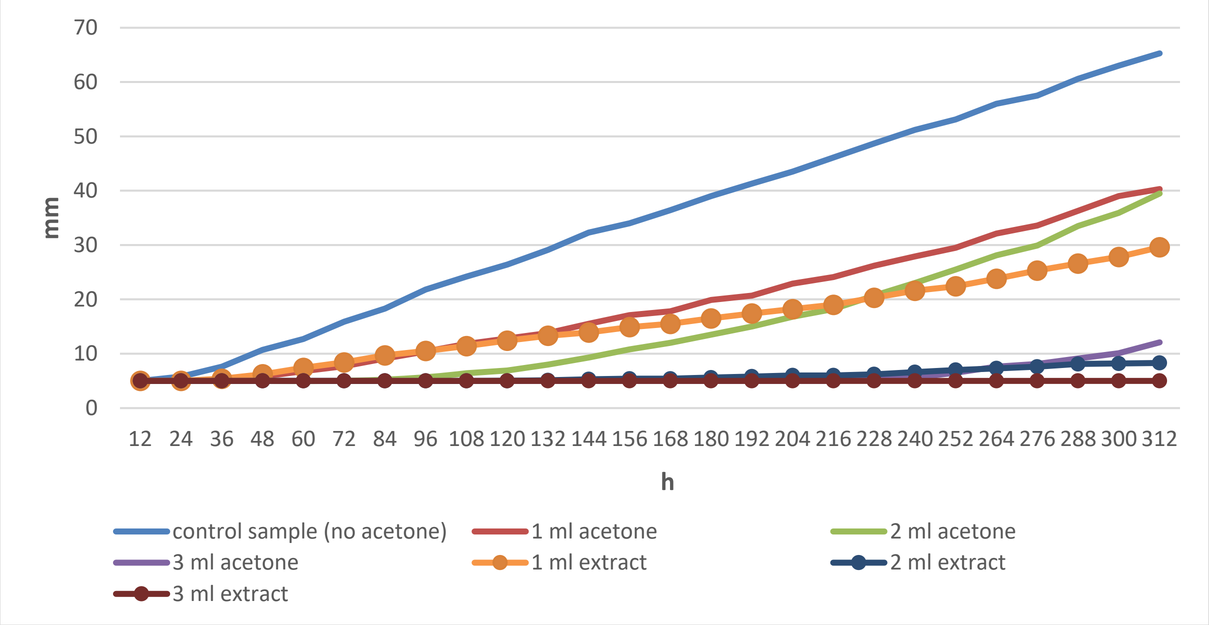

Figure S19: Growth dynamics (mean colony diameter) of *Heterobasidion parviporum* mycelium at 28 °C in cultures treated with 1–4 mL of acetone or acetone extract prepared from mixed thalli of *Cladonia arbuscula* + *Cladonia rangiferina* + *Cladonia digitata* (N = 5 per treatment), with both treatments incorporated into liquid Hansen's medium before solidification.

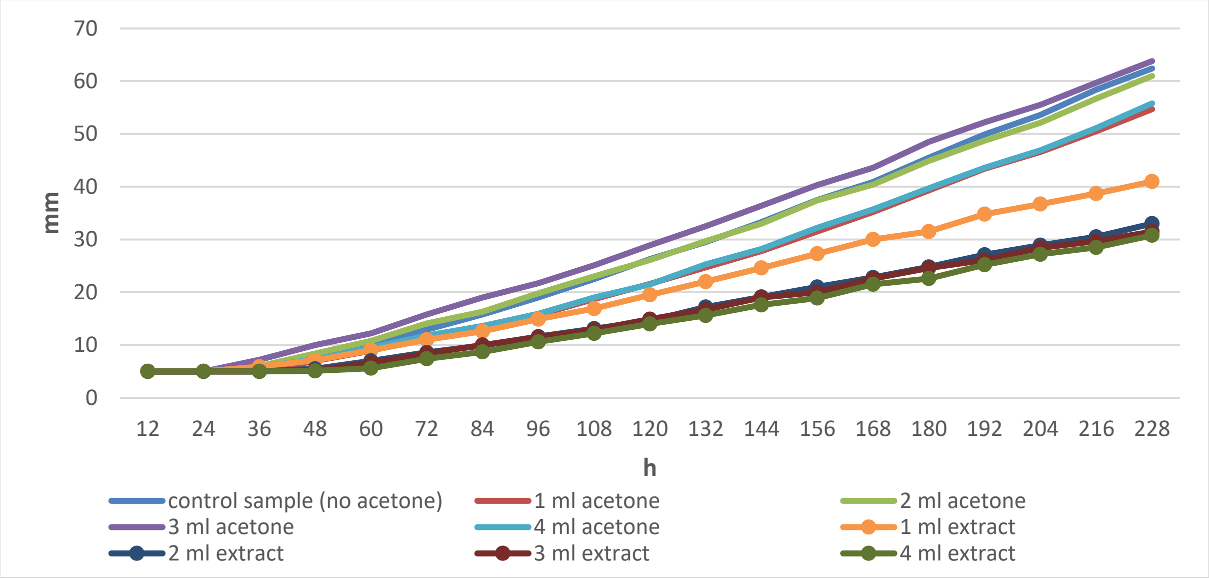

Figure S20: Growth dynamics (mean colony diameter) of *Heterobasidion parviporum* mycelium at 28 °C in cultures treated with 1–4 mL of acetone or acetone extract prepared from mixed thalli of *Cladonia arbuscula* + *Cladonia rangiferina* + *Cladonia digitata* + *Cladonia uncialis* + *Cetraria islandica* (N = 5 per treatment), with both treatments incorporated into liquid Hansen's medium before solidification.

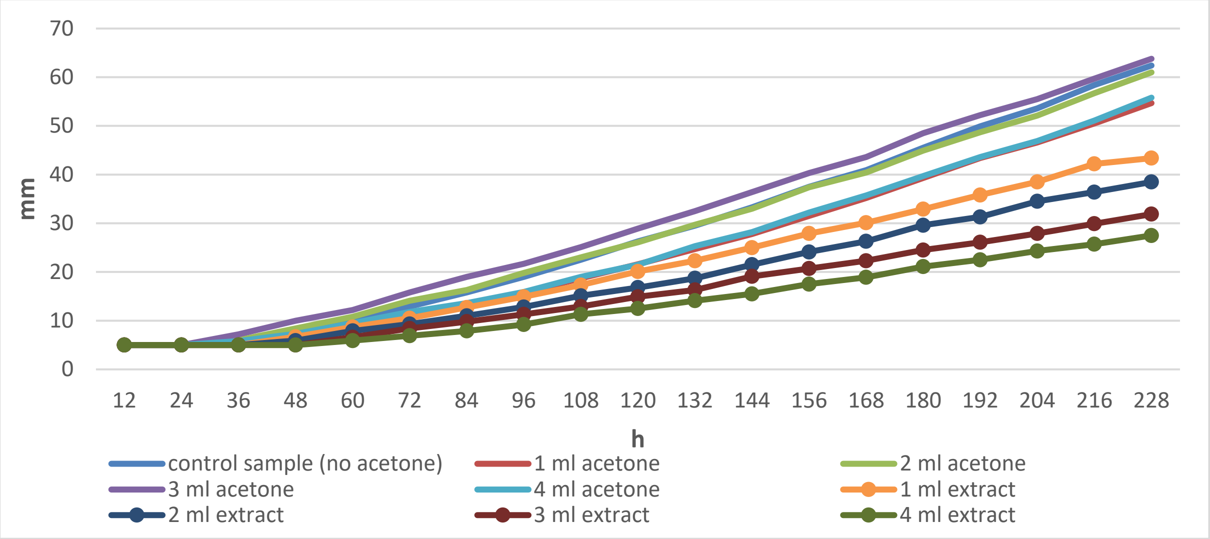

Figure S21: Growth dynamics (mean colony diameter) of *Heterobasidion parviporum* mycelium at 28 °C in cultures treated with 1–4 mL of acetone or acetone extract from *Pseudevernia furfuracea* (N = 5 per treatment), with both treatments incorporated into liquid Hansen's medium before solidification.

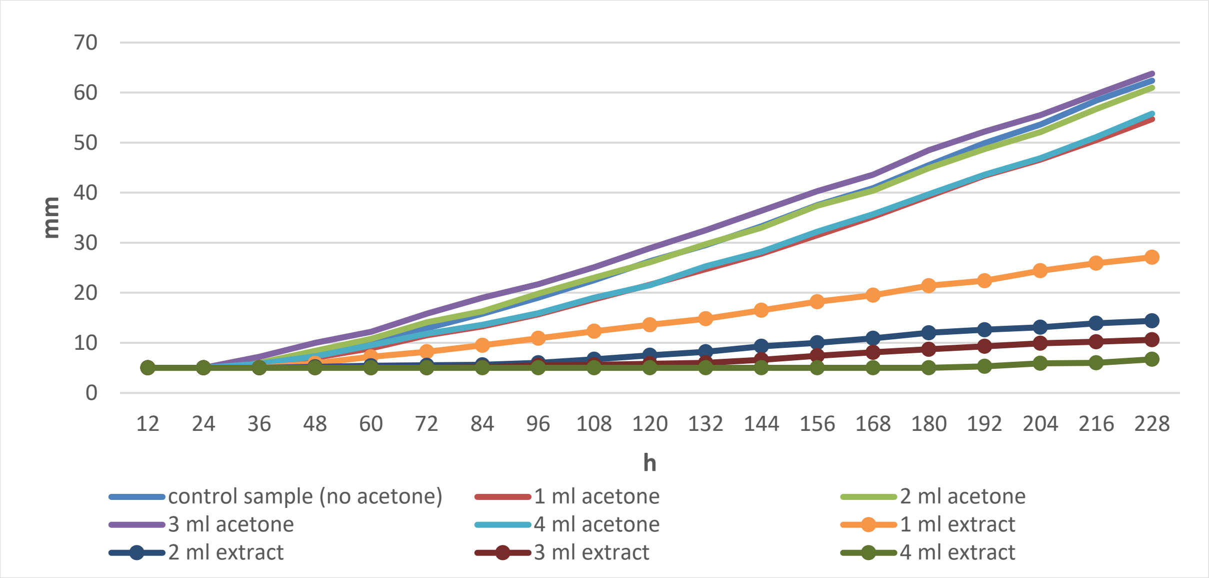

Supplement: Supplementary file 1 [file molecules-31-02882-s001.zip › Figures_S9-S21_Growth_dynamics.pdf]
