# Supplementary material for: Antifungal and Profungal Potentials of Acetone-Extracted Complexes of Lichen Substances on Macromycete Heterobasidion parviporum Mycelium Growth Dynamics—The First In Vitro Approach
Source: Molecules. 2026 Aug 18;31(16):2882. doi: 10.3390/molecules31162882 (PMC13515570; doi:10.3390/molecules31162882)
Supplement: Supplementary file 1 [file molecules-31-02882-s001.zip › Table_S1_Hansen_medium.pdf]

Table S1: Composition of Hansen's medium used to cultivate *Heterobasidion parviporum* mycelium (quantities per 1 L of distilled water).

**Hansen's medium**
2.5 g glucose
2.5 g maltose
1 g bacteriological peptone
0.2 g $MgSO_4$
0.3 g KH_2PO_4
30 g agar
