# Supplementary material for: Antifungal and Profungal Potentials of Acetone-Extracted Complexes of Lichen Substances on Macromycete Heterobasidion parviporum Mycelium Growth Dynamics—The First In Vitro Approach
Source: Molecules. 2026 Aug 18;31(16):2882. doi: 10.3390/molecules31162882 (PMC13515570; doi:10.3390/molecules31162882)
Supplement: Supplementary file 1 [file molecules-31-02882-s001.zip › Table_S20_Surface_extract_vs_acetone.pdf]

Table S20: Comparison of *Heterobasidion parviporum* mycelial growth dynamics between cultures treated with eight lichen acetone-extract variants and cultures receiving equivalent acetone volumes (1–3 mL), applied onto the surface of solidified Hansen's medium, incubated at 28 °C, and measured at 12 h intervals.

a, b, c, d, e, f, g, h - statistically significant differences between the compared pairs of fungal cultures supplemented with the appropriate dose of lichen extract.
"-" - no measurement or inability to adequately compare the mean diameter of the mycelium in samples with extract to the mean diameter of the mycelium in samples with the dose of acetone at a given measurement time.

Heterobasidion parviporum																											
	Average mycelium diameter [mm] at a given measurement time [h] ± standard deviation from sample [mm]																										
	12	24	36	48	60	72	84	96	108	120	132	144	156	168	180	192	204	216	228	240	252	264	276	288	300	312
**1 mL acetone**	**5 ±0**	**5 ±0**	**5 ±0**	**5,8 ±0,27**	**6,8 ±0,45**	**7,7 ±0,67**	**9,1 ±0,82**	**10,4 ±0,65**	**11,8 ±1,04**	**12,8 ±1,48**	**13,8 ±1,64**	**15,5 ±1,87**	**17,1 ±1,75**	**17,8 ±1,89**	**19,9 ±1,98**	**20,7 ±2,11**	**22,9 ±2,01**	**24,1 ±2,38**	**26,2 ±1,64**	**27,9 ±2,01**	**29,5 ±1,87**	**32,1 ±2,19**	**33,6 ±1,35**	**36,3 ±1,35**	**39 ±1,94**	**40,3 ±1,75**
1 mL *Cladonia arbuscula*	5 ±0	5 ±0	5 ±0	5 ±0	5 ±0	5[a] ±0	5,3 ±0,45	5,4 ±0,55	5,7 ±0,97	6,2 ±1,64	6,4 ±1,95	6,8 ±2,25	7,3 ±2,73	8,1 ±3,29	8,8 ±3,77	9,6 ±4,51	10,5 ±5,15	11,3 ±5,43	12,3 ±5,74	13,4 ±6,32	14,5 ±6,91	15,4 ±7,29	16,7 ±8,12	17,9 ±8,51	18,9 ±9,04	20,1 ±9,63
1 mL *C. rangiferina*	5 ±0	5 ±0	5 ±0	5 ±0	5,3 ±0,45	5,9 ±0,89	6,7 ±1,35	7,8 ±1,04	9,1 ±1,29	10,6 ±1,29	11,7 ±1,4	13,4 ±1,71	14,9 ±1,98	17,3 ±2,2	19 ±2,47	21,3 ±2,61	23,5 ±2,78	26,4 ±3,03	28,7 ±3,51	32 ±3,45	34,4 ±4,02	37,6 ±4,38	40,2 ±4,56	43,3[b, c, d] ±4,79	46,5[b, c, d] ±5,04	49,1[b, c, d] ±4,84
1 mL *C. digitata*	5 ±0	5 ±0	5,9 ±0,22	6,8 ±0,84	8,4 ±1,08	10,2[a, e, f, g, h] ±1,2	12,6[e, f, g, h] ±1,14	14,5[e, f, g, h] ±1,22	16,5[e, f, g, h] ±1,32	18,6[e, f, g, h] ±1,47	21,1[e, f, g, h] ±1,29	22,6[e, f, g, h] ±1,64	25,3[e, f, g, h] ±1,86	27,1[e, f, g, h] ±1,64	30,1[e, f, g, h] ±2,07	32,1[e, f, g, h] ±2,07	34,4[e, f, g, h] ±2,16	36,9[e, f, g, h] ±2,3	39,7[e, f, g, h] ±2,64	42,2[e, f, g, h] ±2,02	44,7[e, f, g, h] ±2,97	48[e, f, g, h] ±3	50,4[e, f, g, h] ±2,79	53,8[e, f, g, h] ±3,49	55,6[e, f, g, h] ±3,47	59,5[f, g, h] ±3,45
1 mL *C. uncialis*	5 ±0	5 ±0	5 ±0	5 ±0	5 ±0	5[e] ±0	5[e] ±0	5[e] ±0	5[e] ±0	5,2[e] ±0,45	5,2[e] ±0,45	5,4[e] ±0,89	5,6[e] ±1,34	5,6[e] ±1,34	5,8[e] ±1,79	5,8[e] ±1,79	6,3[e] ±2,64	6,6[e] ±3,05	7,2[e] ±4,13	7,7[e] ±4,76	8,6[e] ±6,07	9,3[e] ±6,96	10[e] ±7,71	10,9[e] ±8,89	11,4[e] ±9,74	12,3 ±10,79
1 mL *Cetraria islandica*	5 ±0	5 ±0	5 ±0	5 ±0	5 ±0	5[f] ±0	5[f] ±0	5[f] ±0	5[f] ±0	5[f] ±0	5[f] ±0	5[f] ±0	5[f] ±0	5[f] ±0	5[f] ±0	5[f] ±0	5[f] ±0	5[f] ±0	5[f] ±0	5[f] ±0	5[f] ±0	5[f] ±0	5[f] ±0	5[b, f] ±0	5[b, f] ±0	5[b, f] ±0
1 mL *Pseudevernia furfuracea*	5 ±0	5 ±0	5 ±0	5 ±0	5 ±0	5[g] ±0	5[g] ±0	5[g] ±0	5[g] ±0	5[g] ±0	5[g] ±0	5[g] ±0	5[g] ±0	5[g] ±0	5[g] ±0	5[g] ±0	5[g] ±0	5[g] ±0	5[g] ±0	5[g] ±0	5[g] ±0	5[g] ±0	5[g] ±0	5[c, g] ±0	5[c, g] ±0	5[c, g] ±0
1 mL *C. arbuscula* + *C. rangiferina* + *C. digitata*	5 ±0	5 ±0	5,4 ±0,55	6,2 ±1,44	7,4 ±1,98	8,4 ±2,41	9,7 ±2,59	10,5 ±3,67	11,4 ±4,29	12,4 ±4,89	13,3 ±5,69	13,9 ±6,07	14,9 ±7,22	15,5 ±8,07	16,5 ±8,76	17,4 ±9,48	18,2 ±10,37	19 ±11,25	20,3 ±12,3	21,6 ±13,18	22,4 ±13,81	23,8 ±15,28	25,3 ±15,96	26,6 ±16,82	27,8 ±17,54	29,6 ±19,12
1 mL *C. arbuscula* + *C. rangiferina* + *C. digitata* + *C. uncialis* + *C. islandica*	5 ±0	5 ±0	5 ±0	5 ±0	5 ±0	5[h] ±0	5[h] ±0	5[h] ±0	5[h] ±0	5[h] ±0	5[h] ±0	5[h] ±0	5[h] ±0	5[h] ±0	5[h] ±0	5[h] ±0	5[h] ±0	5[h] ±0	5[h] ±0	5[h] ±0	5[h] ±0	5[h] ±0	5[h] ±0	5[d, h] ±0	5[d, h] ±0	5[d, h] ±0
**2 mL acetone**	**5 ±0**	**5 ±0**	**5 ±0**	**5 ±0**	**5 ±0**	**5 ±0**	**5,2 ±0,45**	**5,7 ±0,84**	**6,4 ±1,39**	**6,9 ±1,98**	**8 ±2,37**	**9,3 ±2,59**	**10,8 ±3,37**	**12 ±3,48**	**13,5 ±4,23**	**15 ±4,3**	**16,8 ±4,83**	**18,2 ±5,39**	**20,7 ±5,43**	**23 ±6,19**	**25,5 ±6,97**	**28,1 ±6,98**	**29,9 ±7,82**	**33,5 ±8,14**	**35,9 ±9,75**	**39,5 ±9,75**
2 mL *Cladonia arbuscula*	5 ±0	5 ±0	5 ±0	5 ±0	5 ±0	5 ±0	5 ±0	5 ±0	5,1 ±0,22	5,2 ±0,45	5,4 ±0,65	6,1 ±0,74	6,5 ±1,12	6,9 ±1,29	7,5 ±1,58	8,8 ±1,35	9,7 ±1,2	10,7 ±1,35	11,8 ±1,57	12,8 ±1,79	13,9 ±2,33	15,2 ±1,82	16,2 ±1,82	17,4 ±2,01	18,5 ±2,35	19,7 ±2,08
2 mL *C. rangiferina*	5 ±0	5 ±0	5 ±0	5 ±0	5 ±0	5 ±0	5 ±0	5 ±0	5,1 ±0,22	5,3 ±0,67	5,6 ±1,34	5,8 ±1,79	6 ±2,24	6,4 ±3,13	6,8 ±4,02	7,2 ±4,92	7,6 ±5,81	8 ±6,71	8,3 ±7,38	9 ±8,94	9,5 ±10,06	10,1 ±11,4	10,7 ±12,19	11,6 ±13,67	12,5 ±15,15	13,1 ±16,23
2 mL *C. digitata*	5 ±0	5 ±0	5 ±0	5 ±0	5 ±0	5 ±0	5 ±0	5 ±0	5 ±0	5 ±0	5,2 ±0,45	5,4 ±0,89	5,6 ±0,89	6,3 ±1,89	7 ±2,42	7,7 ±2,89	9 ±3,59	10,3 ±4,22	11,4 ±4,55	13,4 ±5,58	14,9 ±6,12	17,5 ±7,27	19,3 ±7,64	21,5 ±8,33	23,9 ±9,4	26,9 ±10,44
2 mL *C. uncialis*	5 ±0	5 ±0	5 ±0	5 ±0	5 ±0	5 ±0	5 ±0	5 ±0	5 ±0	5 ±0	5 ±0	5 ±0	5 ±0	5 ±0	5 ±0	5 ±0	5 ±0	5 ±0	5 ±0	5 ±0	5 ±0	5 ±0	5 ±0	5 ±0	5 ±0	5 ±0
2 mL *Cetraria islandica*	5 ±0	5 ±0	5 ±0	5 ±0	5 ±0	5 ±0	5 ±0	5 ±0	5 ±0	5 ±0	5 ±0	5 ±0	5 ±0	5 ±0	5 ±0	5 ±0	5 ±0	5 ±0	5 ±0	5 ±0	5 ±0	5 ±0	5 ±0	5 ±0	5 ±0	5 ±0

2 mL *Pseudevernia furfuracea*	5 ±0	5 ±0	5 ±0	5 ±0	5 ±0	5 ±0	5 ±0	5 ±0	5 ±0	5 ±0	5 ±0	5 ±0	5 ±0	5 ±0	5 ±0	5 ±0	5 ±0	5 ±0	5 ±0	5 ±0	5 ±0	5 ±0	5 ±0	5 ±0	5 ±0	5 ±0
2 mL *C. arbuscula* + *C. rangiferina* + *C. digitata*	5 ±0	5 ±0	5 ±0	5 ±0	5 ±0	5 ±0	5 ±0	5 ±0	5 ±0	5 ±0	5,1 ±0,22	5,3 ±0,67	5,4 ±0,89	5,4 ±0,89	5,6 ±1,34	5,8 ±1,3	6 ±1,54	6 ±1,54	6,2 ±1,68	6,6 ±1,78	7 ±1,77	7,3 ±2,08	7,6 ±1,98	8,1 ±2,3	8,2 ±2,33	8,3 ±2,36
2 mL *C. arbuscula* + *C. rangiferina* + *C. digitata* + *C. uncialis* + *C. islandica*	5 ±0	5 ±0	5 ±0	5 ±0	5 ±0	5 ±0	5 ±0	5 ±0	5 ±0	5 ±0	5 ±0	5 ±0	5 ±0	5 ±0	5 ±0	5 ±0	5 ±0	5 ±0	5 ±0	5 ±0	5 ±0	5 ±0	5 ±0	5 ±0	5 ±0	5 ±0
**3 mL acetone**	**5 ±0**	**5 ±0**	**5 ±0**	**5 ±0**	**5 ±0**	**5 ±0**	**5 ±0**	**5 ±0**	**5 ±0**	**5 ±0**	**5 ±0**	**5 ±0**	**5 ±0**	**5 ±0**	**5 ±0**	**5 ±0**	**5 ±0**	**5 ±0**	**5,3 ±0,45**	**5,6 ±0,89**	**6,5 ±2,06**	**7,6 ±2,79**	**8,1 ±3,29**	**9,1 ±4,02**	**10,1 ±5,02**	**12,1 ±6,5**
3 mL *Cladonia arbuscula*	-	-	-	-	-	-	-	-	-	-	-	-	-	-	-	-	-	-	-	-	-	-	-	-	-	-
3 mL *C. rangiferina*	-	-	-	-	-	-	-	-	-	-	-	-	-	-	-	-	-	-	-	-	-	-	-	-	-	-
3 mL *C. digitata*	5 ±0	5 ±0	5 ±0	5 ±0	5 ±0	5 ±0	5 ±0	5 ±0	5 ±0	5 ±0	5 ±0	5 ±0	5 ±0	5 ±0	5 ±0	5 ±0	5 ±0	5 ±0	5 ±0	5 ±0	5,1 ±0,22	5,2 ±0,45	5,8 ±1,25	6,6 ±2,04	7,6 ±2,72	8,9 ±4,16
3 mL *C. uncialis*	5 ±0	5 ±0	5 ±0	5 ±0	5 ±0	5 ±0	5 ±0	5 ±0	5 ±0	5 ±0	5 ±0	5 ±0	5 ±0	5 ±0	5 ±0	5 ±0	5 ±0	5 ±0	5 ±0	5 ±0	5 ±0	5 ±0	5 ±0	5 ±0	5 ±0	5 ±0
3 mL *Cetraria islandica*	5 ±0	5 ±0	5 ±0	5 ±0	5 ±0	5 ±0	5 ±0	5 ±0	5 ±0	5 ±0	5 ±0	5 ±0	5 ±0	5 ±0	5 ±0	5 ±0	5 ±0	5 ±0	5 ±0	5 ±0	5 ±0	5 ±0	5 ±0	5 ±0	5 ±0	5 ±0
3 mL *Pseudevernia furfuracea*	5 ±0	5 ±0	5 ±0	5 ±0	5 ±0	5 ±0	5 ±0	5 ±0	5 ±0	5 ±0	5 ±0	5 ±0	5 ±0	5 ±0	5 ±0	5 ±0	5 ±0	5 ±0	5 ±0	5 ±0	5 ±0	5 ±0	5 ±0	5 ±0	5 ±0	5 ±0
3 mL *C. arbuscula* + *C. rangiferina* + *C. digitata*	5 ±0	5 ±0	5 ±0	5 ±0	5 ±0	5 ±0	5 ±0	5 ±0	5 ±0	5 ±0	5 ±0	5 ±0	5 ±0	5 ±0	5 ±0	5 ±0	5 ±0	5 ±0	5 ±0	5 ±0	5 ±0	5 ±0	5 ±0	5 ±0	5 ±0	5 ±0
3 mL *C. arbuscula* + *C. rangiferina* + *C. digitata* + *C. uncialis* + *C. islandica*	5 ±0	5 ±0	5 ±0	5 ±0	5 ±0	5 ±0	5 ±0	5 ±0	5 ±0	5 ±0	5 ±0	5 ±0	5 ±0	5 ±0	5 ±0	5 ±0	5 ±0	5 ±0	5 ±0	5 ±0	5 ±0	5 ±0	5 ±0	5 ±0	5 ±0	5 ±0
