# Supplementary material for: Antifungal and Profungal Potentials of Acetone-Extracted Complexes of Lichen Substances on Macromycete Heterobasidion parviporum Mycelium Growth Dynamics—The First In Vitro Approach
Source: Molecules. 2026 Aug 18;31(16):2882. doi: 10.3390/molecules31162882 (PMC13515570; doi:10.3390/molecules31162882)
Supplement: Supplementary file 1 [file molecules-31-02882-s001.zip › Table_S21_Medium_extract_vs_acetone.pdf]

**Table S21:** Comparison of *Heterobasidion parviporum* mycelial growth dynamics between cultures treated with eight lichen acetone-extract variants and cultures receiving equivalent acetone volumes (1–4 mL), incorporated into liquid Hansen's medium before solidification, incubated at 28 °C, and measured at 12 h intervals.

a, b, c, d, e, f, g, h, i, j - statistically significant differences between compared pairs of fungal cultures in samples supplemented with the appropriate dose of lichen extract.

| ***Heterobasidion parviporum*** | | | | | | | | | | | | | | | | | | | |
|---|---|---|---|---|---|---|---|---|---|---|---|---|---|---|---|---|---|---|---|
| | Average mycelium diameter [mm] at a given measurement time [h] ±standard deviation in sample [mm] | | | | | | | | | | | | | | | | | | |
| | 12 | 24 | 36 | 48 | 60 | 72 | 84 | 96 | 108 | 120 | 132 | 144 | 156 | 168 | 180 | 192 | 204 | 216 | 228 |
| **1 mL acetone** | **5 ±0** | **5 ±0** | **5 ±0** | **7 ±0,94** | **8,9 ±1,02** | **11,5 ±0,87** | **13,3 ±1,6** | **15,7 ±2,2** | **18,7 ±2,59** | **21,6 ±3,09** | **24,7 ±3,78** | **27,8 ±4,09** | **31,5 ±4,68** | **35,2 ±4,83** | **39,3 ±5,51** | **43,4 ±6,46** | **46,6 ±6,09** | **50,5 ±6,05** | **54,7 ±5,85** |
| 1 mL *Cladonia arbuscula* | 5 ±0 | 5 ±0 | 5 ±0 | 5,7[b, c] ±0,67 | 6,8[a, b, c] ±1,04 | 8,6[a, b, c] ±1,19 | 11,1[c] ±1,43 | 12,9[c] ±1,34 | 15,8[c] ±2,33 | 17,5[c] ±2,81 | 20,5[c] ±2,96 | 22,3[c] ±3,91 | 24,8[c] ±4,86 | 26,9[c] ±6,07 | 28,4[c] ±7 | 31,2[c] ±7,46 | 33,4[c] ±7,85 | 35,2[c] ±7,83 | 38,1[c] ±8,88 |
| 1 mL *C. rangiferina* | 5 ±0 | 5 ±0 | 6,4 ±0,55 | 8,5[b] ±0,61 | 10,8[a, b, d] ±0,45 | 13,4[a, b, d] ±0,82 | 16,6[d] ±0,82 | 19,7[d] ±1,35 | 23,5[d] ±1,12 | 26,4[d] ±1,29 | 30,2[d] ±1,68 | 34,8[d] ±1,3 | 38,5[d] ±2,03 | 41,5[d] ±1,66 | 45,4[d] ±1,64 | 49,4[d] ±1,64 | 53,3[d] ±1,6 | 57,7[d] ±1,6 | 61,7[d] ±1,15 |
| 1 mL *C. digitata* | 5 ±0 | 5 ±0 | 6,7 ±0,67 | 8,8 ±1,44 | 11,1[e] ±1,56 | 13,7[e] ±1,64 | 16,3[e] ±2,08 | 19,4[e] ±2,95 | 22,3[e] ±3,38 | 25,6[e] ±4,17 | 28,7[e] ±4,55 | 32,4[e] ±5,28 | 35,8[e] ±5,84 | 40,3[e] ±6,05 | 42,3[e] ±6,26 | 46,9[e] ±7,12 | 51,1[e] ±7,44 | 54,5[e] ±7,88 | 58,5[e] ±8,76 |
| 1 mL *C. uncialis* | 5 ±0 | 5 ±0 | 5,6 ±0,55 | 7,3 ±1,68 | 8,6 ±2,33 | 10,5 ±3,32 | 12,1 ±4,16 | 14,2 ±5,39 | 16 ±6,32 | 17,6 ±7,22 | 20,1 ±8,73 | 22,5 ±9,94 | 24,6 ±11,22 | 26,8 ±12,34 | 28,5 ±13,22 | 30,8 ±14,71 | 33 ±15,95 | 35,9 ±17,71 | 38,4 ±19,11 |
| 1 mL *Cetraria islandica* | 5 ±0 | 5 ±0 | 6,3 ±0,67 | 9,3[c, f] ±1,4 | 11,4[c, f] ±2,04 | 15,3[c, f] ±2,97 | 18,7[c, f] ±3,68 | 22,4[c, f] ±4,11 | 26,4[c, f] ±4,35 | 30[c, f] ±4,81 | 34,7[c, f] ±5,31 | 37,5[c, f] ±4,96 | 41,8[c, f] ±5,03 | 45,8[c, f] ±5,19 | 49,6[c, f, g] ±4,79 | 53,4[c, f] ±5,76 | 58,1[c, f, g] ±5,27 | 62[c, f, g] ±5,44 | 66,4[c, f, g] ±4,84 |
| 1 mL *Pseudevernia furfuracea* | 5 ±0 | 5 ±0 | 5 ±0 | 5,9[f] ±0,74 | 7,2[d, e, f] ±0,57 | 8,2[d, e, f] ±1,3 | 9,5[d, e, f] ±1,5 | 10,9[d, e, f] ±2,04 | 12,3[d, e, f] ±2,39 | 13,6[d, e, f] ±2,9 | 14,8[d, e, f] ±3,13 | 16,5[d, e, f] ±3,16 | 18,2[d, e, f] ±3,38 | 19,5[d, e, f] ±3,61 | 21,4[d, e, f] ±3,75 | 22,4[d, e, f] ±3,75 | 24,4[d, e, f] ±3,9 | 25,9[d, e, f] ±4,02 | 27,1[d, e, f] ±4,32 |
| 1 mL *C. arbuscula* + *C. rangiferina* + *C. digitata* | 5 ±0 | 5 ±0 | 5,9 ±0,22 | 7,1 ±0,74 | 9 ±0,35 | 11 ±0,71 | 12,6 ±1,24 | 14,9 ±1,67 | 16,9 ±1,24 | 19,5 ±1,58 | 22 ±1,22 | 24,6 ±1,08 | 27,3 ±0,91 | 30 ±1,77 | 31,5[g] ±0,79 | 34,8 ±1,04 | 36,7[g] ±0,76 | 38,7[g] ±0,97 | 41[g] ±0,61 |
| 1 mL *C. arbuscula* + *C. rangiferina* + *C. digitata* + *C. uncialis* + *C. islandica* | 5 ±0 | 5 ±0 | 5 ±0 | 6,7 ±0,45 | 8,7 ±0,45 | 10,5 ±0,5 | 12,7 ±0,45 | 14,9 ±0,42 | 17,3 ±0,57 | 20,1 ±0,42 | 22,3 ±1,2 | 25 ±0,79 | 27,9 ±0,96 | 30,1 ±0,74 | 32,9 ±1,56 | 35,8 ±1,75 | 38,5 ±2,06 | 42,2 ±2,59 | 43,4 ±2,16 |
| **2 mL acetone** | **5 ±0** | **5 ±0** | **6 ±0,35** | **8,4 ±0,65** | **10,8 ±0,57** | **14,1 ±0,55** | **16,3 ±0,45** | **19,8 ±1,25** | **23 ±1,66** | **26,1 ±1,14** | **29,7 ±1,35** | **33 ±1,58** | **37,4 ±2,04** | **40,4 ±1,78** | **44,9 ±2,51** | **48,7 ±2,36** | **52,1 ±2,48** | **56,7 ±2,56** | **61 ±2,89** |
| 2 mL *Cladonia arbuscula* | 5 ±0 | 5 ±0 | 5[a] ±0 | 5[a, b, c] ±0 | 5,5[a, b, c] ±0,5 | 6,5[a, b, c] ±0,35 | 7,8[a, b, c] ±0,27 | 10,3[a, b, c] ±0,45 | 11,4[a, b, c] ±0,74 | 13,9[a, c] ±0,89 | 15,4[a, c] ±1,85 | 17,1[a, b, c] ±2,3 | 19,1[a, c] ±3,17 | 20,9[a, c] ±3,97 | 22,9[a, c] ±4,75 | 24,5[a, c] ±5,62 | 26,3[a] ±6,38 | 28,3 ±7,22 | 30,3 ±8,3 |
| 2 mL *C. rangiferina* | 5 ±0 | 5,3 ±0,27 | 8,1[a, d, e, f, g] ±0,74 | 10,4[a, e, f] ±0,42 | 13,5[a, e, f] ±0,5 | 16,4[a, e] ±0,42 | 19,2[a, e] ±0,91 | 22,7[a, e] ±0,97 | 25,8[a, e] ±0,76 | 29,4[a, e] ±0,82 | 33,2[a, e] ±1,2 | 36[a, e] ±1 | 40,3[a, e] ±1,04 | 43,2[a, e] ±1,04 | 47,1[a, e] ±0,96 | 50,5[a, e] ±0,94 | 53,8[a, e] ±0,97 | 57,5[e, f] ±1,7 | 61,1[e] ±0,82 |
| 2 mL *C. digitata* | 5 ±0 | 5 ±0 | 7,1 ±0,22 | 9,2[b] ±0,45 | 12,4[b, h] ±0,89 | 15,4[b, h] ±0,82 | 18,2[b, h] ±1,44 | 21,7[b, h] ±1,25 | 25,2[b, h] ±1,92 | 28,5[h] ±1,62 | 32,1[h] ±1,82 | 35,7[b, h] ±1,82 | 38,4[h] ±2,16 | 42,7[h] ±,02 | 45,8[h] ±2,33 | 49,5[h] ±1,46 | 53,1[h] ±1,24 | 56,2[h] ±1,04 | 61[h] ±1,46 |
| 2 mL *C. uncialis* | 5 ±0 | 5 ±0 | 5[d] ±0 | 5,9 ±0,22 | 7,5 ±0,5 | 9,5 ±0,5 | 10,9 ±0,65 | 12,5 ±0,94 | 14,7 ±0,91 | 16,7 ±0,84 | 19,4 ±0,65 | 21 ±0,35 | 23,6 ±1,14 | 25,1 ±0,96 | 27,8 ±1,2 | 29,1 ±0,96 | 31,3 ±1,25 | 33,6 ±1,39 | 36,2 ±1,25 |
| 2 mL *Cetraria islandica* | 5 ±0 | 5,1 ±0,22 | 6,9 ±0,55 | 9,7[c, i] ±1,25 | 12,2[c, i] ±1,44 | 15,8[c, i] ±2,31 | 19,1[c, i] ±2,7 | 22,3[c, i] ±3,19 | 25,8[c, i] ±3,33 | 29[c, i] ±3,48 | 33,2[c, i] ±3,63 | 35,9[c, i] ±3,68 | 39,9[c, i] ±3,13 | 43,1[c, i, j] ±3,76 | 46,8[c, i, j] ±3,29 | 49,7[c, i] ±3,27 | 53,1[c, i] ±2,9 | 56,3[i] ±3,05 | 60,2[i] ±2,39 |
| 2 mL *Pseudevernia furfuracea* | 5 ±0 | 5 ±0 | 5[e] ±0 | 5,2[e, i] ±0,45 | 5,4[e, h, i] | 5,5[e, h, i] | 5,6[e, h, i] | 6[e, h, i] ±0,71 | 6,7[e, h, i] | 7,5[e, h, i] | 8,2[e, h, i] | 9,3[e, h, i] | 10[e, h, i] ±1,22 | 10,9[e, h, i] | 12[e, h, i] ±1,54 | 12,6[e, h, i] | 13,1[e, h, i] | 13,9[e, h, i] | 14,4[e, h, i] ±1,82 |

| | | | | | | | | | | | | | | | | | | | |
|---|---|---|---|---|---|---|---|---|---|---|---|---|---|---|---|---|---|---|---|
| | | | | | ±0,55 | ±0,5 | ±0,55 | | ±0,84 | ±1 | ±1,15 | ±1,15 | | ±1,34 | | ±1,39 | ±1,39 | ±1,56 | |
| 2 mL *C. arbuscula* + *C. rangiferina* + *C. digitata* | 5<br>±0 | 5<br>±0 | 5$^{f}$<br>±0 | 5,5$^{f}$<br>±0,5 | 7$^{f}$<br>±0,94 | 8,6<br>±0,42 | 9,9<br>±0,65 | 11,6<br>±0,74 | 13,1<br>±0,96 | 14,6<br>±0,65 | 17,2<br>±0,76 | 19,1<br>±1,34 | 21<br>±1,12 | 22,8$^{j}$<br>±1,44 | 24,8$^{j}$<br>±1,04 | 27,1<br>±1,95 | 28,9<br>±1,82 | 30,5$^{f}$<br>±1,66 | 33<br>±1,58 |
| 2 mL *C. arbuscula* + *C. rangiferina* + *C. digitata* + *C. uncialis* + *C. islandica* | 5<br>±0 | 5<br>±0 | 5$^{g}$<br>±0 | 5,9<br>±0,55 | 7,9<br>±0,55 | 9,3<br>±0,97 | 11<br>±1,41 | 12,8<br>±1,75 | 15,1<br>±1,75 | 16,8<br>±1,99 | 18,7<br>±1,72 | 21,5<br>±2,74 | 24,1<br>±2,48 | 26,3<br>±2,71 | 29,6<br>±2,95 | 31,3<br>±3,01 | 34,5<br>±3,52 | 36,4<br>±3,7 | 38,5<br>±3,1 |
| **3 mL acetone** | **5<br>±0** | **5<br>±0** | **7,2<br>±0,27** | **10<br>±0** | **12,2<br>±0,45** | **15,8<br>±0,27** | **19<br>±0,94** | **21,7<br>±0,91** | **25,1<br>±0,74** | **28,9<br>±1,14** | **32,5<br>±1,06** | **36,4<br>±1,24** | **40,3<br>±1,1** | **43,6<br>±1,56** | **48,5<br>±1,27** | **52,2<br>±1,99** | **55,5<br>±2,03** | **59,7<br>±1,48** | **63,8<br>±1,64** |
| 3 mL *Cladonia arbuscula* | 5<br>±0 | 5<br>±0 | 5<br>±0 | 5$^{b}$<br>±0 | 5$^{a, b}$<br>±0 | 5,6$^{b}$<br>±0,65 | 6,4$^{b}$<br>±0,65 | 8,2$^{b}$<br>±1,15 | 10$^{b}$<br>±0,5 | 11,7$^{b}$<br>±0,57 | 14,3$^{b}$<br>±1,2 | 15,7$^{b}$<br>±0,84 | 17,7$^{b}$<br>±0,91 | 19,6$^{b}$<br>±0,82 | 21,1$^{b}$<br>±1,14 | 23,3$^{b}$<br>±1,6 | 25,1$^{b}$<br>±1,78 | 27<br>±2,03 | 29,6<br>±2,33 |
| 3 mL *C. rangiferina* | 5<br>±0 | 5<br>±0 | 6,7<br>±0,76 | 9,7<br>±0,45 | 12$^{a, c}$<br>±0,94 | 14,6$^{c}$<br>±0,74 | 17,3$^{c}$<br>±1,15 | 20,5$^{c}$<br>±1 | 23,2$^{c}$<br>±0,91 | 26,1$^{c}$<br>±1,02 | 29,7$^{c}$<br>±1,1 | 32,9$^{c}$<br>±1,29 | 36,9$^{c}$<br>±1,08 | 39,6$^{c}$<br>±1,08 | 42,5$^{c}$<br>±0,79 | 45,9$^{c}$<br>±,64 | 49,4$^{c}$<br>±1,71 | 52,9$^{c}$<br>±0,74 | 57$^{c}$<br>±1,66 |
| 3 mL *C. digitata* | 5<br>±0 | 5,2<br>±0,45 | 6,5<br>±0,5 | 8,2<br>±0,27 | 9,9<br>±0,55 | 12,7<br>±1,04 | 14,5<br>±1,06 | 17,7<br>±1,35 | 19,8<br>±1,35 | 23,1<br>±1,52 | 26,3<br>±2,66 | 30,2<br>±3,09 | 33,9<br>±3,27 | 37,2$^{d}$<br>±3,68 | 40,3$^{d}$<br>±2,86 | 44,7$^{d}$<br>±2,97 | 48,8$^{d}$<br>±2,66 | 52,5$^{d}$<br>±2,55 | 56,8$^{d}$<br>±2,97 |
| 3 mL *C. uncialis* | 5<br>±0 | 5<br>±0 | 5<br>±0 | 5,4<br>±0,55 | 6,6<br>±0,65 | 7,6<br>±0,96 | 8,9<br>±1,52 | 10,4<br>±1,24 | 12,1<br>±1,14 | 14,5<br>±1,17 | 16,8<br>±0,91 | 18,5<br>±1,77 | 19,9<br>±1,39 | 21,8<br>±1,1 | 24,1<br>±1,08 | 26<br>±1,84 | 27,6<br>±0,89 | 29,9<br>±1,29 | 32,1<br>±1,56 |
| 3 mL *Cetraria islandica* | 5<br>±0 | 5,2<br>±0,45 | 7,2<br>±0,27 | 10,2$^{b, e, f}$<br>±0,45 | 12,6$^{b, e}$<br>±0,42 | 16,5$^{b, e}$<br>±0,94 | 20,1$^{b, e}$<br>±1,08 | 23,3$^{b, e}$<br>±1,15 | 26,7$^{b, e}$<br>±1,15 | 29,7$^{b, e}$<br>±1,2 | 33,6$^{b, e}$<br>±1,39 | 36$^{b, e}$<br>±1,41 | 39,6$^{b, e}$<br>±1,64 | 42,6$^{b, e}$<br>±1,64 | 46$^{b, e}$<br>±1,84 | 49,2$^{b, e}$<br>±2,02 | 51,7$^{b, e}$<br>±1,6 | 55,1$^{e}$<br>±1,98 | 58,4$^{e}$<br>±2,43 |
| 3 mL *Pseudevernia furfuracea* | 5<br>±0 | 5<br>±0 | 5<br>±0 | 5$^{e}$<br>±0 | 5$^{c, e}$<br>±0 | 5$^{c, e}$<br>±0 | 5,1$^{c, e}$<br>±0,22 | 5,5$^{c, e}$<br>±0,71 | 5,6$^{c, e}$<br>±0,89 | 5,8$^{c, e}$<br>±1,15 | 6$^{c, e}$<br>±1,54 | 6,6$^{c, e}$<br>±1,47 | 7,4$^{c, e}$<br>±1,82 | 8,1$^{c, d, e}$<br>±1,75 | 8,7$^{c, d, e}$<br>±1,82 | 9,3$^{c, d, e}$<br>±1,6 | 9,9$^{c, d, e}$<br>±1,64 | 10,2$^{c, d, e}$<br>±1,4 | 10,6$^{c, d, e}$<br>±1,39 |
| 3 mL *C. arbuscula* + *C. rangiferina* + *C. digitata* | 5<br>±0 | 5<br>±0 | 5<br>±0 | 5,2<br>±0,45 | 6,6<br>±0,82 | 8,4<br>±0,82 | 10<br>±0,94 | 11,5<br>±0,71 | 12,9<br>±0,96 | 14,9<br>±0,74 | 16,7<br>±1,15 | 19<br>±1,62 | 20<br>±1,77 | 22,6<br>±2,51 | 24,6<br>±2,38 | 26,1<br>±2,1 | 28,4<br>±1,92 | 29,7<br>±2,22 | 31,5<br>±2,4 |
| 3 mL *C. arbuscula* + *C. rangiferina* + *C. digitata* + *C. uncialis* + *C. islandica* | 5<br>±0 | 5<br>±0 | 5<br>±0 | 5$^{f}$<br>±0 | 6,6<br>±0,55 | 8,4<br>±0,22 | 9,8<br>±0,27 | 11,3<br>±0,57 | 12,9<br>±0,74 | 14,9<br>±,65 | 16,3<br>±0,76 | 19,1<br>±0,89 | 20,7<br>±0,97 | 22,3<br>±1,44 | 24,5<br>±2,09 | 26,1<br>±1,71 | 27,9<br>±1,47 | 29,9<br>±1,88 | 31,9<br>±2,38 |
| **4 mL acetone** | **5<br>±0** | **5<br>±0** | **5,8<br>±0,84** | **7,3<br>±1,72** | **9,5<br>±1,62** | **11,8<br>±2,14** | **13,6<br>±2,68** | **15,9<br>±3,36** | **19<br>±4,23** | **21,5<br>±4,78** | **25,3<br>±5,82** | **28,2<br>±6,19** | **32,2<br>±6,87** | **35,7<br>±7,16** | **39,7<br>±7,06** | **43,6<br>±8,12** | **46,9<br>±7,82** | **51,1<br>±7,51** | **55,8<br>±8,08** |
| 4 mL *Cladonia arbuscula* | 5<br>±0 | 5<br>±0 | 5<br>±0 | 5<br>±0 | 5$^{a}$<br>±0 | 5,3$^{a}$<br>±0,45 | 6,4<br>±1,24 | 7,9<br>±1,29 | 9,3<br>±1,64 | 11<br>±1,41 | 13<br>±1,58 | 14,5<br>±1,87 | 16,4<br>±2,86 | 17,7<br>±2,97 | 19,4<br>±3,07 | 20,7<br>±3,77 | 22,6<br>±4,36 | 23,8<br>±4,31 | 26,5<br>±5,09 |
| 4 mL *C. rangiferina* | 5<br>±0 | 5<br>±0 | 6<br>±0,79 | 7,9<br>±0,74 | 9,7<br>±0,76 | 11,3<br>±0,97 | 13,6$^{b}$<br>±0,89 | 16$^{b}$<br>±0,79 | 18,4$^{b}$<br>±1,6 | 21,8$^{b}$<br>±1,35 | 24,5$^{b}$<br>±1,46 | 27,2$^{b}$<br>±1,64 | 31,1$^{b}$<br>±2,01 | 33,9$^{b}$<br>±1,82 | 37,4$^{b}$<br>±1,98 | 40,8$^{b}$<br>±2,17 | 45$^{b}$<br>±2,4 | 49,1$^{b}$<br>±2,68 | 52,4$^{b}$<br>±2,97 |
| 4 mL *C. digitata* | 5<br>±0 | 5<br>±0 | 6,4<br>±0,96 | 8,2<br>±1,64 | 10,7$^{a, c}$<br>±2,2 | 13,6$^{a, c}$<br>±2,68 | 15,5$^{c}$<br>±3,24 | 18$^{c}$<br>±3,55 | 21,1$^{c}$<br>±3,94 | 23,7$^{c}$<br>±4,74 | 26,5$^{c}$<br>±5,76 | 30,4$^{c}$<br>±6,38 | 32,5$^{c}$<br>±7,22 | 36,2$^{c}$<br>±8,42 | 38,2$^{c}$<br>±9,28 | 42,1$^{c}$<br>±10,84 | 45,6$^{c}$<br>±10,84 | 48$^{c}$<br>±12,17 | 52$^{c}$<br>±12,16 |
| 4 mL *C. uncialis* | 5<br>±0 | 5<br>±0 | 5<br>±0 | 5<br>±0 | 5,1<br>±0,22 | 5,6<br>±0,55 | 6,5<br>±1 | 7,3<br>±1,57 | 8,3<br>±1,89 | 9,6<br>±2,58 | 11,4<br>±3,66 | 12,6<br>±4,44 | 13,8<br>±5,08 | 14,9<br>±5,57 | 16<br>±6,17 | 17,9<br>±7,34 | 19,2<br>±8,04 | 20,6<br>±8,81 | 22,6<br>±9,96 |
| 4 mL *Cetraria islandica* | 5<br>±0 | 5,1<br>±0,22 | 6,8<br>±1,15 | 9,1<br>±2,36 | 11<br>±3,54 | 13,9$^{d}$<br>±5,14 | 16,2$^{d}$<br>±6,43 | 18,9$^{d}$<br>±7,86 | 21,4$^{d}$<br>±9,3 | 23,8$^{d}$<br>±10,62 | 26,7$^{d}$<br>±12,29 | 28,9$^{d}$<br>±13,44 | 31,7$^{d}$<br>±15,02 | 34,1$^{d}$<br>±16,35 | 37,1$^{d}$<br>±18 | 39,4$^{d}$<br>±19,29 | 41,8$^{d}$<br>±20,64 | 44,3$^{d}$<br>±22,02 | 46,6<br>±23,31 |
| 4 mL *Pseudevernia furfuracea* | 5<br>±0 | 5<br>±0 | 5<br>±0 | 5<br>±0 | 5$^{c}$<br>±0 | 5$^{c, d}$<br>±0 | 5$^{b, c, d}$<br>±0 | 5$^{b, c, d}$<br>±0 | 5$^{b, c, d}$<br>±0 | 5$^{b, c, d}$<br>±0 | 5$^{b, c, d}$<br>±0 | 5$^{b, c, d}$<br>±0 | 5$^{b, c, d}$<br>±0 | 5$^{b, c, d}$<br>±0 | 5$^{b, c, d}$<br>±0 | 5,3$^{b, c, d}$<br>±0,45 | 5,9$^{b, c, d}$<br>±0,22 | 6$^{b, c, d}$<br>±0 | 6,7$^{b, c, d}$<br>±0,45 |
| 4 mL *C. arbuscula* + *C. rangiferina* + *C. digitata* | 5<br>±0 | 5<br>±0 | 5<br>±0 | 5,1<br>±0,22 | 5,6<br>±0,65 | 7,4<br>±1,52 | 8,7<br>±2,31 | 10,6<br>±2,72 | 12,2<br>±2,91 | 14<br>±2,57 | 15,6<br>±2,88 | 17,6<br>±2,63 | 18,9<br>±2,33 | 21,5<br>±2,67 | 22,6<br>±2,95 | 25,2<br>±2,56 | 27,2<br>±2,08 | 28,5<br>±2,76 | 30,8<br>±2,68 |
| 4 mL *C. arbuscula* + *C. rangiferina* + *C. digitata* + *C. uncialis* + *C. islandica* | 5<br>±0 | 5<br>±0 | 5<br>±0 | 5<br>±0 | 5,9<br>±0,55 | 6,9<br>±1,14 | 7,9<br>±1,14 | 9,2<br>±1,25 | 11,3<br>±1,52 | 12,5<br>±1,9 | 14,1<br>±2,01 | 15,5<br>±2,47 | 17,5<br>±2,47 | 18,9<br>±3,25 | 21,1<br>±4,16 | 22,5<br>±3,98 | 24,3<br>±4,58 | 25,7<br>±5,71 | 27,5<br>±6,14 |
