# Supplementary material for: Antifungal and Profungal Potentials of Acetone-Extracted Complexes of Lichen Substances on Macromycete Heterobasidion parviporum Mycelium Growth Dynamics—The First In Vitro Approach
Source: Molecules. 2026 Aug 18;31(16):2882. doi: 10.3390/molecules31162882 (PMC13515570; doi:10.3390/molecules31162882)
Supplement: Supplementary file 1 [file molecules-31-02882-s001.zip › Table_S22_Supplementation_methods.pdf]

**Table S22:** Comparison of *Heterobasidion parviporum* mycelial growth dynamics between application onto the surface of solidified Hansen's medium and incorporation into the liquid medium before solidification: (A) solvent-free controls and (B) cultures receiving equivalent volumes (1–3 mL) of acetone or eight lichen acetone-extract variants. Cultures were incubated at 28 °C and measured at 12 h intervals.

Statistically significant differences in lichen extracts between the different supplementation methods at a given measurement time are shown in red.
ONTO – dose of extract supplemented onto the solidified surface of the medium;
INTO – dose of extract mixed with the medium before solidification;
mixed 3 lichen species – extract obtained from mixed thalli of *Cladonia arbuscula* + *Cladonia rangiferina* + *Cladonia digitata*;
mixed 5 lichen species – extract obtained from mixed thalli of *Cladonia arbuscula* + *Cladonia rangiferina* + *Cladonia digitata* + *Cladonia uncialis* + *Cetraria islandica*;
"-" – no measurement or inability to compare the diameter of the mycelium in samples with extract to the diameter of the mycelium in samples with an equivalent dose of the acetone at a given measurement time.

**A *Heterobasidion parviporum***
Average mycelium diameter [mm] at a given measurement time [h]
± standard deviation in sample [mm]

**Control sample without acetone (0 mL acetone)**

| Measurements time | 12 | 24 | 36 | 48 | 60 | 72 | 84 | 96 | 108 | 120 | 132 | 144 | 156 | 168 | 180 | 192 | 204 | 216 | 228 |
|---|---|---|---|---|---|---|---|---|---|---|---|---|---|---|---|---|---|---|---|
| control sample in supplementation method ONTO the medium | 5 ±0 | **5,8 ±0,27** | **7,6 ±0,42** | **10,7 ±0,57** | **12,7 ±0,45** | **15,9 ±0,89** | 18,3 ±0,84 | 21,8 ±0,76 | 24,2 ±1,25 | 26,4 ±1,14 | 29,1 ±0,82 | 32,3 ±1,44 | 34 ±1 | 36,4 ±1,56 | 39 ±1,54 | 41,3 ±1,35 | 43,5 ±1,37 | 46,1 ±1,71 | 48,7 ±1,3 |
| control sample in supplementation method INTO the medium | 5 ±0 | 5 ±0 | 5,5 ±0,87 | 7,8 ±1,35 | 10,5 ±1,12 | 12,9 ±1,67 | 15,8 ±2,02 | 19 ±2,26 | 22,5 ±2,69 | 26,3 ±2,89 | 29,5 ±3,22 | 33,3 ±3,65 | 37,5 ±4,09 | **40,9 ±4,26** | **45,5 ±4,97** | **49,9 ±5,07** | **53,6 ±5,96** | **58,4 ±5,91** | **62,4 ±6,1** |

**B *Heterobasidion parviporum***
Average mycelium diameter [mm] at a given measurement time [h]
± standard deviation in sample [mm]

| Measurement times | solvent | | *C. arbuscula* | | *C. rangiferina* | | *C. digitata* | | *C. uncialis* | | *C. islandica* | | *P. furfuracea* | | mixed 3 lichen species | | mixed 5 lichen species | |
|---|---|---|---|---|---|---|---|---|---|---|---|---|---|---|---|---|---|---|
| | **1 mL acetone** | | **1 mL lichen extract** | | | | | | | | | | | | | | | |
| | **ONTO** | **INTO** | **ONTO** | **INTO** | **ONTO** | **INTO** | **ONTO** | **INTO** | **ONTO** | **INTO** | **ONTO** | **INTO** | **ONTO** | **INTO** | **ONTO** | **INTO** | **ONTO** | **INTO** |
| 12 | 5 ±0 | 5 ±0 | 5 ±0 | 5 ±0 | 5 ±0 | 5 ±0 | 5 ±0 | 5 ±0 | 5 ±0 | 5 ±0 | 5 ±0 | 5 ±0 | 5 ±0 | 5 ±0 | 5 ±0 | 5 ±0 | 5 ±0 | 5 ±0 |
| 24 | 5 ±0 | 5 ±0 | 5 ±0 | 5 ±0 | 5 ±0 | 5 ±0 | 5 ±0 | 5 ±0 | 5 ±0 | 5 ±0 | 5 ±0 | 5 ±0 | 5 ±0 | 5 ±0 | 5 ±0 | 5 ±0 | 5 ±0 | 5 ±0 |
| 36 | 5 ±0 | 5 ±0 | 5 ±0 | 5 ±0 | 5 ±0 | **6,4 ±0,55** | 5,9 ±0,22 | 6,7 ±0,67 | 5 ±0 | 5,6 ±0,55 | 5 ±0 | **6,3 ±0,67** | 5 ±0 | 5 ±0 | 5,4 ±0,55 | 5,9 ±0,22 | 5 ±0 | 5 ±0 |
| 48 | 5,8 ±0,27 | 7 ±0,94 | 5 ±0 | 5,7 ±0,67 | 5 ±0 | **8,5 ±0,61** | 6,8 ±0,84 | 8,8 ±1,44 | 5 ±0 | **7,3 ±1,68** | 5 ±0 | **9,3 ±1,4** | 5 ±0 | **5,9 ±0,74** | 6,2 ±1,44 | 7,1 ±0,74 | 5 ±0 | **6,7 ±0,45** |

| | | | | | | | | | | | | | | | | | | |
|---|---|---|---|---|---|---|---|---|---|---|---|---|---|---|---|---|---|---|
| 60 | 6,8 ±0,45 | 8,9 ±1,02 | 5 ±0 | 6,8 ±1,04 | 5,3 ±0,45 | 10,8 ±0,45 | 8,4 ±1,08 | 11,1 ±1,56 | 5 ±0 | 8,6 ±2,33 | 5 ±0 | 11,4 ±2,04 | 5 ±0 | 7,2 ±0,57 | 7,4 ±1,98 | 9 ±0,35 | 5 ±0 | 8,7 ±0,45 |
| 72 | 7,7 ±0,67 | 11,5 ±0,87 | 5 ±0 | 8,6 ±1,19 | 5,9 ±0,89 | 13,4 ±0,82 | 10,2 ±1,2 | 13,7 ±1,64 | 5 ±0 | 10,5 ±3,32 | 5 ±0 | 15,3 ±2,97 | 5 ±0 | 8,2 ±1,3 | 8,4 ±2,41 | 11 ±0,71 | 5 ±0 | 10,5 ±0,5 |
| 84 | 9,1 ±0,82 | 13,3 ±1,6 | 5,3 ±0,45 | 11,1 ±1,43 | 6,7 ±1,35 | 16,6 ±0,82 | 12,6 ±1,14 | 16,3 ±2,08 | 5 ±0 | 12,1 ±4,16 | 5 ±0 | 18,7 ±3,68 | 5 ±0 | 9,5 ±1,5 | 9,7 ±2,59 | 12,6 ±1,24 | 5 ±0 | 12,7 ±0,45 |
| 96 | 10,4 ±0,65 | 15,7 ±2,2 | 5,4 ±0,55 | 12,9 ±1,34 | 7,8 ±1,04 | 19,7 ±1,35 | 14,5 ±1,11 | 19,4 ±2,95 | 5 ±0 | 14,2 ±5,39 | 5 ±0 | 22,4 ±4,11 | 5 ±0 | 10,9 ±2,04 | 10,5 ±3,67 | 14,9 ±1,67 | 5 ±0 | 14,9 ±0,42 |
| 108 | 11,8 ±1,04 | 18,7 ±2,59 | 5,7 ±0,97 | 15,8 ±2,33 | 9,1 ±1,29 | 23,5 ±1,12 | 16,5 ±1,32 | 22,3 ±3,38 | 5 ±0 | 16 ±6,32 | 5 ±0 | 26,4 ±4,35 | 5 ±0 | 12,3 ±2,39 | 11,4 ±4,29 | 16,9 ±1,24 | 5 ±0 | 17,3 ±0,57 |
| 120 | 12,8 ±1,48 | 21,6 ±3,09 | 6,2 ±1,64 | 17,5 ±2,81 | 10,6 ±1,29 | 26,4 ±1,29 | 18,6 ±1,47 | 25,6 ±4,17 | 5,2 ±0,45 | 17,6 ±7,22 | 5 ±0 | 30 ±4,81 | 5 ±0 | 13,6 ±2,9 | 12,4 ±4,89 | 19,5 ±1,58 | 5 ±0 | 20,1 ±0,42 |
| 132 | 13,8 ±1,64 | 24,7 ±3,78 | 6,4 ±1,95 | 20,5 ±2,96 | 11,7 ±1,4 | 30,2 ±1,68 | 21,1 ±1,29 | 28,7 ±4,55 | 5,2 ±0,45 | 20,1 ±8,73 | 5 ±0 | 34,7 ±5,31 | 5 ±0 | 14,8 ±3,13 | 13,3 ±5,69 | 22 ±1,22 | 5 ±0 | 22,3 ±1,2 |
| 144 | 15,5 ±1,87 | 27,8 ±4,09 | 6,8 ±2,25 | 22,3 ±3,91 | 13,4 ±1,71 | 34,8 ±1,3 | 22,6 ±1,64 | 32,4 ±5,28 | 5,4 ±0,89 | 22,5 ±9,94 | 5 ±0 | 37,5 ±4,96 | 5 ±0 | 16,5 ±3,16 | 13,9 ±6,07 | 24,6 ±1,08 | 5 ±0 | 25 ±0,79 |
| 156 | 17,1 ±1,75 | 31,5 ±4,68 | 7,3 ±2,73 | 24,8 ±4,86 | 14,9 ±1,98 | 38,5 ±2,03 | 25,3 ±1,86 | 35,8 ±5,84 | 5,6 ±1,34 | 24,6 ±11,22 | 5 ±0 | 41,8 ±5,03 | 5 ±0 | 18,2 ±3,38 | 14,9 ±7,22 | 27,3 ±0,91 | 5 ±0 | 27,9 ±0,96 |
| 168 | 17,8 ±1,89 | 35,2 ±4,83 | 8,1 ±3,29 | 26,9 ±6,07 | 17,3 ±2,2 | 41,5 ±1,66 | 27,1 ±1,64 | 40,3 ±6,05 | 5,6 ±1,34 | 26,8 ±12,34 | 5 ±0 | 45,8 ±5,19 | 5 ±0 | 19,5 ±3,61 | 15,5 ±8,07 | 30 ±1,77 | 5 ±0 | 30,1 ±0,74 |
| 180 | 19,9 ±1,98 | 39,3 ±5,51 | 8,8 ±3,77 | 28,4 ±7 | 19 ±2,47 | 45,4 ±1,64 | 30,1 ±2,07 | 42,3 ±6,26 | 5,8 ±1,79 | 28,5 ±13,22 | 5 ±0 | 49,6 ±4,79 | 5 ±0 | 21,4 ±3,75 | 16,5 ±8,76 | 31,5 ±0,79 | 5 ±0 | 32,9 ±1,56 |
| 192 | 20,7 ±2,11 | 43,4 ±6,46 | 9,6 ±4,51 | 31,2 ±7,46 | 21,3 ±2,61 | 49,4 ±1,64 | 32,1 ±2,07 | 46,9 ±7,12 | 5,8 ±1,79 | 30,8 ±14,71 | 5 ±0 | 53,4 ±5,76 | 5 ±0 | 22,4 ±3,75 | 17,4 ±9,48 | 34,8 ±1,04 | 5 ±0 | 35,8 ±1,75 |
| 204 | 22,9 ±2,01 | 46,6 ±6,09 | 10,5 ±5,15 | 33,4 ±7,85 | 23,5 ±2,78 | 53,3 ±1,6 | 34,4 ±2,16 | 51,1 ±7,44 | 6,3 ±2,64 | 33 ±15,95 | 5 ±0 | 58,1 ±5,27 | 5 ±0 | 24,4 ±3,9 | 18,2 ±10,37 | 36,7 ±0,76 | 5 ±0 | 38,5 ±2,06 |
| 216 | 24,1 ±2,38 | 50,5 ±6,05 | 11,3 ±5,43 | 35,2 ±7,83 | 26,4 ±3,03 | 57,7 ±1,6 | 36,9 ±2,3 | 54,5 ±7,88 | 6,6 ±3,05 | 35,9 ±17,71 | 5 ±0 | 62 ±5,44 | 5 ±0 | 25,9 ±4,02 | 19 ±11,25 | 38,7 ±0,97 | 5 ±0 | 42,2 ±2,59 |
| 228 | 26,2 ±1,64 | 54,7 ±5,85 | 12,3 ±5,74 | 38,1 ±8,88 | 28,7 ±3,51 | 61,7 ±1,15 | 39,7 ±2,64 | 58,5 ±8,76 | 7,2 ±4,13 | 38,4 ±19,11 | 5 ±0 | 66,4 ±4,84 | 5 ±0 | 27,1 ±4,32 | 20,3 ±12,3 | 41 ±0,61 | 5 ±0 | 43,4 ±2,16 |
| | 2 mL acetone | | 2 mL lichen extract | | | | | | | | | | | | | | | |
| | ONTO | INTO | ONTO | INTO | ONTO | INTO | ONTO | INTO | ONTO | INTO | ONTO | INTO | ONTO | INTO | ONTO | INTO | ONTO | INTO |
| 12 | 5 ±0 | 5 ±0 | 5 ±0 | 5 ±0 | 5 ±0 | 5 ±0 | 5 ±0 | 5 ±0 | 5 ±0 | 5 ±0 | 5 ±0 | 5 ±0 | 5 ±0 | 5 ±0 | 5 ±0 | 5 ±0 | 5 ±0 | 5 ±0 |
| 24 | 5 ±0 | 5 ±0 | 5 ±0 | 5 ±0 | 5 ±0 | 5,3 ±0,27 | 5 ±0 | 5 ±0 | 5 ±0 | 5 ±0 | 5 ±0 | 5,1 ±0,22 | 5 ±0 | 5 ±0 | 5 ±0 | 5 ±0 | 5 ±0 | 5 ±0 |
| 36 | 5 ±0 | 6 ±0,35 | 5 ±0 | ±5 0 | 5 ±0 | 8,1 ±0,74 | 5 ±0 | 7,1 ±0,22 | 5 ±0 | 5 ±0 | 5 ±0 | 6,9 ±0,55 | 5 ±0 | 5 ±0 | 5 ±0 | 5 ±0 | 5 ±0 | 5 ±0 |
| 48 | 5 ±0 | 8,4 ±0,65 | 5 ±0 | 5 ±0 | 5 ±0 | 10,4 ±0,42 | 5 ±0 | 9,2 ±0,45 | 5 ±0 | 5,9 ±0,22 | 5 ±0 | 9,7 ±1,25 | 5 ±0 | 5,2 ±0,45 | 5 ±0 | 5,5 ±0,5 | 5 ±0 | 5,9 ±0,55 |
| 60 | 5 ±0 | 10,8 ±0,57 | 5 ±0 | 5,5 ±0,5 | 5 ±0 | 13,5 ±0,5 | 5 ±0 | 12,4 ±0,89 | 5 ±0 | 7,5 ±0,5 | 5 ±0 | 12,2 ±1,44 | 5 ±0 | 5,4 ±0,55 | 5 ±0 | 7 ±0,94 | 5 ±0 | 7,9 ±0,55 |
| 72 | 5 ±0 | 14,1 ±0,55 | 5 ±0 | 6,5 ±0,35 | 5 ±0 | 16,4 ±0,42 | 5 ±0 | 15,4 ±0,82 | 5 ±0 | 9,5 ±0,5 | 5 ±0 | 15,8 ±2,31 | 5 ±0 | 5,5 ±0,5 | 5 ±0 | 8,6 ±0,42 | 5 ±0 | 9,3 ±0,97 |

| | | | | | | | | | | | | | | | | | | |
|---|---|---|---|---|---|---|---|---|---|---|---|---|---|---|---|---|---|---|
| 84 | 5,2 ±0,45 | **16,3 ±0,45** | 5 ±0 | **7,8 ±0,27** | 5 ±0 | **19,2 ±0,91** | 5 ±0 | **18,2 ±1,44** | 5 ±0 | **10,9 ±0,65** | 5 ±0 | **19,1 ±2,7** | 5 ±0 | 5,6 ±0,55 | 5 ±0 | **9,9 ±0,65** | 5 ±0 | **11 ±1,41** |
| 96 | 5,7 ±0,84 | **19,8 ±1,25** | 5 ±0 | **10,3 ±0,45** | 5 ±0 | **22,7 ±0,97** | 5 ±0 | **21,7 ±1,25** | 5 ±0 | **12,5 ±0,94** | 5 ±0 | **22,3 ±3,19** | 5 ±0 | **6 ±0,71** | 5 ±0 | **11,6 ±0,74** | 5 ±0 | **12,8 ±1,75** |
| 108 | 6,4 ±1,39 | **23 ±1,66** | 5,1 ±0,22 | **11,4 ±0,74** | 5,1 ±0,22 | **25,8 ±0,76** | 5 ±0 | **25,2 ±1,92** | 5 ±0 | **14,7 ±0,91** | 5 ±0 | **25,8 ±3,33** | 5 ±0 | **6,7 ±0,84** | 5 ±0 | **13,1 ±0,96** | 5 ±0 | **15,1 ±1,75** |
| 120 | 6,9 ±1,98 | **26,1 ±1,14** | 5,2 ±0,45 | **13,9 ±0,89** | 5,3 ±0,67 | **29,4 ±0,82** | 5 ±0 | **28,5 ±1,62** | 5 ±0 | **16,7 ±0,84** | 5 ±0 | **29 ±3,48** | 5 ±0 | **7,5 ±1** | 5 ±0 | **14,6 ±0,65** | 5 ±0 | **16,8 ±1,99** |
| 132 | 8 ±2,37 | **29,7 ±1,35** | 5,4 ±0,65 | **15,4 ±1,85** | 5,6 ±1,34 | **33,2 ±1,2** | 5,2 ±0,45 | **32,1 ±1,82** | 5 ±0 | **19,4 ±0,65** | 5 ±0 | **33,2 ±3,63** | 5 ±0 | **8,2 ±1,15** | 5,1 ±0,22 | **17,2 ±0,76** | 5 ±0 | **18,7 ±1,72** |
| 144 | 9,3 ±2,59 | **33 ±1,58** | 6,1 ±0,74 | **17,1 ±2,3** | 5,8 ±1,79 | **36 ±1** | 5,4 ±0,89 | **35,7 ±1,82** | 5 ±0 | **21 ±0,35** | 5 ±0 | **35,9 ±3,68** | 5 ±0 | **9,3 ±1,15** | 5,3 ±0,67 | **19,1 ±1,34** | 5 ±0 | **21,5 ±2,74** |
| 156 | 10,8 ±3,37 | **37,4 ±2,04** | 6,5 ±1,12 | **19,1 ±3,17** | 6 ±2,24 | **40,3 ±1,04** | 5,6 ±0,89 | **38,4 ±2,16** | 5 ±0 | **23,6 ±1,14** | 5 ±0 | **39,9 ±3,13** | 5 ±0 | **10 ±1,22** | 5,4 ±0,89 | **21 ±1,12** | 5 ±0 | **24,1 ±2,48** |
| 168 | 12 ±3,48 | **40,4 ±1,78** | 6,9 ±1,29 | **20,9 ±3,97** | 6,4 ±3,13 | **43,2 ±1,04** | 6,3 ±1,89 | **42,7 ±2,02** | 5 ±0 | **25,1 ±0,96** | 5 ±0 | **43,1 ±3,76** | 5 ±0 | **10,9 ±1,34** | 5,4 ±0,89 | **22,8 ±1,44** | 5 ±0 | **26,3 ±2,71** |
| 180 | 13,5 ±4,23 | **44,9 ±2,51** | 7,5 ±1,58 | **22,9 ±4,75** | 6,8 ±4,02 | **47,1 ±0,96** | 7 ±2,42 | **45,8 ±2,33** | 5 ±0 | **27,8 ±1,2** | 5 ±0 | **46,8 ±3,29** | 5 ±0 | **12 ±1,54** | 5,6 ±1,34 | **24,8 ±1,04** | 5 ±0 | **29,6 ±2,95** |
| 192 | 15 ±4,3 | **48,7 ±2,36** | 8,8 ±1,35 | **24,5 ±5,62** | 7,2 ±4,92 | **50,5 ±0,94** | 7,7 ±2,89 | **49,5 ±1,46** | 5 ±0 | **29,1 ±0,96** | 5 ±0 | **49,7 ±3,27** | 5 ±0 | **12,6 ±1,39** | 5,8 ±1,3 | **27,1 ±1,95** | 5 ±0 | **31,3 ±3,01** |
| 204 | 16 ±4,83 | **52,1 ±2,48** | 9,7 ±1,2 | **26,3 ±6,38** | 7,6 ±4,92 | **53,8 ±0,97** | 9 ±3,59 | **53,1 ±1,24** | 5 ±0 | **31,3 ±1,25** | 5 ±0 | **53,1 ±2,9** | 5 ±0 | **13,1 ±1,39** | 6 ±1,54 | **28,9 ±1,82** | 5 ±0 | **34,5 ±3,52** |
| 216 | 18,2 ±5,39 | **56,7 ±2,56** | 10,7 ±1,35 | **28,3 ±7,22** | 8 ±6,71 | **57,5 ±1,7** | 10,3 ±4,22 | **56,2 ±1,04** | 5 ±0 | **33,6 ±1,39** | 5 ±0 | **56,3 ±3,05** | 5 ±0 | **13,9 ±1,56** | 6 ±1,54 | **30,5 ±1,66** | 5 ±0 | **36,4 ±3,7** |
| 228 | 20,7 ±5,43 | **61 ±2,89** | 11,8 ±1,57 | **30,3 ±8,3** | 8,3 ±7,38 | **61,1 ±0,82** | 11,4 ±4,55 | **61 ±1,46** | 5 ±0 | **36,2 ±1,25** | 5 ±0 | **60,2 ±2,39** | 5 ±0 | **14,4 ±1,82** | 6,2 ±1,68 | **33 ±1,58** | 5 ±0 | **38,5 ±3,1** |
| | **3 mL acetone** | | **3 mL lichen extract** | | | | | | | | | | | | | | | |
| | **ONTO** | **INTO** | - | - | - | - | **ONTO** | **INTO** | **ONTO** | **INTO** | **ONTO** | **INTO** | **ONTO** | **INTO** | **ONTO** | **INTO** | **ONTO** | **INTO** |
| 12 | 5 ±0 | 5 ±0 | - | - | - | - | 5 ±0 | 5 ±0 | 5 ±0 | 5 ±0 | 5 ±0 | 5 ±0 | 5 ±0 | 5 ±0 | 5 ±0 | 5 ±0 | 5 ±0 | 5 ±0 |
| 24 | 5 ±0 | 5 ±0 | - | - | - | - | 5 ±0 | 5,2 ±0,45 | 5 ±0 | 5 ±0 | 5 ±0 | 5,2 ±0,45 | 5 ±0 | 5 ±0 | 5 ±0 | 5 ±0 | 5 ±0 | 5 ±0 |
| 36 | 5 ±0 | **7,2 ±0,27** | - | - | - | - | 5 ±0 | **6,5 ±0,5** | 5 ±0 | 5 ±0 | 5 ±0 | **7,2 ±0,27** | 5 ±0 | 5 ±0 | 5 ±0 | 5 ±0 | 5 ±0 | 5 ±0 |
| 48 | 5 ±0 | **10 ±0** | - | - | - | - | 5 ±0 | **8,2 ±0,27** | 5 ±0 | 5,4 ±0,55 | 5 ±0 | **10,2 ±0,45** | 5 ±0 | 5 ±0 | 5 ±0 | 5,2 ±0,45 | 5 ±0 | 5 ±0 |
| 60 | 5 ±0 | **12,2 ±0,45** | - | - | - | - | 5 ±0 | **9,9 ±0,55** | 5 ±0 | **6,6 ±0,65** | 5 ±0 | **12,6 ±0,42** | 5 ±0 | 5 ±0 | 5 ±0 | **6,6 ±0,82** | 5 ±0 | **6,6 ±0,55** |
| 72 | 5 ±0 | **15,8 ±0,27** | - | - | - | - | 5 ±0 | **12,7 ±1,04** | 5 ±0 | **7,6 ±0,96** | 5 ±0 | **16,5 ±0,94** | 5 ±0 | 5 ±0 | 5 ±0 | **8,4 ±0,82** | 5 ±0 | **8,4 ±0,22** |
| 84 | 5 ±0 | **19 ±0,94** | - | - | - | - | 5 ±0 | **14,5 ±1,06** | 5 ±0 | **8,9 ±1,52** | 5 ±0 | **20,1 ±1,08** | 5 ±0 | 5,1 ±0,22 | 5 ±0 | **10 ±0,94** | 5 ±0 | **9,8 ±0,27** |
| 96 | 5 ±0 | **21,7 ±0,91** | - | - | - | - | 5 ±0 | **17,7 ±1,35** | 5 ±0 | **10,4 ±1,24** | 5 ±0 | **23,3 ±1,15** | 5 ±0 | 5,5 ±0,71 | 5 ±0 | **11,5 ±0,71** | 5 ±0 | **11,3 ±0,57** |

| | | | | | | | | | | | | | | | | | | |
|---|---|---|---|---|---|---|---|---|---|---|---|---|---|---|---|---|---|---|
| 108 | 5<br>±0 | 25,1<br>±0,74 | - | - | - | - | 5<br>±0 | 19,8<br>±1,35 | 5<br>±0 | 12,1<br>±1,14 | 5<br>±0 | 26,7<br>±1,15 | 5<br>±0 | 5,6<br>±0,89 | 5<br>±0 | 12,9<br>±0,96 | 5<br>±0 | 12,9<br>±0,74 |
| 120 | 5<br>±0 | 28,9<br>±1,14 | - | - | - | - | 5<br>±0 | 23,1<br>±1,52 | 5<br>±0 | 14,5<br>±1,17 | 5<br>±0 | 29,7<br>±1,2 | 5<br>±0 | 5,8<br>±1,15 | 5<br>±0 | 14,9<br>±0,74 | 5<br>±0 | 14,9<br>±0,65 |
| 132 | 5<br>±0 | 32,5<br>±1,06 | - | - | - | - | 5<br>±0 | 26,3<br>±2,66 | 5<br>±0 | 16,8<br>±0,91 | 5<br>±0 | 33,6<br>±1,39 | 5<br>±0 | 6<br>±1,54 | 5<br>±0 | 16,7<br>±1,15 | 5<br>±0 | 16,3<br>±0,76 |
| 144 | 5<br>±0 | 36,4<br>±1,24 | - | - | - | - | 5<br>±0 | 30,2<br>±3,09 | 5<br>±0 | 18,5<br>±1,77 | 5<br>±0 | 36<br>±1,41 | 5<br>±0 | 6,6<br>±1,47 | 5<br>±0 | 19<br>±1,62 | 5<br>±0 | 19,1<br>±0,89 |
| 156 | 5<br>±0 | 40,3<br>±1,1 | - | - | - | - | 5<br>±0 | 33,9<br>±3,27 | 5<br>±0 | 19,9<br>±1,39 | 5<br>±0 | 39,6<br>±1,64 | 5<br>±0 | 7,4<br>±1,82 | 5<br>±0 | 20<br>±1,77 | 5<br>±0 | 20,7<br>±0,97 |
| 168 | 5<br>±0 | 43,6<br>±1,56 | - | - | - | - | 5<br>±0 | 37,2<br>±3,68 | 5<br>±0 | 21,8<br>±1,1 | 5<br>±0 | 42,6<br>±1,64 | 5<br>±0 | 8,1<br>±1,75 | 5<br>±0 | 22,6<br>±2,51 | 5<br>±0 | 22,3<br>±1,44 |
| 180 | 5<br>±0 | 48,5<br>±1,27 | - | - | - | - | 5<br>±0 | 40,3<br>±2,86 | 5<br>±0 | 24,1<br>±1,08 | 5<br>±0 | 46<br>±1,84 | 5<br>±0 | 8,7<br>±1,82 | 5<br>±0 | 24,6<br>±2,38 | 5<br>±0 | 24,5<br>±2,09 |
| 192 | 5<br>±0 | 52,2<br>±1,99 | - | - | - | - | 5<br>±0 | 44,7<br>±2,97 | 5<br>±0 | 26<br>±1,84 | 5<br>±0 | 49,2<br>±2,02 | 5<br>±0 | 9,3<br>±1,6 | 5<br>±0 | 26,1<br>±2,1 | 5<br>±0 | 26,1<br>±1,71 |
| 204 | 5<br>±0 | 55,5<br>±2,03 | - | - | - | - | 5<br>±0 | 48,8<br>±2,66 | 5<br>±0 | 27,6<br>±0,89 | 5<br>±0 | 51,7<br>±1,6 | 5<br>±0 | 9,9<br>±1,64 | 5<br>±0 | 28,4<br>±1,92 | 5<br>±0 | 27,9<br>±1,47 |
| 216 | 5<br>±0 | 59,7<br>±1,48 | - | - | - | - | 5<br>±0 | 52,5<br>±2,55 | 5<br>±0 | 29,9<br>±1,29 | 5<br>±0 | 55,1<br>±1,98 | 5<br>±0 | 10,2<br>±1,4 | 5<br>±0 | 29,7<br>±2,22 | 5<br>±0 | 29,9<br>±1,88 |
| 228 | 5,3<br>±0,45 | 63,8<br>±1,64 | - | - | - | - | 5<br>±0 | 56,8<br>±2,97 | 5<br>±0 | 32,1<br>±1,56 | 5<br>±0 | 58,4<br>±2,43 | 5<br>±0 | 10,6<br>±1,39 | 5<br>±0 | 31,5<br>±2,4 | 5<br>±0 | 31,9<br>±2,38 |
