# Supplementary material for: Antifungal and Profungal Potentials of Acetone-Extracted Complexes of Lichen Substances on Macromycete Heterobasidion parviporum Mycelium Growth Dynamics—The First In Vitro Approach
Source: Molecules. 2026 Aug 18;31(16):2882. doi: 10.3390/molecules31162882 (PMC13515570; doi:10.3390/molecules31162882)
Supplement: Supplementary file 1 [file molecules-31-02882-s001.zip › Tables_S2-S10_Surface_supplementation.pdf]

Table S2: Growth dynamics of *Heterobasidion parviporum* mycelium measured *in vitro* at 12 h intervals in cultures receiving 0–5 mL of acetone on the surface of solidified Hansen's medium and incubated at 28 °C.

Statistically significant differences compared to the control sample (0 mL) are shown in red.

***Heterobasidion parviporum*; acetone**																										
	Average mycelium diameter [mm] at a given measurement hour ±standard deviation in sample [mm]																									
	12	24	36	48	60	72	84	96	108	120	132	144	156	168	180	192	204	216	228	240	252	264	276	288	300	312
Control sample 0 mL	5 ±0	5,8 ±0,27	7,6 ±0,42	10,7 ±0,57	12,7 ±0,45	15,9 ±0,89	18,3 ±0,84	21,8 ±0,76	24,2 ±1,25	26,4 ±1,14	29,1 ±0,82	32,3 ±1,44	34 ±1	36,4 ±1,56	39 ±1,54	41,3 ±1,35	43,5 ±1,37	46,1 ±1,71	48,7 ±1,3	51,2 ±1,48	53,1 ±1,52	56 ±0,94	57,5 ±1,62	60,6 ±2,01	63 ±1,66	65,3 ±1,92
1 mL acetone	5 ±0	5 ±0	5 ±0	5,8 ±0,27	6,8 ±0,45	7,7 ±0,67	9,1 ±0,82	10,4 ±0,65	11,8 ±1,04	12,8 ±1,48	13,8 ±1,64	15,5 ±1,87	17,1 ±1,75	17,8 ±1,89	19,9 ±1,98	20,7 ±2,11	22,9 ±2,01	24,1 ±2,38	26,2 ±1,64	27,9 ±2,01	29,5 ±1,87	32,1 ±2,19	33,6 ±1,67	36,3 ±1,35	39 ±1,94	40,3 ±1,75
2 mL acetone	5 ±0	5 ±0	5 ±0	5 ±0	5 ±0	5 ±0	5,2 ±0,45	5,7 ±0,84	6,4 ±1,39	6,9 ±1,39	8 ±2,37	9,3 ±2,59	10,8 ±3,37	12 ±3,48	13,5 ±4,23	15 ±4,3	16,8 ±4,83	18,2 ±5,39	20,7 ±5,43	23 ±6,19	25,5 ±6,97	28,1 ±6,98	29,9 ±7,82	33,5 ±8,14	35,9 ±9,02	39,5 ±9,75
3 mL acetone	5 ±0	5 ±0	5 ±0	5 ±0	5 ±0	5 ±0	5 ±0	5 ±0	5 ±0	5 ±0	5 ±0	5 ±0	5 ±0	5 ±0	5 ±0	5 ±0	5 ±0	5 ±0	5,3 ±0,45	5,6 ±0,89	6,5 ±2,06	7,6 ±2,79	8,1 ±3,29	9,1 ±4,02	10,1 ±5,02	12,1 ±6,5
4 mL acetone	5 ±0	5 ±0	5 ±0	5 ±0	5 ±0	5 ±0	5 ±0	5 ±0	5 ±0	5 ±0	5 ±0	5 ±0	5 ±0	5 ±0	5 ±0	5 ±0	5 ±0	5 ±0	5 ±0	5 ±0	5 ±0	5 ±0	5 ±0	5 ±0	5 ±0	5 ±0
5 mL acetone	5 ±0	5 ±0	5 ±0	5 ±0	5 ±0	5 ±0	5 ±0	5 ±0	5 ±0	5 ±0	5 ±0	5 ±0	5 ±0	5 ±0	5 ±0	5 ±0	5 ±0	5 ±0	5 ±0	5 ±0	5 ±0	5 ±0	5 ±0	5 ±0	5 ±0	5 ±0

Table S3: Growth dynamics of *Heterobasidion parviporum* mycelium measured *in vitro* at 12 h intervals in cultures treated with 1 and 2 mL of acetone extract from *Cladonia arbuscula* applied onto the surface of solidified Hansen's medium and incubated at 28 °C.

Statistically significant differences compared to the control sample (0 mL) are shown in red.

Heterobasidion parviporum*; extract from *C. arbuscula																										
	Average mycelium diameter [mm] at a given measurement hour<br>±standard deviation in sample [mm]																									
	12	24	36	48	60	72	84	96	108	120	132	144	156	168	180	192	204	216	228	240	252	264	276	288	300	312
Control sample<br>0 mL	5<br>±0	5,8<br>±0,27	7,6<br>±0,42	10,7<br>±0,57	12,7<br>±0,45	15,9<br>±0,89	18,3<br>±0,84	21,8<br>±0,76	24,2<br>±1,25	26,4<br>±1,14	29,1<br>±0,82	32,3<br>±1,44	34<br>±1	36,4<br>±1,56	39<br>±1,54	41,3<br>±1,35	43,5<br>±1,37	46,1<br>±1,71	48,7<br>±1,3	51,2<br>±1,48	53,1<br>±1,52	56<br>±0,94	57,5<br>±1,62	60,6<br>±2,01	63<br>±1,66	65,3<br>±1,92
1 mL extract	5<br>±0	5<br>±0	5<br>±0	5<br>±0	5<br>±0	5<br>±0	5,3<br>±0,45	5,4<br>±0,55	5,7<br>±0,97	6,2<br>±1,64	6,4<br>±1,95	6,8<br>±2,25	7,3<br>±2,73	8,1<br>±3,29	8,8<br>±3,77	9,6<br>±4,51	10,5<br>±5,15	11,3<br>±5,43	12,3<br>±5,74	13,4<br>±6,32	14,5<br>±7,29	15,4<br>±7,29	16,7<br>±8,12	17,9<br>±8,51	18,9<br>±9,04	20,1<br>±9,63
2 mL extract	5<br>±0	5<br>±0	5<br>±0	5<br>±0	5<br>±0	5<br>±0	5<br>±0	5<br>±0	5,1<br>±0,22	5,2<br>±0,45	5,4<br>±0,65	6,1<br>±0,74	6,5<br>±1,12	6,9<br>±1,29	7,5<br>±1,58	8,8<br>±1,35	9,7<br>±,2	10,7<br>±1,35	11,8<br>±1,57	12,8<br>±1,79	13,9<br>±2,33	15,2<br>±1,82	16,2<br>±1,82	17,4<br>±2,01	18,5<br>±2,35	19,7<br>±2,08

Table S4: Growth dynamics of *Heterobasidion parviporum* mycelium measured *in vitro* at 12 h intervals in cultures treated with 1 and 2 mL of acetone extract from *Cladonia rangiferina* applied onto the surface of solidified Hansen's medium and incubated at 28 °C.

Statistically significant differences compared to the control sample (0 mL) are shown in red.

Heterobasidion parviporum*; extract from *C. rangiferina																										
	Average mycelium diameter [mm] at a given measurement hour ±standard deviation in sample [mm]																									
	12	24	36	48	60	72	84	96	108	120	132	144	156	168	180	192	204	216	228	240	252	264	276	288	300	312
Control sample 0 mL	5 ±0	5,8 ±0,27	7,6 ±0,42	10,7 ±0,57	12,7 ±0,45	15,9 ±0,89	18,3 ±0,84	21,8 ±0,76	24,2 ±1,25	26,4 ±1,14	29,1 ±0,82	32,3 ±1,44	34 ±1	36,4 ±1,56	39 ±1,54	41,3 ±1,35	43,5 ±1,37	46,1 ±1,71	48,7 ±1,3	51,2 ±1,48	53,1 ±1,52	56 ±0,94	57,5 ±1,62	60,6 ±2,01	63 ±1,66	65,3 ±1,92
1 mL extract	5 ±0	5 ±0	5 ±0	5 ±0	5,3 ±0,45	5,9 ±0,89	6,7 ±1,35	7,8 ±1,04	9,1 ±1,29	10,6 ±1,29	11,7 ±1,4	13,4 ±1,71	14,9 ±1,98	17,3 ±2,2	19 ±2,47	21,3 ±2,61	23,5 ±2,78	26,4 ±3,03	28,7 ±3,51	32 ±3,45	34,4 ±4,02	37,6 ±4,38	40,2 ±4,56	43,3 ±4,79	46,5 ±5,04	49,1 ±4,84
2 mL extract	5 ±0	5 ±0	5 ±0	5 ±0	5 ±0	5 ±0	5 ±0	5 ±0	5,1 ±0,22	5,3 ±0,67	5,6 ±1,34	5,8 ±1,79	6 ±2,24	6,4 ±3,13	6,8 ±4,02	7,2 ±4,92	7,6 ±5,81	8 ±6,71	8,3 ±7,28	9 ±8,94	9,5 ±10,06	10,1 ±11,4	10,7 ±12,19	11,6 ±13,67	12,5 ±15,15	13,1 ±16,23

Table S5: Growth dynamics of *Heterobasidion parviporum* mycelium measured *in vitro* at 12 h intervals in cultures treated with 1–3 mL of acetone extract from *Cladonia digitata* applied onto the surface of solidified Hansen's medium and incubated at 28 °C.

Statistically significant differences compared to the control sample (0 mL) are shown in red.

a - statistically significant differences between compared pairs of cultures supplemented with various doses of the extract.

Heterobasidion parviporum*; extract from *C. digitata																										
	Average mycelium diameter [mm] at a given measurement hour ±standard deviation in sample [mm]																									
	12	24	36	48	60	72	84	96	108	120	132	144	156	168	180	192	204	216	228	240	252	264	276	288	300	312
Control sample 0 mL	5 ±0	5,8 ±0,27	7,6 ±0,42	10,7 ±0,57	12,7 ±0,45	15,9 ±0,89	18,3 ±0,84	21,8 ±0,76	24,2 ±1,25	26,4 ±1,14	29,1 ±0,82	32,3 ±1,44	34 ±1	36,4 ±1,56	39 ±1,54	41,3 ±1,35	43,5 ±1,37	46,1 ±1,71	48,7 ±1,3	51,2 ±1,48	53,1 ±1,52	56 ±0,94	57,5 ±1,62	60,6 ±2,01	63 ±1,66	65,3 ±1,92
1 mL extract	5 ±0	5 ±0	5,9 ±0,22	6,8 ±0,84	8,4 ±1,08	10,2 ±1,2	12,6 ±1,14	14,5 ±1,22	16,5 ±1,32	18,6 ±1,47	21,1 ±1,29	22,6 ±1,64	25,3 ±1,86	27,1 ±1,64	30,1 ±2,07	32,1 ±2,07	34,4 ±2,16	36,9 ±2,3	39,7 ±2,64	42,2 ±2,02	44,7[a] ±2,97	48[a] ±3	50,4 ±2,79	53,8[a] ±3,49	55,6 ±3,47	59,5[a] ±3,45
2 mL extract	5 ±0	5 ±0	5 ±0	5 ±0	5 ±0	5 ±0	5 ±0	5 ±0	5 ±0	5 ±0	5,2 ±0,45	5,4 ±0,89	5,6 ±0,89	6,3 ±1,89	7 ±2,42	7,7 ±2,89	9 ±3,59	10,3 ±4,22	11,4 ±4,55	13,4 ±5,58	14,9 ±6,12	17,5 ±7,27	19,3 ±7,64	21,5 ±8,33	23,9 ±9,4	26,9 ±10,44
3 mL extract	5 ±0	5 ±0	5 ±0	5 ±0	5 ±0	5 ±0	5 ±0	5 ±0	5 ±0	5 ±0	5 ±0	5 ±0	5 ±0	5 ±0	5 ±0	5 ±0	5 ±0	5 ±0	5 ±0	5 ±0	5,1[a] ±0,22	5,2[a] ±0,45	5,8 ±1,25	6,6[a] ±2,04	7,6 ±2,72	8,9[a] ±4,16

Table S6: Growth dynamics of *Heterobasidion parviporum* mycelium measured *in vitro* at 12 h intervals in cultures treated with 1–3 mL of acetone extract from *Cladonia uncialis* applied onto the surface of solidified Hansen's medium and incubated at 28 °C.

Statistically significant differences compared to the control sample (0 mL) are shown in red.

Heterobasidion parviporum*; extract from *C. uncialis																										
	Average mycelium diameter [mm] at a given measurement hour ±standard deviation in sample [mm]																									
	12	24	36	48	60	72	84	96	108	120	132	144	156	168	180	192	204	216	228	240	252	264	276	288	300	312
Control sample 0 mL	5 ±0	5,8 ±0,27	7,6 ±0,42	10,7 ±0,57	12,7 ±0,45	15,9 ±0,89	18,3 ±0,84	21,8 ±0,76	24,2 ±1,25	26,4 ±1,14	29,1 ±0,82	32,3 ±1,44	34 ±1	36,4 ±1,56	39 ±1,54	41,3 ±1,35	43,5 ±1,37	46,1 ±1,71	48,7 ±1,3	51,2 ±1,48	53,1 ±1,52	56 ±0,94	57,5 ±1,62	60,6 ±2,01	63 ±1,66	65,3 ±1,92
1 mL extract	5 ±0	5 ±0	5 ±0	5 ±0	5 ±0	5 ±0	5 ±0	5 ±0	5 ±0	5,2 ±0,45	5,2 ±0,45	5,4 ±0,89	5,6 ±1,34	5,6 ±1,34	5,8 ±1,79	5,8 ±1,79	6,3 ±2,64	6,6 ±3,05	7,2 ±4,13	7,7 ±4,76	8,6 ±6,07	9,3 ±6,96	10 ±7,71	10,9 ±8,89	11,4 ±9,74	12,3 ±10,79
2 mL extract	5 ±0	5 ±0	5 ±0	5 ±0	5 ±0	5 ±0	5 ±0	5 ±0	5 ±0	5 ±0	5 ±0	5 ±0	5 ±0	5 ±0	5 ±0	5 ±0	5 ±0	5 ±0	5 ±0	5 ±0	5 ±0	5 ±0	5 ±0	5 ±0	5 ±0	5 ±0
3 mL extract	5 ±0	5 ±0	5 ±0	5 ±0	5 ±0	5 ±0	5 ±0	5 ±0	5 ±0	5 ±0	5 ±0	5 ±0	5 ±0	5 ±0	5 ±0	5 ±0	5 ±0	5 ±0	5 ±0	5 ±0	5 ±0	5 ±0	5 ±0	5 ±0	5 ±0	5 ±0

Table S7: Growth dynamics of *Heterobasidion parviporum* mycelium measured *in vitro* at 12 h intervals in cultures treated with 1–3 mL of acetone extract from *Cetraria islandica* applied onto the surface of solidified Hansen's medium and incubated at 28 °C.

Statistically significant differences compared to the control sample (0 mL) are shown in red.

Heterobasidion parviporum*; extract from *C. islandica																										
	Average mycelium diameter [mm] at a given measurement hour<br>±standard deviation in sample [mm]																									
	12	24	36	48	60	72	84	96	108	120	132	144	156	168	180	192	204	216	228	240	252	264	276	288	300	312
Control sample<br>0 mL	5<br>±0	5,8<br>±0,27	7,6<br>±0,42	10,7<br>±0,57	12,7<br>±0,45	15,9<br>±0,89	18,3<br>±0,84	21,8<br>±0,76	24,2<br>±1,25	26,4<br>±1,14	29,1<br>±0,82	32,3<br>±1,44	34<br>±1	36,4<br>±1,56	39<br>±1,54	41,3<br>±1,35	43,5<br>±1,37	46,1<br>±1,71	48,7<br>±1,3	51,2<br>±1,48	53,1<br>±1,52	56<br>±0,94	57,5<br>±1,62	60,6<br>±2,01	63<br>±1,66	65,3<br>±1,92
1 mL extract	5<br>±0	5<br>±0	5<br>±0	5<br>±0	5<br>±0	5<br>±0	5<br>±0	5<br>±0	5<br>±0	5<br>±0	5<br>±0	5<br>±0	5<br>±0	5<br>±0	5<br>±0	5<br>±0	5<br>±0	5<br>±0	5<br>±0	5<br>±0	5<br>±0	5<br>±0	5<br>±0	5<br>±0	5<br>±0	5<br>±0
2 mL extract	5<br>±0	5<br>±0	5<br>±0	5<br>±0	5<br>±0	5<br>±0	5<br>±0	5<br>±0	5<br>±0	5<br>±0	5<br>±0	5<br>±0	5<br>±0	5<br>±0	5<br>±0	5<br>±0	5<br>±0	5<br>±0	5<br>±0	5<br>±0	5<br>±0	5<br>±0	5<br>±0	5<br>±0	5<br>±0	5<br>±0
3 mL extract	5<br>±0	5<br>±0	5<br>±0	5<br>±0	5<br>±0	5<br>±0	5<br>±0	5<br>±0	5<br>±0	5<br>±0	5<br>±0	5<br>±0	5<br>±0	5<br>±0	5<br>±0	5<br>±0	5<br>±0	5<br>±0	5<br>±0	5<br>±0	5<br>±0	5<br>±0	5<br>±0	5<br>±0	5<br>±0	5<br>±0

Table S8: Growth dynamics of *Heterobasidion parviporum* mycelium measured *in vitro* at 12 h intervals in cultures treated with 1–3 mL of acetone extract prepared from mixed thalli of *Cladonia arbuscula* + *Cladonia rangiferina* + *Cladonia digitata*, applied onto the surface of solidified Hansen's medium and incubated at 28 °C.

Statistically significant differences compared to the control sample (0 mL) are shown in red.

Heterobasidion parviporum*; extract from *Cladonia arbuscula* + *C. rangiferina* + *C. digitata																										
	Average mycelium diameter [mm] at a given measurement hour ±standard deviation in sample [mm]																									
	12	24	36	48	60	72	84	96	108	120	132	144	156	168	180	192	204	216	228	240	252	264	276	288	300	312
Control sample 0 mL	5 ±0	5,8 ±0,27	7,6 ±0,42	10,7 ±0,57	12,7 ±0,45	15,9 ±0,89	18,3 ±0,84	21,8 ±0,76	24,2 ±1,25	26,4 ±1,14	29,1 ±0,82	32,3 ±1,44	34 ±1	36,4 ±1,56	39 ±1,54	41,3 ±1,35	43,5 ±1,37	46,1 ±1,71	48,7 ±1,3	51,2 ±1,48	53,1 ±1,52	56 ±0,94	57,5 ±1,62	60,6 ±2,01	63 ±1,66	65,3 ±1,92
1 mL extract	5 ±0	5 ±0	5,4 ±0,55	6,2 ±1,44	7,4 ±1,98	8,4 ±2,41	9,7 ±2,59	10,5 ±3,67	11,4 ±4,29	12,4 ±4,89	13,3 ±5,69	13,9 ±6,07	14,9 ±7,22	15,5 ±8,07	16,5 ±8,76	17,4 ±9,48	18,2 ±10,37	19 ±11,25	20,3 ±12,3	21,6 ±13,18	22,4 ±13,81	23,8 ±15,28	25,3 ±15,96	26,6 ±16,82	27,8 ±17,54	29,6 ±19,12
2 mL extract	5 ±0	5 ±0	5 ±0	5 ±0	5 ±0	5 ±0	5 ±0	5 ±0	5 ±0	5 ±0	5,1 ±0,22	5,3 ±0,67	5,4 ±0,89	5,4 ±0,89	5,6 ±1,34	5,8 ±1,3	6 ±1,54	6 ±1,54	6,2 ±1,68	6,6 ±1,78	7 ±1,77	7,3 ±2,08	7,6 ±1,98	8,1 ±2,33	8,2 ±2,33	8,3 ±2,36
3 mL extract	5 ±0	5 ±0	5 ±0	5 ±0	5 ±0	5 ±0	5 ±0	5 ±0	5 ±0	5 ±0	5 ±0	5 ±0	5 ±0	5 ±0	5 ±0	5 ±0	5 ±0	5 ±0	5 ±0	5 ±0	5 ±0	5 ±0	5 ±0	5 ±0	5 ±0	5 ±0

Table S9: Growth dynamics of *Heterobasidion parviporum* mycelium measured *in vitro* at 12 h intervals in cultures treated with 1–3 mL of acetone extract prepared from mixed thalli of *Cladonia arbuscula* + *Cladonia rangiferina* + *Cladonia digitata* + *Cladonia uncialis* + *Cetraria islandica*, applied onto the surface of solidified Hansen's medium and incubated at 28 °C.

Statistically significant differences compared to the control sample (0 mL) are shown in red.

Heterobasidion parviporum*; extract from *Cladonia arbuscula* + *C. rangiferina* + *C. digitata* + *C. uncialis* + *Cetraria islandica																										
	Average mycelium diameter [mm] at a given measurement hour ±standard deviation in sample [mm]																									
	12	24	36	48	60	72	84	96	108	120	132	144	156	168	180	192	204	216	228	240	252	264	276	288	300	312
Control sample 0 mL	5 ±0	5,8 ±0,27	7,6 ±0,42	10,7 ±0,57	12,7 ±0,45	15,9 ±0,89	18,3 ±0,84	21,8 ±0,76	24,2 ±1,25	26,4 ±1,14	29,1 ±0,82	32,3 ±1,44	34 ±1	36,4 ±1,56	39 ±1,54	41,3 ±1,35	43,5 ±1,37	46,1 ±1,71	48,7 ±1,3	51,2 ±1,48	53,1 ±1,52	56 ±0,94	57,5 ±1,62	60,6 ±2,01	63 ±1,66	65,3 ±1,92
1 mL extract	5 ±0	5 ±0	5 ±0	5 ±0	5 ±0	5 ±0	5 ±0	5 ±0	5 ±0	5 ±0	5 ±0	5 ±0	5 ±0	5 ±0	5 ±0	5 ±0	5 ±0	5 ±0	5 ±0	5 ±0	5 ±0	5 ±0	5 ±0	5 ±0	5 ±0	5 ±0
2 mL extract	5 ±0	5 ±0	5 ±0	5 ±0	5 ±0	5 ±0	5 ±0	5 ±0	5 ±0	5 ±0	5 ±0	5 ±0	5 ±0	5 ±0	5 ±0	5 ±0	5 ±0	5 ±0	5 ±0	5 ±0	5 ±0	5 ±0	5 ±0	5 ±0	5 ±0	5 ±0
3 mL extract	5 ±0	5 ±0	5 ±0	5 ±0	5 ±0	5 ±0	5 ±0	5 ±0	5 ±0	5 ±0	5 ±0	5 ±0	5 ±0	5 ±0	5 ±0	5 ±0	5 ±0	5 ±0	5 ±0	5 ±0	5 ±0	5 ±0	5 ±0	5 ±0	5 ±0	5 ±0

Table S10: Growth dynamics of *Heterobasidion parviporum* mycelium measured *in vitro* at 12 h intervals in cultures treated with 1–3 mL of acetone extract from *Pseudevernia furfuracea* applied onto the surface of solidified Hansen's medium and incubated at 28 °C.

Statistically significant differences compared to the control sample (0 mL) are shown in red.

Heterobasidion parviporum*; extract from *P. furfuracea																										
	Average mycelium diameter [mm] at a given measurement hour ±standard deviation in sample [mm]																									
	12	24	36	48	60	72	84	96	108	120	132	144	156	168	180	192	204	216	228	240	252	264	276	288	300	312
Control sample 0 mL	5 ±0	5,8 ±0,27	7,6 ±0,42	10,7 ±0,57	12,7 ±0,45	15,9 ±0,89	18,3 ±0,84	21,8 ±0,76	24,2 ±1,25	26,4 ±1,14	29,1 ±0,82	32,3 ±1,44	34 ±1	36,4 ±1,56	39 ±1,54	41,3 ±1,35	43,5 ±1,37	46,1 ±1,71	48,7 ±1,3	51,2 ±1,48	53,1 ±1,52	56 ±0,94	57,5 ±1,62	60,6 ±2,01	63 ±1,66	65,3 ±1,92
1 mL extract	5 ±0	5 ±0	5 ±0	5 ±0	5 ±0	5 ±0	5 ±0	5 ±0	5 ±0	5 ±0	5 ±0	5 ±0	5 ±0	5 ±0	5 ±0	5 ±0	5 ±0	5 ±0	5 ±0	5 ±0	5 ±0	5 ±0	5 ±0	5 ±0	5 ±0	5 ±0
2 mL extract	5 ±0	5 ±0	5 ±0	5 ±0	5 ±0	5 ±0	5 ±0	5 ±0	5 ±0	5 ±0	5 ±0	5 ±0	5 ±0	5 ±0	5 ±0	5 ±0	5 ±0	5 ±0	5 ±0	5 ±0	5 ±0	5 ±0	5 ±0	5 ±0	5 ±0	5 ±0
3 mL extract	5 ±0	5 ±0	5 ±0	5 ±0	5 ±0	5 ±0	5 ±0	5 ±0	5 ±0	5 ±0	5 ±0	5 ±0	5 ±0	5 ±0	5 ±0	5 ±0	5 ±0	5 ±0	5 ±0	5 ±0	5 ±0	5 ±0	5 ±0	5 ±0	5 ±0	5 ±0
