# Supplementary material for: Antifungal and Profungal Potentials of Acetone-Extracted Complexes of Lichen Substances on Macromycete Heterobasidion parviporum Mycelium Growth Dynamics—The First In Vitro Approach
Source: Molecules. 2026 Aug 18;31(16):2882. doi: 10.3390/molecules31162882 (PMC13515570; doi:10.3390/molecules31162882)
Supplement: Supplementary file 1 [file molecules-31-02882-s001.zip › Tables_S11-S19_Medium_incorporation.pdf]

Table S11: Growth dynamics of *Heterobasidion parviporum* mycelium measured *in vitro* at 12 h intervals in cultures receiving 0–4 mL of acetone incorporated into liquid Hansen's medium before solidification and incubated at 28 °C.

[a, b] - statistically significant differences between compared pairs of cultures supplemented with various doses of acetone.

***Heterobasidion parviporum*; acetone**																			
	Average mycelium diameter [mm] at a given measurement hour ±standard deviation in sample [mm]																		
	12	24	36	48	60	72	84	96	108	120	132	144	156	168	180	192	204	216	228
Control sample 0 mL	5 ±0	5 ±0	5,5 ±0,87	7,8 ±1,35	10,5 ±1,12	12,9 ±1,67	15,8 ±2,02	19 ±2,26	22,5 ±2,69	26,3 ±2,89	29,5 ±3,22	33,3 ±3,65	37,5 ±4,09	40,9 ±4,26	45,5 ±4,97	49,9 ±5,07	53,6 ±5,96	58,4 ±5,91	62,4 ±6,1
1 mL acetone	5 ±0	5 ±0	5[a] ±0	7[a] ±0,94	8,9[a] ±1,02	11,5[a] ±0,87	13,3[a] ±1,6	15,7[a] ±2,2	18,7[a] ±2,59	21,6[a] ±3,09	24,7[a] ±3,78	27,8[a] ±4,09	31,5[a] ±4,68	35,2[a] ±4,83	39,3[a] ±5,51	43,4 ±6,46	46,6 ±6,09	50,5[a] ±6,05	54,7 ±5,85
2 mL acetone	5 ±0	5 ±0	6 ±0,35	8,4 ±0,65	10,8 ±0,57	14,1 ±0,55	16,3 ±0,45	19,8 ±1,25	23 ±1,66	26,1 ±1,14	29,7 ±1,35	33 ±1,58	37,4 ±2,04	40,4 ±1,78	44,9 ±2,51	48,7 ±2,36	52,1 ±2,48	56,7 ±2,56	61 ±2,89
3 mL acetone	5 ±0	5 ±0	7,2[a] ±0,27	10[a] ±0	12,2[a, b] ±0,45	15,8[a, b] ±0,27	19[a, b] ±0,94	21,7[a, b] ±0,91	25,1[a, b] ±0,74	28,9[a, b] ±1,14	32,5[a] ±1,06	36,4[a, b] ±1,24	40,3[a] ±1,1	43,6[a] ±1,56	48,5[a] ±1,27	52,2 ±1,99	55,5 ±2,03	59,7[a] ±1,48	63,8 ±1,64
4 mL acetone	5 ±0	5 ±0	5,8 ±0,84	7,3 ±1,72	9,5[b] ±1,62	11,8[b] ±2,14	13,6[b] ±2,68	15,9[b] ±3,36	19[b] ±4,23	21,5[b] ±4,78	25,3 ±5,82	28,2[b] ±6,19	32,2 ±6,87	35,7 ±7,16	39,7 ±7,06	43,6 ±8,12	46,9 ±7,82	51,1 ±7,51	55,8 ±8,08

Table S12: Growth dynamics of *Heterobasidion parviporum* mycelium measured *in vitro* at 12 h intervals in cultures treated with 1–4 mL of acetone extract from *Cladonia arbuscula* incorporated into liquid Hansen's medium before solidification and incubated at 28 °C.

Statistically significant differences compared to the control sample (0 mL) are shown in red.

a - statistically significant differences between compared pairs of cultures supplemented with various doses of the extract.

Heterobasidion parviporum*; extract from *C. arbuscula																			
	Average mycelium diameter [mm] at a given measurement hour ±standard deviation in sample [mm]																		
	12	24	36	48	60	72	84	96	108	120	132	144	156	168	180	192	204	216	228
Control sample 0 mL	5 ±0	5 ±0	5,5 ±0,87	7,8 ±1,35	10,5 ±1,12	12,9 ±1,67	15,8 ±2,02	19 ±2,26	22,5 ±2,69	26,3 ±2,89	29,5 ±3,22	33,3 ±3,65	37,5 ±4,09	40,9 ±4,26	45,5 ±4,97	49,9 ±5,07	53,6 ±5,96	58,4 ±5,91	62,4 ±6,1
1 mL extract	5 ±0	5 ±0	5 ±0	5,7 ±0,67	6,8 ±1,04	8,6[a] ±1,19	11,1 ±1,43	12,9 ±1,34	15,8 ±2,33	17,5 ±2,81	20,5[a] ±2,96	22,3 ±3,91	24,8 ±4,86	26,9 ±6,07	28,4 ±7	31,2 ±7,46	33,4 ±7,85	35,2 ±7,83	38,1 ±8,88
2 mL extract	5 ±0	5 ±0	5 ±0	5 ±0	5,5 ±0,5	6,5 ±0,35	7,8 ±0,27	10,3 ±0,45	11,4 ±0,74	13,9 ±0,89	15,4 ±1,85	17,1 ±2,3	19,1 ±3,17	20,9 ±3,97	22,9 ±4,75	24,5 ±5,62	26,3 ±6,38	28,3 ±7,22	30,3 ±8,3
3 mL extract	5 ±0	5 ±0	5 ±0	5 ±0	5 ±0	5,6 ±0,65	6,4 ±0,65	8,2 ±1,15	10 ±0,5	11,7 ±0,57	14,3 ±1,2	15,7 ±0,84	17,7 ±0,91	19,6 ±0,82	21,1 ±1,14	23,3 ±1,6	25,1 ±1,78	27 ±2,03	29,6 ±2,33
4 mL extract	5 ±0	5 ±0	5 ±0	5 ±0	5 ±0	5,3[a] ±0,45	6,4 ±1,24	7,9 ±1,29	9,3 ±1,64	11 ±1,41	13[a] ±1,58	14,5 ±1,87	16,4 ±2,86	17,7 ±2,97	19,4 ±3,07	20,7 ±3,77	22,6 ±4,36	23,8 ±4,31	26,5 ±5,09

Table S13: Growth dynamics of *Heterobasidion parviporum* mycelium measured *in vitro* at 12 h intervals in cultures treated with 1–4 mL of acetone extract from *Cladonia rangiferina* incorporated into liquid Hansen's medium before solidification and incubated at 28 °C.

Statistically significant differences compared to the control sample (0 mL) are shown in red.

a, b - statistically significant differences between compared pairs of cultures supplemented with various doses of the extract.

Heterobasidion parviporum*; extract from *C. rangiferina																			
	Average mycelium diameter [mm] at a given measurement hour ±standard deviation in sample [mm]																		
	**12**	**24**	**36**	**48**	**60**	**72**	**84**	**96**	**108**	**120**	**132**	**144**	**156**	**168**	**180**	**192**	**204**	**216**	**228**
Control sample 0 mL	5 ±0	5 ±0	5,5 ±0,87	7,8 ±1,35	10,5 ±1,12	12,9 ±1,67	15,8 ±2,02	19 ±2,26	22,5 ±2,69	26,3 ±2,89	29,5 ±3,22	33,3 ±3,65	37,5 ±4,09	40,9 ±4,26	45,5 ±4,97	49,9 ±5,07	53,6 ±5,96	58,4 ±5,91	62,4 ±6,1
1 mL extract	5 ±0	5 ±0	6,4 ±0,55	8,5 ±0,61	10,8 ±0,45	13,4 ±0,82	16,6 ±0,82	19,7 ±1,35	23,5 ±1,12	26,4 ±1,29	30,2 ±1,68	34,8[b] ±1,3	38,5[b] ±2,03	41,5[b] ±1,66	45,4[b] ±1,64	49,4[b] ±1,64	53,3[b] ±1,6	57,7[b] ±1,6	61,7[b] ±1,15
2 mL extract	5 ±0	5,3 ±0,27	8,1 ±0,74	10,4[a] ±0,42	13,5[a] ±0,5	16,4[a] ±0,42	19,2[a] ±0,91	22,7[a] ±0,97	25,8[a] ±0,76	29,4[a] ±0,82	33,2[a] ±1,2	36[a] ±1	40,3[a] ±1,04	43,2[a] ±1,04	47,1[a] ±0,96	50,5[a] ±0,94	53,8[a] ±0,97	57,5[a] ±1,7	61,1[a] ±0,82
3 mL extract	5 ±0	5 ±0	6,7 ±0,76	9,7 ±0,45	12 ±0,94	14,6 ±0,74	17,3 ±1,15	20,5 ±1	23,2 ±0,91	26,1 ±1,02	29,7 ±1,1	32,9 ±1,29	36,9 ±1,08	39,6 ±1,08	42,5 ±0,79	45,9 ±1,64	49,4 ±1,71	52,9 ±0,74	57 ±1,66
4 mL extract	5 ±0	5 ±0	6 ±0,79	7,9[a] ±0,74	9,7[a] ±0,76	11,3[a] ±0,97	13,6[a] ±0,89	16[a] ±0,79	18,4[a] ±1,6	21,8[a] ±1,35	24,5[a] ±1,46	27,2[a, b] ±1,64	31,1[a, b] ±2,01	33,9[a, b] ±1,82	37,4[a, b] ±1,98	40,8[a, b] ±2,17	45[a, b] ±2,4	49,1[a, b] ±2,68	52,4[a, b] ±2,97

Table S14: Growth dynamics of *Heterobasidion parviporum* mycelium measured *in vitro* at 12 h intervals in cultures treated with 1–4 mL of acetone extract from *Cladonia digitata* incorporated into liquid Hansen's medium before solidification and incubated at 28 °C.

a - statistically significant differences between compared pairs of cultures supplemented with various doses of the extract.

Heterobasidion parviporum*; extract from *C. digitata																			
	Average mycelium diameter [mm] at a given measurement hour ±standard deviation in sample [mm]																		
	12	24	36	48	60	72	84	96	108	120	132	144	156	168	180	192	204	216	228
Control sample 0 mL	5 ±0	5 ±0	5,5 ±0,87	7,8 ±1,35	10,5 ±1,12	12,9 ±1,67	15,8 ±2,02	19 ±2,26	22,5 ±2,69	26,3 ±2,89	29,5 ±3,22	33,3 ±3,65	37,5 ±4,09	40,9 ±4,26	45,5 ±4,97	49,9 ±5,07	53,6 ±5,96	58,4 ±5,91	62,4 ±6,1
1 mL extract	5 ±0	5 ±0	6,7 ±0,67	8,8 ±1,44	11,1 ±1,56	13,7 ±1,64	16,3 ±2,08	19,4 ±2,95	22,3 ±3,38	25,6 ±4,17	28,7 ±4,55	32,4 ±5,28	35,8 ±5,84	40,3 ±6,05	42,3 ±6,26	46,9 ±7,12	51,1 ±7,44	54,5 ±7,88	58,5 ±8,76
2 mL extract	5 ±0	5 ±0	7,1 ±0,22	9,2 ±0,45	12,4[a] ±0,89	15,4 ±0,82	18,2[a] ±1,44	21,7 ±1,35	25,2[a] ±1,92	28,5[a] ±1,62	32,1 ±1,82	35,7 ±1,82	38,4 ±2,16	42,7 ±2,02	45,8 ±2,33	49,5 ±1,46	53,1 ±1,24	56,2 ±1,04	61 ±1,46
3 mL extract	5 ±0	5,2 ±0,45	6,5 ±0,5	8,2 ±0,27	9,9[a] ±0,55	12,7 ±1,04	14,5[a] ±1,06	17,7 ±1,35	19,8[a] ±1,35	23,1[a] ±1,52	26,3 ±2,66	30,2 ±3,09	33,9 ±3,27	37,2 ±3,68	40,3 ±2,86	44,7 ±2,97	48,8 ±2,66	52,5 ±2,55	56,8 ±2,97
4 mL extract	5 ±0	5 ±0	6,4 ±0,96	8,2 ±1,64	10,7 ±2,2	13,6 ±2,68	15,5 ±3,24	18 ±3,55	21,1 ±3,94	23,7 ±4,74	26,5 ±5,76	30,4 ±6,38	32,5 ±7,22	36,2 ±8,42	38,2 ±9,28	42,1 ±10,84	45,6 ±10,84	48 ±12,17	52 ±12,16

Table S15: Growth dynamics of *Heterobasidion parviporum* mycelium measured *in vitro* at 12 h intervals in cultures treated with 1–4 mL of acetone extract from *Cladonia uncialis* incorporated into liquid Hansen's medium before solidification and incubated at 28 °C.

Statistically significant differences compared to the control sample (0 mL) are shown in red.

Heterobasidion parviporum*; extract from *C. uncialis																			
	Average mycelium diameter [mm] at a given measurement hour ±standard deviation in sample [mm]																		
	12	24	36	48	60	72	84	96	108	120	132	144	156	168	180	192	204	216	228
Control sample 0 mL	5 ±0	5 ±0	5,5 ±0,87	7,8 ±1,35	10,5 ±1,12	12,9 ±1,67	15,8 ±2,02	19 ±2,26	22,5 ±2,69	26,3 ±2,89	29,5 ±3,22	33,3 ±3,65	37,5 ±4,09	40,9 ±4,26	45,5 ±4,97	49,9 ±5,07	53,6 ±5,96	58,4 ±5,91	62,4 ±6,1
1 mL extract	5 ±0	5 ±0	5,6 ±0,55	7,3 ±1,68	8,6 ±2,33	10,5 ±3,32	12,1 ±4,16	14,2 ±5,39	16 ±6,32	17,6 ±7,22	20,1 ±8,73	22,5 ±9,94	24,6 ±11,22	26,8 ±12,34	28,5 ±13,22	30,8 ±14,71	33 ±15,95	35,9 ±17,71	38,4 ±19,11
2 mL extract	5 ±0	5 ±0	5 ±0	5,9 ±0,22	7,5 ±0,5	9,5 ±0,5	10,9 ±0,65	12,5 ±0,94	14,7 ±0,91	16,7 ±0,84	19,4 ±0,65	21 ±0,35	23,6 ±1,14	25,1 ±0,96	27,8 ±1,2	29,1 ±0,96	31,3 ±1,25	33,6 ±1,39	36,2 ±1,25
3 mL extract	5 ±0	5 ±0	5 ±0	5,4 ±0,55	6,6 ±0,65	7,6 ±0,96	8,9 ±1,52	10,4 ±1,24	12,1 ±1,14	14,5 ±1,17	16,8 ±0,91	18,5 ±1,77	19,9 ±1,39	21,8 ±1,1	24,1 ±1,08	26 ±1,84	27,6 ±0,89	29,9 ±1,29	32,1 ±1,56
4 mL extract	5 ±0	5 ±0	5 ±0	5 ±0	5,1 ±0,22	5,6 ±0,55	6,5 ±1	7,3 ±1,57	8,3 ±1,89	9,6 ±2,58	11,4 ±3,66	12,6 ±4,44	13,8 ±5,08	14,9 ±5,57	16 ±6,17	17,9 ±7,34	19,2 ±8,04	20,6 ±8,81	22,6 ±9,96

Table S16: Growth dynamics of *Heterobasidion parviporum* mycelium measured *in vitro* at 12 h intervals in cultures treated with 1–4 mL of acetone extract from *Cetraria islandica* incorporated into liquid Hansen's medium before solidification and incubated at 28 °C.

a - statistically significant differences between compared pairs of cultures supplemented with various doses of the extract.

Heterobasidion parviporum*; extract from *C. islandica																			
	Average mycelium diameter [mm] at a given measurement hour ±standard deviation in sample [mm]																		
	12	24	36	48	60	72	84	96	108	120	132	144	156	168	180	192	204	216	228
Control sample 0 mL	5 ±0	5 ±0	5,5 ±0,87	7,8 ±1,35	10,5 ±1,12	12,9 ±1,67	15,8 ±2,02	19 ±2,26	22,5 ±2,69	26,3 ±2,89	29,5 ±3,22	33,3 ±3,65	37,5 ±4,09	40,9 ±4,26	45,5 ±4,97	49,9 ±5,07	53,6 ±5,96	58,4 ±5,91	62,4 ±6,1
1 mL extract	5 ±0	5 ±0	6,3 ±0,67	9,3 ±1,4	11,4 ±2,04	15,3 ±2,97	18,7 ±3,68	22,4 ±4,11	26,4 ±4,35	30 ±4,81	34,7 ±5,31	37,5 ±4,96	41,8 ±5,03	45,8 ±5,19	49,6 ±4,79	53,4 ±5,76	58,1 ±5,27	62[a] ±5,44	66,4 ±4,84
2 mL extract	5 ±0	5,1 ±0,22	6,9 ±0,55	9,7 ±1,25	12,2 ±1,44	15,8 ±2,31	19,1 ±2,7	22,3 ±3,19	25,8 ±3,33	29 ±3,48	33,2 ±3,63	35,9 ±3,68	39,9 ±3,13	43,1 ±3,76	46,8 ±3,29	49,7 ±3,27	53,1 ±2,9	56,3 ±3,05	60,2 ±2,39
3 mL extract	5 ±0	5,2 ±0,45	7,2 ±0,27	10,2 ±0,45	12,6 ±0,42	16,5 ±0,94	20,1 ±1,08	23,3 ±1,15	26,7 ±1,15	29,7 ±1,2	33,6 ±1,39	36 ±1,41	39,6 ±1,64	42,6 ±1,64	46 ±1,84	49,2 ±2,02	51,7 ±1,6	55,1 ±1,98	58,4 ±2,43
4 mL extract	5 ±0	5,1 ±0,22	6,8 ±1,15	9,1 ±2,36	11 ±3,54	13,9 ±5,14	16,2 ±6,43	18,9 ±7,86	21,4 ±9,3	23,8 ±10,62	26,7 ±12,29	28,9 ±13,44	31,7 ±15,02	34,1 ±16,35	37,1 ±18	39,4 ±19,29	41,8 ±20,64	44,3[a] ±22,02	46,6 ±23,31

Table S17: Growth dynamics of *Heterobasidion parviporum* mycelium measured *in vitro* at 12 h intervals in cultures treated with 1–4 mL of acetone extract prepared from mixed thalli of *Cladonia arbuscula* + *Cladonia rangiferina* + *Cladonia digitata*, incorporated into liquid Hansen's medium before solidification and incubated at 28 °C.

Statistically significant differences compared to the control sample (0 mL) are shown in red.

a - statistically significant differences between compared pairs of cultures supplemented with various doses of the extract.

Heterobasidion parviporum*; extract from *Cladonia arbuscula* + *C. rangiferina* + *C. digitata																			
	Average mycelium diameter [mm] at a given measurement hour ±standard deviation in sample [mm]																		
	12	24	36	48	60	72	84	96	108	120	132	144	156	168	180	192	204	216	228
Control sample 0 mL	5 ±0	5 ±0	5,5 ±0,87	7,8 ±1,35	10,5 ±1,12	12,9 ±1,67	15,8 ±2,02	19 ±2,26	22,5 ±2,69	26,3 ±2,89	29,5 ±3,22	33,3 ±3,65	37,5 ±4,09	40,9 ±4,26	45,5 ±4,97	49,9 ±5,07	53,6 ±5,96	58,4 ±5,91	62,4 ±6,1
1 mL extract	5 ±0	5 ±0	5,9 ±0,22	7,1 ±0,74	9[a] ±0,35	11 ±0,71	12,6 ±1,24	14,9 ±1,67	16,9 ±1,24	19,5 ±1,58	22 ±1,22	24,6 ±1,08	27,3 ±0,91	30 ±1,77	31,5 ±0,79	34,8 ±1,04	36,7 ±0,76	38,7 ±0,97	41 ±0,61
2 mL extract	5 ±0	5 ±0	5 ±0	5,5 ±0,5	7 ±0,94	8,6 ±0,42	9,9 ±0,65	11,6 ±0,74	13,1 ±0,96	14,6 ±0,65	17,2 ±0,76	19,1 ±1,34	21 ±1,12	22,8 ±1,44	24,8 ±1,04	27,1 ±1,95	28,9 ±1,82	30,5 ±1,66	33 ±1,58
3 mL extract	5 ±0	5 ±0	5 ±0	5,2 ±0,45	6,6 ±0,82	8,4 ±0,82	10 ±0,94	11,5 ±0,71	12,9 ±0,96	14,9 ±0,74	16,7 ±1,15	19 ±1,62	20 ±1,77	22,6 ±2,51	24,6 ±2,38	26,1 ±2,1	28,4 ±1,92	29,7 ±2,22	31,5 ±2,4
4 mL extract	5 ±0	5 ±0	5 ±0	5,1 ±0,22	5,6[a] ±0,65	7,4 ±1,52	8,7 ±2,31	10,6 ±2,72	12,2 ±2,91	14 ±2,57	15,6 ±2,88	17,6 ±2,63	18,9 ±2,33	21,5 ±2,67	22,6 ±2,95	25,2 ±2,56	27,2 ±2,08	28,5 ±2,76	30,8 ±2,68

Table S18: Growth dynamics of *Heterobasidion parviporum* mycelium measured *in vitro* at 12 h intervals in cultures treated with 1–4 mL of acetone extract prepared from mixed thalli of *Cladonia arbuscula* + *Cladonia rangiferina* + *Cladonia digitata* + *Cladonia uncialis* + *Cetraria islandica*, incorporated into liquid Hansen's medium before solidification and incubated at 28 °C.

Statistically significant differences compared to the control sample (0 mL) are shown in red.

a - statistically significant differences between compared pairs of cultures supplemented with various doses of the extract.

Heterobasidion parviporum*; extract from *Cladonia arbuscula* + *C. rangiferina* + *C. digitata* + *C. uncialis* + *Cetraria islandica																			
	Average mycelium diameter [mm] at a given measurement hour ±standard deviation in sample [mm]																		
	12	24	36	48	60	72	84	96	108	120	132	144	156	168	180	192	204	216	228
Control sample 0 mL	5<br>±0	5<br>±0	5,5<br>±0,87	7,8<br>±1,35	10,5<br>±1,12	12,9<br>±1,67	15,8<br>±2,02	19<br>±2,26	22,5<br>±2,69	26,3<br>±2,89	29,5<br>±3,22	33,3<br>±3,65	37,5<br>±4,09	40,9<br>±4,26	45,5<br>±4,97	49,9<br>±5,07	53,6<br>±5,96	58,4<br>±5,91	62,4<br>±6,1
1 mL extract	5<br>±0	5<br>±0	5<br>±0	6,7<br>±0,45	8,7[a]<br>±0,45	10,5[a]<br>±0,5	12,7[a]<br>±0,45	14,9[a]<br>±0,42	17,3[a]<br>±0,57	20,1[a]<br>±0,42	22,3[a]<br>±1,2	25[a]<br>±0,79	27,9[a]<br>±0,96	30,1[a]<br>±0,74	32,9[a]<br>±1,56	35,8[a]<br>±1,75	38,5<br>±2,06	42,2[a]<br>±2,59	43,4[a]<br>±2,16
2 mL extract	5<br>±0	5<br>±0	5<br>±0	5,9<br>±0,55	7,9<br>±0,55	9,3<br>±0,97	11<br>±1,41	12,8<br>±1,75	15,1<br>±1,75	16,8<br>±1,99	18,7<br>±1,72	21,5<br>±2,74	24,1<br>±2,48	26,3<br>±2,71	29,6<br>±2,95	31,3<br>±3,01	34,5<br>±3,52	36,4<br>±3,7	38,5<br>±3,1
3 mL extract	5<br>±0	5<br>±0	5<br>±0	5<br>±0	6,6<br>±0,55	8,4<br>±0,22	9,8<br>±0,27	11,3<br>±0,57	12,9<br>±0,74	14,9<br>±0,65	16,3<br>±0,76	19,1<br>±0,89	20,7<br>±0,97	22,3<br>±1,44	24,5<br>±2,09	26,1<br>±1,71	27,9<br>±1,47	29,9<br>±1,88	31,9<br>±2,38
4 mL extract	5<br>±0	5<br>±0	5<br>±0	5<br>±0	5,9[a]<br>±0,55	6,9[a]<br>±1,14	7,9[a]<br>±1,14	9,2[a]<br>±1,25	11,3[a]<br>±1,52	12,5[a]<br>±1,9	14,1[a]<br>±2,01	15,5[a]<br>±2,47	17,5[a]<br>±3,18	18,9[a]<br>±3,25	21,1[a]<br>±4,16	22,5[a]<br>±3,98	24,3<br>±4,58	25,7[a]<br>±5,71	27,5[a]<br>±6,14

Table S19: Growth dynamics of *Heterobasidion parviporum* mycelium measured *in vitro* at 12 h intervals in cultures treated with 1–4 mL of acetone extract from *Pseudevernia furfuracea* incorporated into liquid Hansen's medium before solidification and incubated at 28 °C.

Statistically significant differences compared to the control sample (0 mL) are shown in red.

a - statistically significant differences between compared pairs of cultures supplemented with various doses of the extract.

Heterobasidion parviporum*; extract from *P. furfuracea																			
	Average mycelium diameter [mm] at a given measurement hour<br>±standard deviation in sample [mm]																		
	12	24	36	48	60	72	84	96	108	120	132	144	156	168	180	192	204	216	228
Control sample<br>0 mL	5<br>±0	5<br>±0	5,5<br>±0,87	7,8<br>±1,35	10,5<br>±1,12	12,9<br>±1,67	15,8<br>±2,02	19<br>±2,26	22,5<br>±2,69	26,3<br>±2,89	29,5<br>±3,22	33,3<br>±3,65	37,5<br>±4,09	40,9<br>±4,26	45,5<br>±4,97	49,9<br>±5,07	53,6<br>±5,96	58,4<br>±5,91	62,4<br>±6,1
1 mL extract	5<br>±0	5<br>±0	5<br>±0	5,9<br>±0,74	7,2<br>±0,57	8,2<br>±1,3	9,5<br>±1,5	10,9<br>±2,04	12,3[a]<br>±2,39	13,6[a]<br>±2,9	14,8[a]<br>±3,13	16,5[a]<br>±3,16	18,2[a]<br>±3,38	19,5[a]<br>±3,61	21,4[a]<br>±3,75	22,4[a]<br>±3,75	24,4[a]<br>±3,9	25,9[a]<br>±4,02	27,1[a]<br>±4,32
2 mL extract	5<br>±0	5<br>±0	5<br>±0	5,2<br>±0,45	5,4<br>±0,55	5,5<br>±0,55	5,6<br>±0,55	6<br>±0,71	6,7<br>±0,84	7,5<br>±1	8,2<br>±1,15	9,3<br>±1,15	10<br>±1,22	10,9<br>±1,34	12<br>±1,54	12,6<br>±1,39	13,1<br>±1,39	13,9<br>±1,56	14,4<br>±1,82
3 mL extract	5<br>±0	5<br>±0	5<br>±0	5<br>±0	5<br>±0	5<br>±0	5,1<br>±0,22	5,5<br>±0,71	5,6<br>±0,89	5,8<br>±1,15	6<br>±1,54	6,6<br>±1,47	7,4<br>±1,82	8,1<br>±1,75	8,7<br>±1,82	9,3<br>±1,6	9,9<br>±1,64	10,2<br>±1,4	10,6<br>±1,39
4 mL extract	5<br>±0	5<br>±0	5<br>±0	5<br>±0	5<br>±0	5<br>±0	5<br>±0	5<br>±0	5[a]<br>±0	5[a]<br>±0	5[a]<br>±0	5[a]<br>±0	5[a]<br>±0	5[a]<br>±0	5[a]<br>±0	5,3[a]<br>±0,45	5,9[a]<br>±0,22	6[a]<br>±0	6,7[a]<br>±0,45
